# Reconfigurable pH-Responsive DNA Origami Lattices

**DOI:** 10.1021/acsnano.3c03438

**Published:** 2023-05-31

**Authors:** Sofia Julin, Veikko Linko, Mauri A. Kostiainen

**Affiliations:** † Biohybrid Materials, Department of Bioproducts and Biosystems, Aalto University, P.O. Box 16100, 00076 Aalto, Finland; ‡ LIBER Center of Excellence, Aalto University, P.O. Box 16100, 00076 Aalto, Finland; § Institute of Technology, University of Tartu, Nooruse 1, 50411 Tartu, Estonia

**Keywords:** DNA nanotechnology, DNA origami, metal nanoparticles, DNA triplex, pH control, hierarchical self-assembly

## Abstract

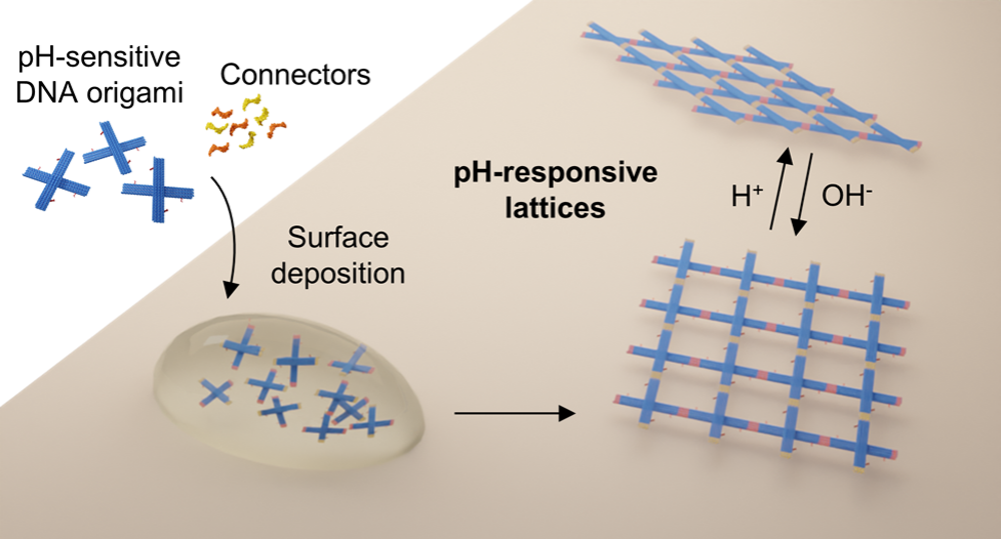

DNA nanotechnology enables straightforward fabrication of user-defined and nanometer-precise templates for a cornucopia of different uses. To date, most of these DNA assemblies have been static, but dynamic structures are increasingly coming into view. The programmability of DNA not only allows for encoding of the DNA object shape but also it may be equally used in defining the mechanism of action and the type of stimuli-responsiveness of the dynamic structures. However, these “robotic” features of DNA nanostructures are usually demonstrated for only small, discrete, and device-like objects rather than for collectively behaving higher-order systems. Here, we show how a large-scale, two-dimensional (2D) and pH-responsive DNA origami-based lattice can be assembled into two different configurations (“open” and “closed” states) on a mica substrate and further switched from one to the other distinct state upon a pH change of the surrounding solution. The control over these two configurations is achieved by equipping the arms of the lattice-forming DNA origami units with “pH-latches” that form Hoogsteen-type triplexes at low pH. In short, we demonstrate how the electrostatic control over the adhesion and mobility of the DNA origami units on the surface can be used both in the large lattice formation (with the help of directed polymerization) and in the conformational switching of the whole lattice. To further emphasize the feasibility of the method, we also demonstrate the formation of pH-responsive 2D gold nanoparticle lattices. We believe this work can bridge the nanometer-precise DNA origami templates and higher-order large-scale systems with the stimuli-induced dynamicity.

Recent advances in the field of nanotechnology have enabled the fabrication of a variety of nanoobjects with intriguing geometries and properties. However, for many applications, more complex, structurally well-defined nanomaterials in which the individual building blocks could interact with each other in a predefined manner would be highly desirable.^1−3^ Owing to the highly specific and predictable Watson–Crick base pairing, DNA-based nanostructures have proven to be feasible templates for constructing precise nanoscale arrangements.^4,5^ For this, particularly, the DNA origami technique allows for the production of a wide range of well-defined two- and three-dimensional (2D and 3D) DNA nanostructures with high complexity and addressability.^6−8^ The rapidly emerged DNA origami design software^9−11^ have further given rise to numerous sophisticated applications in nanomedicine,^12−15^ nanophotonics,^16,17^ nanoelectronics,^18^ and bottom-up nanofabrication.^19,20^

DNA origami is a versatile method, and therefore, it has also been used to construct large-scale hierarchical assemblies.^21−24^ These DNA origami-based lattices could also serve as templates for controlling and directing the spatial arrangements of other compounds, as shown for example by creating increasingly complex metal nanoparticle lattices using DNA origami frameworks.^25,26^ Thus, far, most of such research has been focused on static assemblies; however, getting inspired by nature, the interests are increasingly shifting toward dynamic structures that undergo conformational changes in response to external stimuli, such as pH, salt concentration, light, or temperature.^27^ Apart from a very few examples,^28−31^ the use of DNA origami for the construction of dynamic 2D and 3D lattices has been rather limited. Nevertheless, the library of already demonstrated small dynamic DNA-based devices^32,33^ suggests that the DNA origami method could also be harnessed in building larger dynamic lattices and other highly ordered assemblies.^34^

In this work, we created a dynamic 2D DNA origami lattice that changes its configuration in response to the pH of the surrounding solution. For that, we designed a pliers-like DNA origami unit that serves as the basic building block of the lattice and that can be readily switched between an open “+”-shaped and a closed “X”-shaped state upon a pH change. The controlled dynamicity is achieved using pH-sensitive “latches”, whose counterparts are positioned at the opposite arms of the unit (Figure 1a). These particular “pH-latches” are based on the pH-dependent, Hoogsteen-type DNA triplex formation,^35,36^ but it is noteworthy to mention that there also exist other pH-responsive constructs that could be equally implemented, such as the i-motif.^37^ First, we characterized the plain unit and its dynamic behavior by agarose gel electrophoresis (AGE) and transmission electron microscopy (TEM). By introducing “connector oligonucleotides”, the units were selectively linked together, and subsequently, we were able to increase the complexity of our system. This was shown by assembling DNA origami dimers, one-dimensional (1D) DNA origami arrays (chains), and ultimately reconfigurable pH-sensitive 2D DNA origami lattices. Furthermore, the developed lattices could also serve as templates for other nanoscale compounds, which we demonstrated here by assembling dynamic pH-responsive 2D gold nanoparticle (AuNP) lattices.

**Figure 1:**
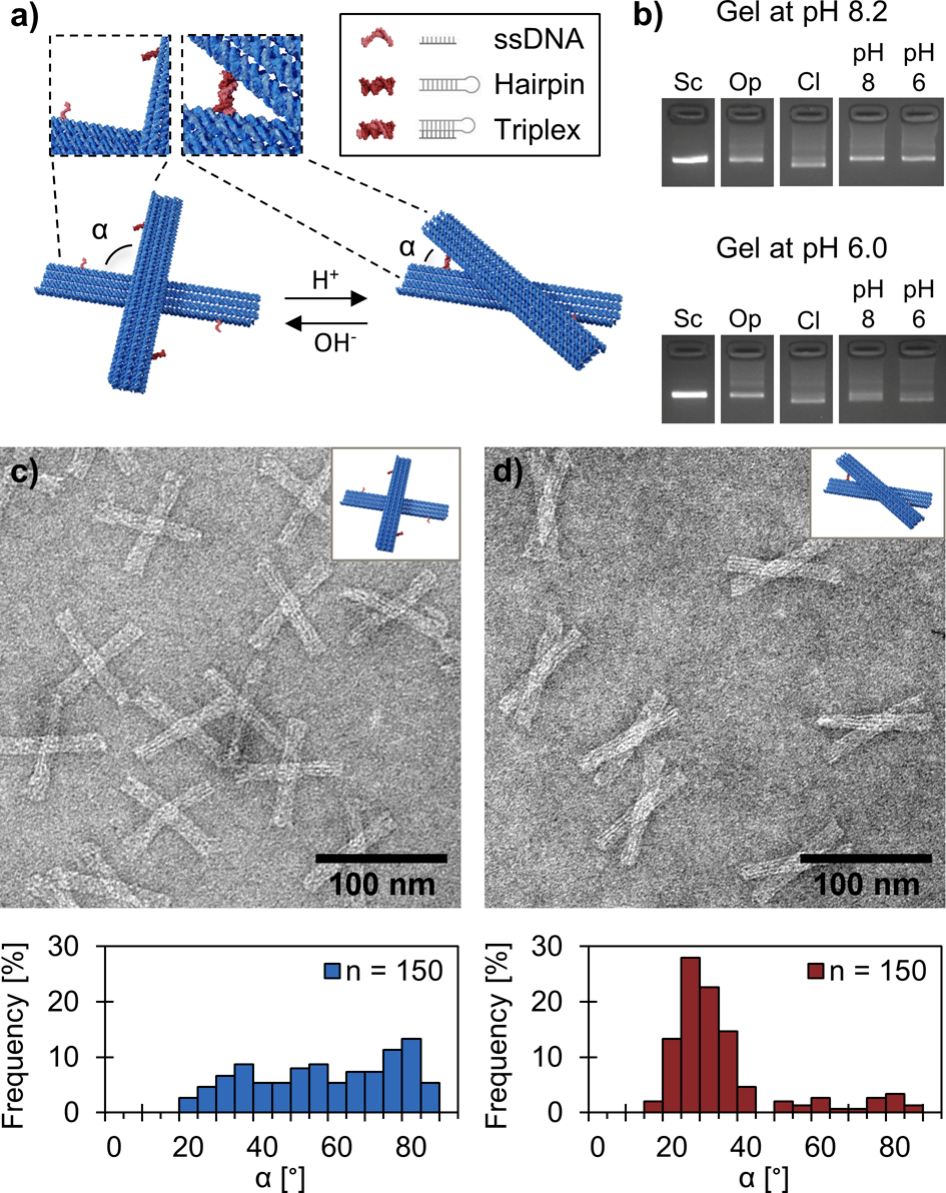
Design and characterization of the reconfigurable pH-sensitive DNA origami unit. (a) DNA origami unit contains two bar-like arms (86 nm × 12 nm × 6 nm) that are connected through a pivot (two DNA scaffold crossovers). The arms are equipped with two latches that consist of a 20-bp hairpin and a complementary 20-nt ssDNA counterpart. These two latch counterparts will form a DNA triplex when the solution pH is below the transition p*K*_a_ value (∼7.2), and thus, the closing of the latches locks the arms of the unit at a fixed vertex angle, α ≈ 30°. Increasing the pH above the p*K*_a_ will open the latches and let the arms move freely between α ≈ 20–90°. (b) Analysis of different DNA origami units by agarose gel electrophoresis (AGE) at pH 8.2 (top panel) and pH 6.0 (bottom panel). The samples in the gel are scaffold (Sc), permanently open unit (Op), permanently closed unit (Cl), and unit with pH latches at pH 8.2 (pH 8, initially open) and pH 6.0 (pH 6, initially closed). Transmission electron microscopy (TEM) images of the units with pH latches at (c) pH 8.2 and (d) pH 6.0. Both TEM images are negatively stained with 2% (w/v) uranyl formate. The bottom panel shows the distribution of the angle, α, between the two arms of the unit. The number of individual structures analyzed for each sample is *n* = 150.

## Results and Discussion

### Design and Characterization of the Reconfigurable pH-Responsive DNA Origami Unit

To assemble the pH-responsive and dynamic lattice, we first constructed and characterized the pH-sensitive DNA origami unit, the basic building block of the lattice. The pliers-like DNA origami unit consists of two bar-shaped arms (86 nm × 12 nm × 6 nm) that are connected to each other through the pivot which is two single-stranded DNA (ssDNA) scaffold crossovers (analogous to the Holliday junction) (Figure 1a). The unit is designed with two rationally engineered pH-sensitive “latches”. Therefore, depending on the pH of the surrounding solution, the unit may adopt either an open (arms rotate freely with respect to each other, and thus the observed vertex angle between the arms varies from α ≈ 20° to α ≈ 90°) or a closed configuration (vertex angle α ≈ 30°). In more detail, the latches are staple-strand extensions and consist of two counterparts positioned on different arms of the unit: a hairpin with a 20-base pair (bp) double-stranded DNA (dsDNA) region and a complementary 20-nucleotide (nt) ssDNA sequence. At high pH, the hairpin and the ssDNA do not interact with each other, thus allowing for free rotation of the arms. At low pH, for one, these two counterparts can form a parallel DNA triplex through Hoogsteen interactions, which locks the two arms at a fixed position. Both pH latches have different base sequences but an identical T-A·T base content of 60%, which ensures that both latches have a transition pH value of p*K*_a_ ∼ 7.2 and thus they will open/close at the same pH.^38^ However, the pH range at which the opening/closing takes place can be rationally tuned by adjusting the T-A·T base content of the latch sequences.^35,39^ In addition to the pH-sensitive unit, we also designed and prepared two control units: a permanently open unit with no latch sequences (Op) and a permanently closed unit (Cl), in which the pH-sensitive latch sequences have been replaced with complementary ssDNA overhangs.

To confirm both the correct folding of the units and the functionality of the pH-sensitive latches, poly-T passivated DNA origami units (8-nt polythymine extensions at each helix to avoid end-to-end stacking) were first analyzed by agarose gel electrophoresis (AGE) (Figure 1b and Figure S2). The closed unit is more compact than the open unit and therefore the closed unit exhibits a higher electrophoretic mobility in the gel. This allows for separation of these two configurations by AGE. The first gel was run at pH 8.2 (Figure 1b, top panel), which is above the p*K*_a_ value, and thus, it was also expected that samples prepared at pH 8.2 (initially open) and at pH 6.0 (initially closed) will both adapt the open configuration. This is indeed the case, as the both samples exhibit equal mobility which further matches the mobility of the permanently open (Op) control sample. The second gel (Figure 1b, bottom panel), was run at pH 6.0, which is well below the p*K*_a_ value. Here, the (initially closed) sample at pH 6.0 remains predominantly in its closed configuration, while the (initially open) sample prepared at pH 8.2 shows a slightly broader band. This indicates that the sample is a blend of both open and closed configurations due to the slow closing kinetics of the initially open unit.^38,39^ The opening kinetics is faster, and therefore, the initially closed unit will swiftly open in the pH 8.2 gel, resulting in a clear and narrow band.

In addition to AGE, we also used TEM to characterize the DNA origami units at both pH 8.2 and pH 6.0. In both cases, TEM reveals distinct, correctly folded units (Figure 1c,d and Figures S3 and S4). At pH 8.2, the unit equipped with pH-sensitive latches adapted the open configuration with a wide (α ≈ 20–90°) and flat vertex angle distribution (Figure 1c). At pH 6.0, on the other hand, most of the units adapted the closed configuration with an vertex angle of α ≈ 30° (∼75% of the units have vertex angles within 20–40°) (Figure 1d). Despite this pronounced and narrow vertex angle distribution, both TEM and AGE analysis additionally reveal that a small fraction of the pH-sensitive units still remains at the open configuration at pH 6.0. The same trend was also observed for a pH-sensitive unit variant with different latch configurations, thus allowing for closing of the arms in the opposite direction (Figure S5) and the permanently closed control unit (at both pH 8.2 and pH 6.0, see Figures S6 and S7). The effect was even more pronounced at low cation concentrations (see Figure S1), indicating that the electrostatic repulsion between the two arms is strong enough to prevent some units from closing. Nevertheless, the observed closing yield is in good agreement with previously reported closing efficiencies for similar pH-responsive DNA origami structures.^40^

### Selective Assembly of DNA Origami Dimers

**Figure 2:**
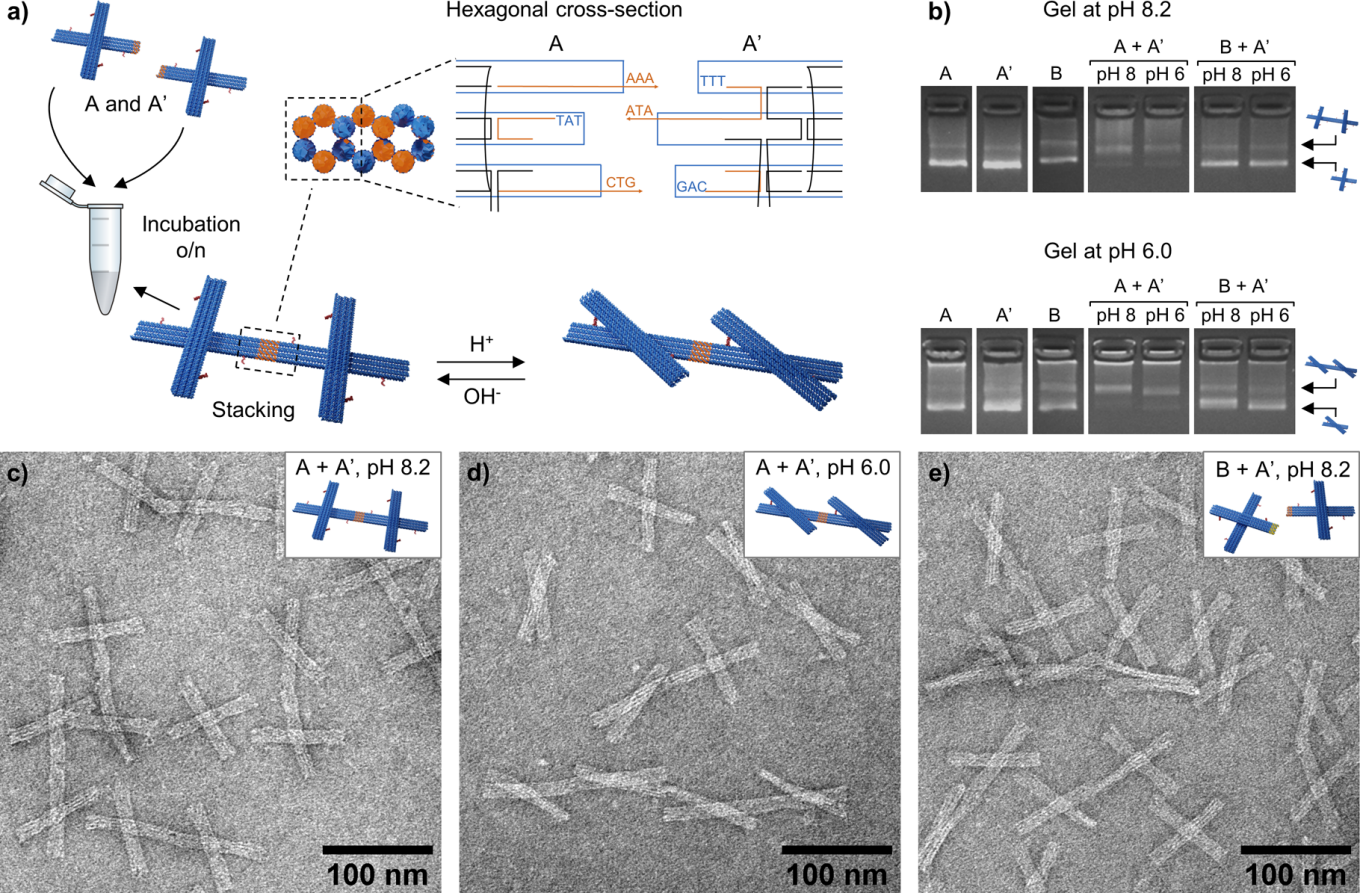
Formation of dynamic DNA origami dimers. (a) Dimers are formed by mixing equimolar amounts of both units (folded separately). The DNA origami units are selectively linked together by bridging the side scaffold loops with connector oligonucleotides. To connect the scaffold loops, seven of the connector oligonucleotides have a 3-nt overhang (in the 3′ end) complementary to the scaffold sequence on the opposite end of the arm. (b) Characterization of the dimer formation by AGE at pH 8.2 (top panel) and pH 6.0 (bottom panel). If not otherwise specified, the pH of the samples are 8.2 in the top gel and 6.0 in the bottom gel. TEM images of (c) dimers formed at pH 8.2 by combining A and A′ units (*c* = 5.7 nM), (d) the same dimer solution as in (c) (A and A′ units, *c* = 5.4 nM) after the pH has been decreased to 6.0 with acetic acid, and (e) a mixture of B and A′ units (*c* = 5.4 nM). These units do not have matching connector oligonucleotides, and therefore, no dimers are formed. The samples in TEM images are negatively stained with 2% (w/v) uranyl formate.

For the lattice formation, it is crucial that the units are connected together in a programmable fashion without undesired interconnection of the top arm and the bottom arm that are located in different planes. In order to selectively connect only specific ends of the arms, we designed “connector oligonucleotides” for seven helices in each of the two arms (Figure 2a and Figure S45). To further minimize the undesired interactions between the two arms, the connector oligonucleotides were arranged in different patterns for the top and the bottom arms of the unit, while the rest of the helix-ends remained untouched (blue helices in Figure 2a cross-section). In total, 14 strands of the connector oligonucleotides (7 per each arm, 4 at one end and 3 at the other) contain a 3-nt long protruding 3′-end-overhang. Each overhang is complementary to a 3-nt long scaffold sequence, which is located in the same helix but at the opposite site of the arm (3 and 4 recession sites at the opposite edges of the arm). Therefore, these interlocking complementary sequences can efficiently bridge the side scaffold loops of these two adjacent DNA origami units.^41^ The combination of short hybridizing sequences and shape complementarity provides the needed specificity for correctly joining the units together; however, the interactions are still weak enough to allow for rearrangements between the units and thus to help avoiding misaligned lattice formation.^42^

To demonstrate the selectivity of the connector oligonucleotides, we, as a proof of concept, prepared different versions of pH-responsive DNA origami dimers (Figure 2a and Figures S9–S17). The two units (marked with A and A′ if the connector oligonucleotides are in the bottom arm) were folded and purified from excess staple strands in separate batches, after which the dimers were formed by mixing equimolar amounts of both units. In order to prevent multimerization of the units, the interfaces of the arm ends not involved in dimer formation were poly-T-passivated (8-nt long polythymine overhangs). AGE revealed that the band corresponding to the single units almost completely vanished in the dimer mixture, whereas another band with lower electrophoretic mobility appeared in the gel, indicating a successful dimerization (Figure 2b and Figures S9 and S13). Importantly, a control sample with mismatching units (unit B with connectors in top arm combined with unit A′) did not form any dimers, demonstrating that our strategy to connect the units is indeed highly selective. To further confirm that the two units interact with each other correctly, we used TEM to visualize the formed dimers. The TEM images of the dimers that were assembled at pH 8.2 show, as expected, perfectly aligned and well-defined DNA origami dimers with the arms open (Figure 2c and Figures S10 and S11). Furthermore, by adding acetic acid to this dimer solution, the arms of the dimer could be locked into the closed configuration (Figure 2d and Figures S10 and S11). Equally, the dimers could be formed from the units initially at the closed state at pH 6.0, after which the arms could be released again by increasing the pH with sodium hydroxide (Figures S13–S15). As indicated above, the B and A′ units neither have the required shape complementarity nor the matching sequences, and therefore, only discrete, unconnected DNA origami units were observed in TEM (Figure 2e and Figures S12 and S16).

### Formation of 1D Arrays Using the DNA Origami Unit

**Figure 3:**
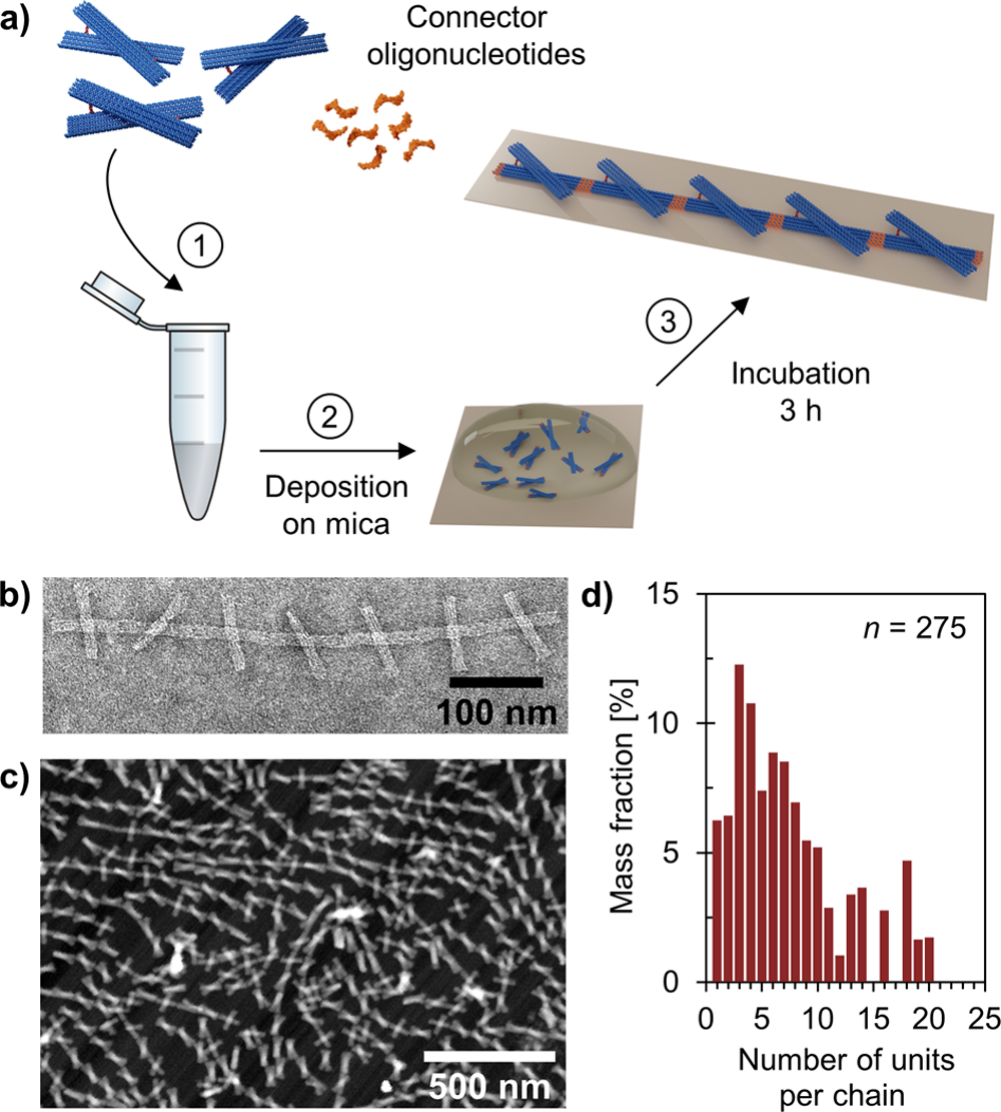
Formation of one-dimensional (1D) arrays using the DNA origami unit. (a) Polymerization of the units into chains is initiated by the addition of connector oligonucleotides. For the surface-mediated assembly, the mixture is immediately deposited onto a mica substrate. (b) TEM image of a negatively stained linear 1D array formed in solution at pH 8.2 (25 h incubation at room temperature, *c*_unit_ = 10.0 nM, but sample diluted 1:2 in 1× FOB (1× TAE, 20 mM MgCl_2_, 5 mM NaCl) before deposited onto the TEM grid). (c) Atomic force microscopy (AFM) image of DNA origami chains formed on a mica substrate at pH 6.0 (3 h incubation). (d) Observed chain length distribution for the 1D arrays assembled on a mica substrate at pH 6.0 (determined from AFM images).

To further explore the possibility of using the DNA origami unit for the construction of large-scale lattices, we formed 1D arrays using the DNA origami unit. To this end, we prepared a unit with the polymerizing connector oligonucleotides on the bottom arm (A and A′ interactions) and fully poly-T-passivated interfaces on the top arm. To avoid undesired multimerization and formation of kinetically trapped configurations during the folding, the unit was prepared without the connector oligonucleotides. The polymerization of the units into linear arrays was initiated in a subsequent step by adding connector oligonucleotides in 10-fold excess to units that were earlier purified from the excess staple strands used in folding (Figure 3a, step 1). Initially, the assembly was carried out in solution by incubating the sample mixture at room temperature for at least 24 h. Although we recognized correctly formed linear chains when imaging the sample by TEM (Figure 3b and Figures S18 and S19), the tendency of the sample to form highly entangled structures set limitations to the analysis of the chain formation.

As an alternative to the solution-phase formation, we also assembled the DNA origami chains on a mica substrate at the solid–liquid interface. The interface restricts the movement of the units to the 2D plane and may thus provide additional control of the lattice formation and growth.^34,43^ For the surface-assisted assembly, the units and the connector oligonucleotides were mixed together in a buffer supplemented with MgCl_2_ and NaCl and immediately after that deposited onto a mica substrate (Figure 3a). Linear arrays were grown at both pH 6.0 (Figure 3c and Figure S20) and pH 8.2 (Figures S21 and S22), and in both cases, discrete chains of various lengths were formed. Nineteen percent of the units assembled into >1 μm long chains (>11 units), while the majority of them formed chains of 3–10 units (pH 6.0, *n* = 275) (Figure 3d). This is also in line with the previously reported chain lengths for similar linear DNA origami arrays.^44^

### Assembly of pH-Responsive and Reconfigurable 2D DNA Origami Lattices

**Figure 4:**
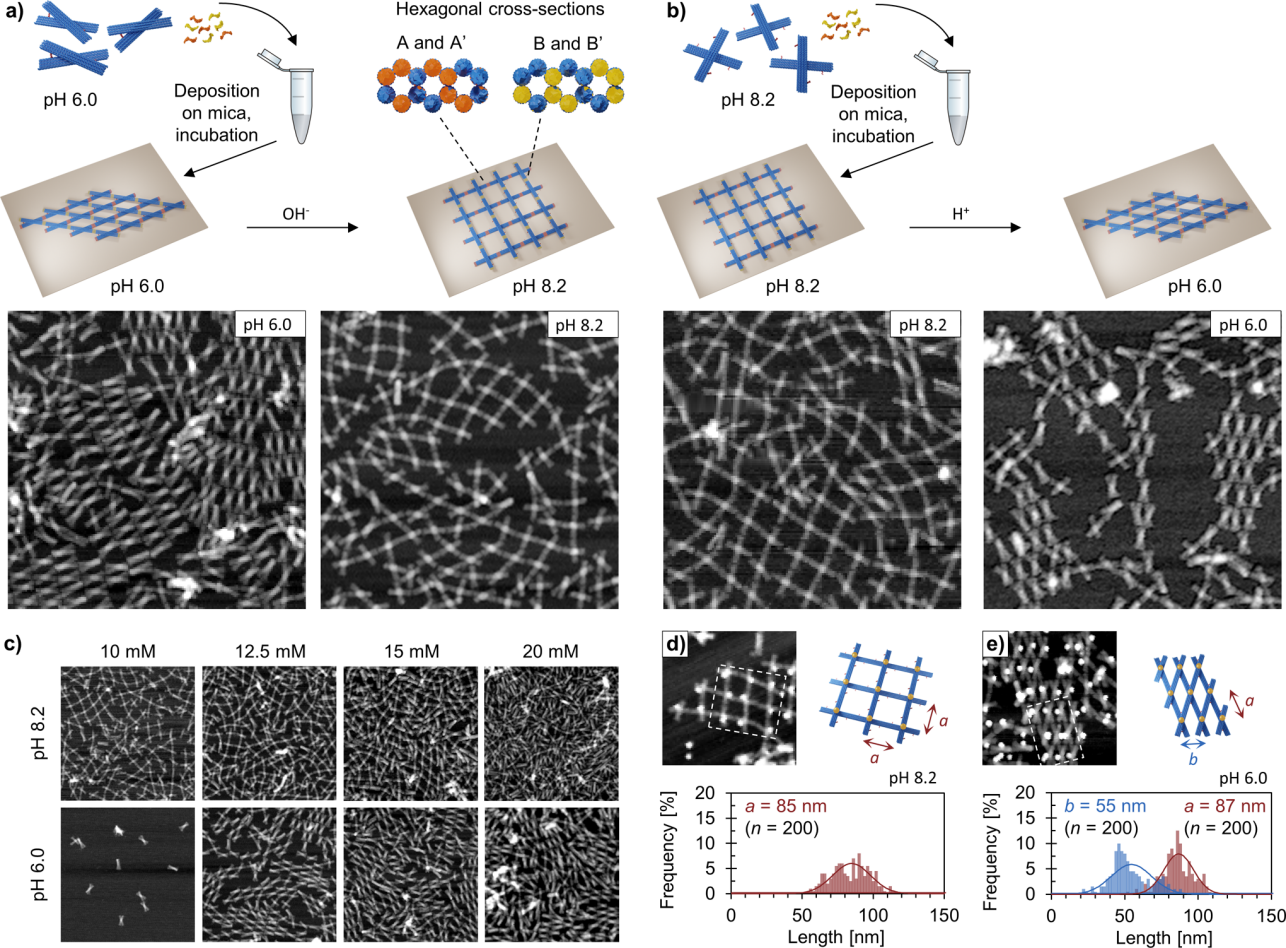
Assembly of pH-responsive and reconfigurable two-dimensional (2D) DNA origami lattices. (a and b) Connector oligonucleotides for both arms of the unit initiate the assembly of a 2D lattice on a mica substrate. The formed lattice could adapt either an open or a closed configuration depending on the pH of the surrounding solution. The reconfigurable lattice could be expanded or squeezed also after the initial assembly by increasing or decreasing the pH. AFM images (1 μm × 1 μm) of the different lattice configurations are shown below the schematics. (c) AFM images (1 μm × 1 μm) of the 2D lattice formation at different Mg^2+^ concentrations. The Na^+^ concentration is kept constant at 75 mM, and the assembly time is 3 h. The DNA origami lattice could guide gold nanoparticles (AuNPs) into either (d) a square lattice at pH 8.2 or (e) a oblique lattice at pH 6.0. The top panel shows an AFM image (500 nm × 500 nm) of the AuNP lattice, and the area marked with dotted lines is schematically presented next to the image. The bottom panel show the observed lattice constant distributions for the formed AuNP lattice (determined from the AFM images). The AuNP lattices are assembled during 3 h. In (a), (b), (d), and (e), the Mg^2+^ concentration is 10 mM for lattices at pH 8.2 and 12.5 mM for lattices at pH 6.0.

By introducing connector oligonucleotides on both the bottom and the top arms of the unit (A and A′ interactions as well as B and B′ interactions), we constructed dynamic 2D lattices (Figure 4a,b). The two pH-sensitive conformations of the unit allow the lattice to adopt either an open or a closed configuration depending on the pH of the assembly solution. The 2D lattices were assembled directly onto the mica substrate by employing a previously established protocol^24^ that we further developed and optimized for our system. For successful formation of large hierarchical DNA origami assemblies on mica, the electrostatic interactions between the DNA origami and the surface have to be carefully controlled, which is usually accomplished by tuning the relative amounts of Na^+^ and Mg^2+^ in the assembly buffer.^43,45^ The divalent Mg^2+^ ions mediate the DNA origami adsorption onto mica by forming salt bridges, whereas the competitive Na^+^ ions weaken these interactions and enhance the DNA origami mobility on the surface. Depending on the assembly pH, we observed a clear difference in the DNA origami adsorption, which also affected the lattice growth. Therefore, we investigated the influence of the Mg^2+^ concentration on lattice formation on the mica substrate during 3 h by keeping the Na^+^ concentration constant at 75 mM (Figure 4c and Figures S23 and S24). At pH 8.2, the optimum Mg^2+^ concentration was found to be 10 mM, which is well in agreement with previously optimized conditions.^46^ At pH 6.0, for one, the electrostatic interactions were noticeably weaker and a Mg^2+^ concentration of 12.5 mM was needed to obtain sufficient DNA origami adsorption for the subsequent lattice growth. The observed pH-dependent difference in the required Mg^2+^ concentration may be explained by silicate protonation and thus a reduced surface charge of mica at low pH. In addition, increasing the Mg^2+^ concentrations of the assembly solution beyond these optimized values results in high DNA origami adsorption and low DNA origami mobility on the surface, which considerably decrease the lattice order.

Depending on the assembly pH, the DNA origami lattice has two clearly distinguishable configurations (Figures 4a,b, bottom left). At pH 8.2, the unit will adapt the open configuration and the formed lattice will be in an expanded state. At pH 6.0, on the other hand, the units are predominantly in the closed configuration, and therefore, a more compact lattice is formed. Nonetheless, in both cases, the obtained lattice is polycrystalline and composed of smaller crystalline domains of various sizes in close proximity to each other. The order and the size of the crystal domains correlate with the assembly duration, and therefore, the crystal growth could be considerably improved by increasing the assembly time (Figures S25–S28). The crystal domains are generally also larger at pH 6.0, which could be explained by the enhanced rigidity of the unit when the arms are tied together and thus not able to rotate freely. Moreover, replacing Mg^2+^ with Ca^2+^ has been shown to enhance the lattice order for closed-packed lattices of symmetric DNA origami units that do not bind to each other via basepairing,^47^ but for our system, this replacement had no significant effect (Figure S29).

Thus far, most of the reported DNA origami-based frameworks have been static, meaning that their lattice parameters have been fixed once they have been assembled. However, approaches allowing a stimuli-induced dynamic symmetry conversion after the assembly would be highly desirable. Therefore, we next studied whether our assembled pH-responsive and reconfigurable lattices could be readily expanded and squeezed by simply increasing or decreasing the pH. For these experiments, we first assembled the lattices on the mica surface for 5 h at pH 8.2 or 6.0, washed away weakly interacting and unbound assemblies, deposited a different buffer solution with lower/higher pH, and incubated for additional 2 h (pH increase from 6.0 to 8.2) or 20 h (pH decrease from 8.2 to 6.0). When the pH was increased from 6.0 to 8.2, a clear change from the closed state toward the open lattice configuration was observed (Figure 4a, bottom right and Figures S30–S32), indicating that the formed lattices are rather mobile on the surface. Closing of the lattice after assembly, (pH decrease from 8.2 to 6.0), for one, required much longer time, and the overall change in the lattice configuration was not as pronounced as in the case of opening the lattice (Figure 4b, bottom right and Figures S33–S35). Interestingly, we also observed that, as long as the lattices were not attached to the mica substrate with NiCl_2_, the once dried lattices (for AFM imaging) could be rehydrated and their configuration altered by increasing or decreasing the pH (Figures S36–S40). This further demonstrates that the lattices are mobile enough on the surface to rearrange also after the initial assembly.

### Assembly of DNA-Templated, pH-Responsive, and Reconfigurable 2D AuNP Lattices

It is known that spatially well-defined arrangements of metal nanoparticles possess intriguing optical, plasmonic, electronic, and magnetic properties,^2^ but fabrication of highly ordered dynamic nanoparticle lattices is rather challenging. As already mentioned, programmable and modular DNA-based structures are suitable templates for guiding nanoparticles into complex, mostly static lattices using either DNA hybridization^21,25,26^ or electrostatic interactions.^48^ In order to demonstrate that our pH-sensitive lattice could be used as a template to create reconfigurable nanoparticle lattices, we modified the DNA origami unit by adding an anchoring site for an oligonucleotide-coated gold nanoparticle (AuNP, 10 nm in diameter) in the middle of the unit (Figure S8). AFM images of the prepared lattices show, as expected, two distinct lattice configurations depending on the assembly pH or unit used; a 2D square lattice at pH 8.2 (Figure 4d and Figure S41) and a 2D oblique lattice at pH 6.0 (Figure 4e and Figure S42) or if a permanently closed unit is used (Figure S43). Furthermore, the average lattice constants determined by AFM are *a* = 85 ± 13 nm for the square lattice and *a* = 87 ± 10 nm, *b* = 55 ± 14 nm for the oblique lattice. The highest frequency was observed for *a* = 90–92 nm for the square lattice and *a* = 86–88 nm and *b* = 44–46 nm for the oblique lattice. The DNA origami unit is rather flexible at pH 8.2, and taking that into account, the observed lattice constants are well in agreement with the theoretical ones (*a* = 86 nm (both for square and oblique lattices) and *b* = 45 nm, assuming a vertex angle of 30°).

## Conclusions

In this work, we have presented a strategy for constructing pH-responsive and dynamically reconfigurable lattices using DNA origami as the building block. The pH-responsiveness of the lattice is achieved by equipping the arms of the pliers-like, lattice-forming DNA origami unit with pH latches that form Hoogsteen-type of triplexes in low pH. Therefore, the unit could rapidly switch between an open “+”-shaped and a closed “X”-shaped configuration upon a pH change. Nevertheless, the high level of programmability of the DNA origami would equally enable other stimuli-responsive elements, such as photoresponsive molecules^49^ and thermoresponsive polymers,^50^ to be implemented into the basic building block of the lattice, thus allowing reconfigurable lattices that undergo conformational changes in response to different external stimuli. Furthermore, the high addressability of DNA origami allows not only AuNPs (as demonstrated here) but also a wide variety of other compounds to be precisely positioned onto DNA origami frameworks. Therefore, we believe that our demonstrated system as well as other recently reported reconfigurable DNA-based lattices^28−31,51,52^ will contribute to the development of more sophisticated stimuli-responsive and functional materials in future.

## Methods

### Design and Preparation of the pH-Responsive DNA Origami Unit

The pH-responsive DNA origami unit was designed on a honeycomb lattice using caDNAno v 2.2.0,^53^ and its three-dimensional shape was predicted using the CanDo software.^54,55^ The caDNAno design for the unit is shown in Figures S44 and S45, and the staple strands for the different versions of the unit are listed in Tables S2–S9.

The DNA origami units were folded in a one-pot reaction in either 50 or 100 μL quantities by mixing the circular p7249 scaffold (final concentration of 20 nM) with 7.5× excess of staple strands in a folding buffer (FOB) containing 1× Tris-acetate-EDTA (TAE) buffer, 20 mM MgCl_2_, and 5 mM NaCl. The folding reaction mixture was thermally annealed from 75 to 27 °C in a ProFlex PCR system or a G-storm G1 Thermal Cycler using the following annealing program: (1) Cooling from 75 to 70 °C at a rate of −0.2 °C/8 s; (2) cooling from 70 to 60 °C at a rate of −0.1 °C/8 s; (3) cooling from 60 to 27 °C at a rate of −0.1 °C/2 min; and (4) cooled down to 20 or 12 °C and stored at this temperature until the program was manually stopped.

The excess staple strands were removed from the folded DNA origami structures using a polyethylene glycol (PEG) precipitation method.^56^ First, the DNA origami solution was diluted 4-fold with 1× FOB, after which the solution was thoroughly mixed 1:1 with PEG precipitation buffer (15% (w/v) PEG 8000, 1× TAE, 505 mM NaCl). The mixture was centrifuged at 14 000 *g* for 30 min at room temperature using an Eppendorf 5424R microcentrifuge, the supernatant was carefully removed, and the DNA origami pellet was resuspendended in 1× FOB (either at pH 8.2 or 6.0) to the original reaction volume. To dissolve the pellet, the DNA origami solution was incubated at 30 °C overnight under continuous shaking at 600 rpm using an Eppendorf Thermomixer C. The DNA origami concentration was estimated as described in Section 2.1 in the Supporting Information.

### Dimer Formation

The DNA origami dimers were formed by mixing equimolar amounts of PEG-purified DNA origami units (to a final concentration of 5.7 nM) in 1× FOB (either at pH 8.2 or 6.0). To allow for the formation of dimers, the samples were incubated at room temperature for at least 22 h. The pH of the dimer solution was decreased/increased by adding 1.5 μL of 0.5 M acetic acid or 0.5 M sodium hydroxide to 30 μL of dimer solution and incubating at room temperature for at least additional 23 h.

### 1D Array Formation in Solution

For the assembly of DNA origami chains in solution, PEG-purified DNA origami units (final concentrations of 2.0, 5.0, or 10.0 nM) were mixed with 10-fold excess of connector oligonucleotides in 1× FOB (either at pH 8.2 or 6.0). To allow for polymerization, the samples were incubated at room temperature for at least 24 h.

### 1D and 2D Lattice Assembly on Mica

The lattice assembly on mica was mainly carried out following a procedure previously described by Xin *et al*.^24^ For the deposition, PEG purified DNA origami units (final concentration of 2.0 nM) were mixed with 10-fold excess of connector oligonucleotides in a buffer (at either pH 8.2 or 6.0) containing 1× TAE supplemented with MgCl_2_ (10–20 mM depending on the sample) and 75 mM NaCl. The DNA origami sample mixture (120 μL) was evenly deposited onto a freshly cleaved mica surface (15 mm × 15 mm, grade V1, Electron Microscopy Sciences) and incubated covered at room temperature for 3–24 h. After the incubation, the mica surface was rinsed 5 times with 100 μL of 1× TAE supplemented with MgCl_2_ (same MgCl_2_ concentration and pH as in the sample solution). Immediately after the washing step, 120 μL of 1× TAE containing 10 mM NiCl_2_ was deposited on the mica surface and incubated covered for 1 h. After the incubation, the mica surface was rinsed 6 times with 100 μL of deionized water, after which the sample was dried thoroughly using a nitrogen gas stream.

### pH-Responsiveness of Assembled 2D Lattices

To demonstrate that the lattice is pH-responsive and reconfigurable also after the initial assembly, the lattices were assembled as described above. After the initial assembly, the mica surface was rinsed 3–5 times with 100 μL of 1× TAE, 10 mM MgCl_2_ at pH 8.2 (for the lattice assembled at pH 8.2) or 100 μL of 1× TAE, 12.5 mM MgCl_2_ at pH 6.0 (for the lattice assembled at pH 6.0). In order to decrease the pH, 120 μL of 1× TAE, 12.5 mM MgCl_2_, and 75 mM NaCl at pH 6.0 was deposited on the lattice assembled at pH 8.2 and incubated for 20 h under a cover. Similarly, in order to increase the pH, 120 μL of 1× TAE, 10 mM MgCl_2_, and 75 mM NaCl at pH 8.2 was deposited on the lattice assembled at pH 6.0 and incubated for 2 h under a cover. After the incubation, the mica surface was rinsed 3–5 times with 100 μL of 1× TAE, 12.5 mM MgCl_2_ at pH 6.0 (for the lattice changed to pH 6.0) or 100 μL of 1× TAE, 10 mM MgCl_2_ at pH 8.2 (for the lattice changed to pH 8.2). Immediately after the second washing step, 120 μL of 1× TAE, 10 mM NiCl_2_ was deposited on the mica surface and incubated for 1 h under a cover. After the incubation, the mica surface was rinsed 6 times with 100 μL of deionized water, after which the sample was dried thoroughly using a nitrogen gas stream.

### Preparation of DNA-Functionalized AuNPs and AuNP-Conjugated DNA Origami Units

The DNA-functionalized AuNPs were mainly prepared as described previously.^38^ If not stated otherwise, all the steps of the DNA-functionalization of the AuNPs were carried out at 40 °C under constant shaking at 600 rpm using an Eppendorf Thermomixer C. First, 80 μL of citrate-stabilized AuNPs (10 nm in diameter, upconcentrated to 50 nM) was incubated with 1.6 μL of 1% (w/v) sodium dodecyl sulfate (SDS) solution for 20 min. Next, 16 μL of thiolated oligonucleotides (*c* = 100 μM, see Table S7 for the sequence) was added and the mixture was incubated for additional 30 min, after which a salt-aging process was carried out. First, 0.8 μL of 2.5 M NaCl was added every 5 min (6 times), followed by 1.6 μL of 2.5 M NaCl every 5 min (6 times), 3.2 μL of 2.5 M NaCl every 5 min (5 times), and 2.0 μL of 2.5 M NaCl (once). After the salt-aging, 120 μL of 1× FOB (1× TAE buffer, 20 mM MgCl_2_, 5 mM NaCl) supplemented with 0.02% (w/v) SDS was added, and the mixture was incubated for 60 min. The temperature was decreased to 20 °C, after which the incubation continued overnight (constant shaking at 600 rpm).

Before the conjugation to the DNA origami unit, the DNA-functionalized AuNPs were purified from excess thiolated oligonucleotides using spin-filtration at room temperature (Amicon Ultra 100 kDa MWCO centrifugal filter, EMD Millipore). The filter was washed with 200 μL of 1× FOB with 0.02% (w/v) SDS (14 000*g*, 5 min) before use. DNA-functionalized AuNPs (260–400 μL per addition, in total 1260 μL) were added to the filter unit, and after each addition, the unit was centrifuged at 14 000 *g* for 10 min using an Eppendorf microcentrifuge 5424R. Finally, the DNA-functionalized AuNPs were washed 3 times by adding 200 μL of 1× FOB with 0.02% (w/v) SDS and centrifuging at 14 000 *g* for 10 min. The DNA-functionalized AuNPs were recovered by inverting the filter unit and centrifuging at 2000*g* for 2.5 min.

The DNA origami unit has a position for AuNP attachment in the middle of the unit (see Table S7 for the sequences of the attachment strands). For the conjugation, 7.5× excess of DNA-functionalized AuNPs was mixed with PEG purified DNA origami units (final concentration of 7.5 nM in the conjugation mixture) in 1× FOB supplemented with 0.01% (w/v) SDS. To increase the attachment yield, the mixture was thermally annealed from 40 to 20 °C at a rate of −0.1 °C/min using a ProFlex PCR system.

Before the lattice assembly on mica, PEG precipitation^57^ was used to remove the SDS and some of the free AuNPs from the solution with AuNP-conjugated DNA origami units. Fifty microliters of AuNP-conjugated DNA origami units was mixed with 12.5 μL of PEG precipitation buffer (17.5% (w/v) PEG 8000, 1× TAE, 10 mM MgCl_2_, 500 mM NaCl). The mixture was incubated at 4 °C for 10 min before being centrifuged at 12 600 *g* at 4 °C for 30 min. The supernatant was carefully removed, after which the pellet was resuspended in 50 μL of 1× FOB at either pH 8.2 or 6.0. The solution was incubated at room temperature overnight before being deposited on mica. The lattice assembly was done as described above and the composition of the used buffer was the same, but the DNA origami concentration was slightly higher (2.0–3.0 nM, calculated based on the concentration in the conjugation step, assuming no loss during the PEG precipitation).

### Agarose Gel Electrophoresis

Agarose gel electrophoresis was used to analyze the folding of the DNA origami unit as well as the formation of DNA origami dimers. A 2% (w/v) agarose gel was prepared in 1× TAE buffer containing 11 mM MgCl_2_ for the gel at pH 8.2, whereas the 2% (w/v) agarose gel was prepared in 45 mM MES and 25 mM Tris containing 11 mM MgCl_2_ for the gel at pH 6.0. Both gels were stained with ethidium bromide (final concentration of 0.46 μg mL^–1^). Depending on the sample and the type of gel, the sample volume was 10–18 μL and the DNA origami concentration was 11.1/15.0 nM (DNA origami units) or 5.4/5.7 nM (DNA origami dimers). A gel loading dye solution was added to the samples at a ratio of 1:5 before loading the samples in the gel pockets. The gel was run for 45 min at a constant voltage of 95 V using a BioRad Wide Mini-Sub Cell GT System and a BioRad PowerPac Basic power supply while keeping the gel electrophoresis chamber on an ice bath. For the gel at pH 8.2, the running buffer was 1× TAE buffer supplemented with 11 mM MgCl_2_, whereas 45 mM MES and 25 mM Tris supplemented with 11 mM MgCl_2_ was used as running buffer for the gel at pH 6.0. After the run, the gel was visualized by ultraviolet light using a BioRad Gel Doc XR+ documentation system.

### Transmission Electron Microscopy

The TEM samples were prepared on glow-charged (20 s oxygen plasma flash) Formvar carbon-coated copper grids (FCF400-Cu, Electron Microscopy Sciences) according to the protocol previously described by Castro *et al*.^54^ Three microliters of DNA origami solution (*c* = 5.0 nM for DNA origami units, *c* = 5.4/5.7 nM for DNA origami dimers and *c* = 2.0–5.0 nM for 1D DNA origami arrays) was applied onto the carbon-coated side of the grid and incubated for 3 min before excess sample solution was blotted away with filter paper. After that, the sample was negatively stained with 2% (w/v) aqueous uranyl formate solution containing 25 mM NaOH (added to increase the pH of the stain solution) in two subsequent steps. First, the sample was immersed into a 5 μL droplet of stain solution, after which the stain was immediately removed using filter paper. Next, the sample was immersed into a 20 μL droplet of stain solution for 45 s before the solution was blotted away with a filter paper. The samples were left to dry under ambient conditions for at least 15 min before imaging. All TEM images were obtained using a FEI Tecnai 12 Bio-Twin electron microscope operated at an acceleration voltage of 120 kV. The images were processed and analyzed (vertex angle measurements) using ImageJ.

### Atomic Force Microscopy

The atomic force microscopy (AFM) images were obtained using a Dimension Icon AFM (Bruker). The samples were imaged in air using ScanAsyst in Air Mode and ScanAsyst-Air probes (Bruker). The AFM images were recorded with a resolution of 512 pxl × 512 pxl and a scan rate of 0.5 or 0.75 Hz depending on the scan size (5 μm × 5 μm, 3 μm × 3 μm, or 2 μm × 2 μm). The images were processed (row alignment, correction of horizontal scars, and height scale adjustment) using NanoScope Analysis (v. 1.90, Bruker) and/or Gwyddion open source software (v. 2.58).^58^

## Supporting Information Available

Material sources and supplementary methods including molar extinction coefficients for different versions of the DNA origami unit, characterization of the different DNA origami units by agarose gel electrophoresis, TEM and AFM, characterization of the dimer formation by agarose gel electrophoresis and TEM, TEM and AFM images of the 1D array formation (both in solution and on mica), AFM images of 2D lattice formation on mica using different parameters (different pH values, MgCl_2_ concentrations, assembly times and unit types), AFM images demonstrating the pH-responsiveness of already assembled lattices, additional AFM images of AuNP lattices, caDNAno designs for the DNA origami units, and staple strand sequences for the DNA origami units (PDF)
