# Supplementary material for: Reconfigurable pH-Responsive DNA Origami Lattices
Source: ACS Nano. 2023 May 31;17(11):11014–22. doi: 10.1021/acsnano.3c03438 (PMC10278177; doi:10.1021/acsnano.3c03438)
Supplement: Supplementary file 1 — nn3c03438_si_002.pdf [file nn3c03438_si_002.pdf]

Supporting Information

Reconfigurable pH-Responsive DNA Origami Lattices

Sofia Julin[1], **Veikko Linko**[1,2,3*] **& Mauri A. Kostiainen**[1,2*]

[1]Biohybrid Materials, Department of Bioproducts and Biosystems, Aalto University, P.O. Box 16100, 00076 Aalto, Finland

[2]LIBER Center of Excellence, Aalto University, P.O. Box 16100, 00076 Aalto, Finland

[3]Institute of Technology, University of Tartu, Nooruse 1, 50411 Tartu, Estonia

[*]Correspondence and requests for materials should be addressed to veikko.pentti.linko@ut.ee or mauri.kostiainen@aalto.fi

Contents

Page

1 Materials **S3**

2 Supplementary Methods **S4**
2.1 DNA Origami Concentration Estimation S4
2.2 AFM Imaging of the DNA Origami Unit S4
2.3 pH-Responsiveness of Rehydrated 2D DNA Origami Lattices S4

3 pH-Responsive DNA Origami Unit **S6**

4 pH-Responsive DNA Origami Dimers **S14**

5 pH-Responsive 1D DNA Origami Arrays **S23**

6 pH-Responsive and Reconfigurable 2D DNA Origami Lattices **S28**

7 DNA-Templated, pH-Responsive, and Reconfigurable 2D AuNP Lattices **S46**

8 DNA Origami Unit Design **S49**

9 References **S58**

1. Materials

All chemicals were purchased from commercial suppliers and used as received unless otherwise noted. For preparation of the DNA origami unit, the circular single-stranded p7249 scaffold (c = 100 nM) was purchased from Tilibit Nanosystems and the single-stranded staple strands from Integrated DNA Technologies. 50× TAE buffer (2 M tris(hydroxymethyl)aminomethane (Tris), 1 M acetic acid, 50 mM ethylenediaminetetraacetic acid (EDTA), pH 8.4) was purchased from Thermo Fischer Scientific. For the agarose gel electrophoresis, the ethidium bromide and gel loading dye solution were purchased from Sigma Aldrich, whereas the agarose was purchased from Meridian Bioscience. The gel loading dye solution (0.25% bromophenol blue, 0.25% xylene cyanol, 40% sucrose) was diluted 1:30 in 40% (w/v) sucrose before used. The uranyl formate for staining the TEM samples were purchased from Electron Microscopy Sciences. The citrate stabilized gold nanoparticles (10 nm in diameter) were purchased from either Sigma Aldrich or nanoComposix. For all of the experiments, deionized water (Milli-Q grade) was used.

2. Supplementary Methods

2.1. DNA Origami Concentration Estimation

The DNA origami concentration after PEG purification was estimated from the DNA origami absorbance at 260 nm using Beer-Lambert law:

$$A_{260} = \epsilon_{260} c l \tag{S1}$$

where A_{260} is the absorbance at a wavelength of 260 nm, ϵ_{260} is the approximated molar extinction coefficient of the DNA origami structure at 260 nm and l is the path lenght through the solution in centimeters (0.05 cm in the used set-up). The absorbance at 260 nm was measured using a BioTek Eon Microplate Spectrophotometer and a Take3 microvolume plate. A sample size of 2 μL was used for the measurements and the concentration was obtained as the average of three measurements. The molar extinction extinction coefficients, ϵ_{260}, where calculated as

$$\epsilon_{260} = 6700 \times N_{\mathrm{ds}} + 10000 \times N_{\mathrm{ss}} \tag{S2}$$

where N_{ds} is the number of hybridized nucleotides and N_{ss} is the number of non-hybridized, single-stranded nucleotides in the DNA origami unit.(1)

Table S1. Molar extinction coefficients for different versions of the DNA origami unit.

**DNA origami unit**	**Extinction coefficient ($M^{-1}cm^{-1}$)**
DNA origami unit with pH latches, polyT-passivated	1.02×10^8
Permanently closed DNA origami unit, polyT-passivated	1.02×10^8
Permanently open DNA origami unit, polyT-passivated	1.01×10^8
DNA origami unit with pH latches for dimer formation	1.01×10^8
DNA origami unit with pH latches, polyT-passivated on the top arm (used for 1D arrays)	0.98×10^8
DNA origami unit with pH latches and scaffold loops (used for 2D lattices)	0.94×10^8
DNA origami unit with pH latches, AuNP attachment strands and scaffold loops (used for 2D lattices)	0.95×10^8
Permanently closed DNA origami unit with scaffold loops (used for 2D lattices)	0.94×10^8
Permanently closed DNA origami unit with AuNP attachment strands and scaffold loops (used for 2D lattices)	0.94×10^8

2.2. AFM Imaging of the DNA Origami Unit

10 μL of poly-T passivated DNA origami units with pH latches (PEG-purified, c = 1 nM for the sample at pH 8.2 and c = 2 nM for the sample at pH 6.0) was deposited onto a freshly cleaved mica surface (15 mm × 15 mm, grade V1, Electron Microscopy Sciences) and incubated covered at room temperature for 1 min. After the incubation, the mica surface was rinsed 3 times with 100 μL of deionized water, after which the sample was dried thorouhly using a nitrogen gas stream.

The atomic foce microscopy (AFM) images were obtained using a Dimension Icon AFM (Bruker). The samples were imaged in air using ScanAsyst in Air Mode and ScanAsyst-Air probes (Bruker). The AFM images were recorded with a resolution of 512 pxl × 512 pxl, a scan rate of 0.5 Hz and a scan size of 2 μm × 2 μm. The images were processed using Gwyddion open source software (v. 2.58). (2)

2.3. pH-Responsiveness of Rehydrated 2D DNA Origami Lattices

In order to demonstrate that assembled and dried 2D lattices still are pH-responsive and reconfigurable if rehydrated, the following approach was used. PEG-purified DNA origami units (final concentration of 2.0 nM) were mixed with 10-fold excess of connector oligonucleotides in 1× TAE supplemented with 12.5 mM $MgCl_2$ and 75 mM NaCl at pH 6.0 (lattice assembled at pH 6.0) or in 1× TAE supplemented with 10 mM $MgCl_2$ and 75 mM NaCl at pH 8.2 (lattice assembled at pH 8.2). 120 μL of the DNA origami sample mixture was evenly deposited onto a freshly cleaved mica surface (15 mm × 15 mm, grade V1, Electron Microscopy Sciences) and incubated covered at room temperature for 3 or 24 h. After the incubation, the mica surface was rinsed 3 times with 100 μL of deionized water, after which the sample was dried thoroughly using a nitrogen gas stream. Note that, the rehydrated lattice will not be pH-responsive and reconfigurable if attached to the mica substrate with $NiCl_2$ before the drying (see **Figure S36**).

After drying and AFM imaging of the sample, the lattice was rehydrated and at the same time the pH increased/decreased. In order to rehydrate (and increase the pH of) the lattice assembled at pH 6.0, 120 μL of 1× TAE, 20 mM $MgCl_2$ and 75 mM

NaCl at pH 8.2 was deposited on the lattice and incubated covered for 2 hours. Similarly, in order to rehydrate (and decrease the pH of) the lattice assembled at pH 8.2, 120 μL of 1× TAE, 20 mM $MgCl_2$ and 75 mM NaCl at pH 6.0 was deposited on the lattice and incubated covered for 20 hours. After the incubation, the mica surface was rinsed 3 times with 100 μL of 1× TAE, 20 mM $MgCl_2$ at pH 8.2 (for the lattice changed to pH 8.2) or 100 μL of 1× TAE, 20 mM $MgCl_2$ at pH 6.0 (for the lattice changed to pH 6.0). Immediately after this washing step, 120 μL of 1× TAE, 10 mM $NiCl_2$ was deposited on the mica surface and incubated covered for 1 h. After the incubation, the mica surface was rinsed 6 times with 100 μL of deionized water, after which the sample was dried thoroughly using a nitrogen gas stream.

The atomic foce microscopy (AFM) images were obtained using a Dimension Icon AFM (Bruker). The samples were imaged in air using ScanAsyst in Air Mode and ScanAsyst-Air probes (Bruker). The AFM images were recorded with a resolution of 512 pxl × 512 pxl, a scan rate of 0.75 Hz and a scan size of 3 μm × 3 μm. The images were processed using Gwyddion open source software (v. 2.58). (2)

3. pH-Responsive DNA Origami Unit

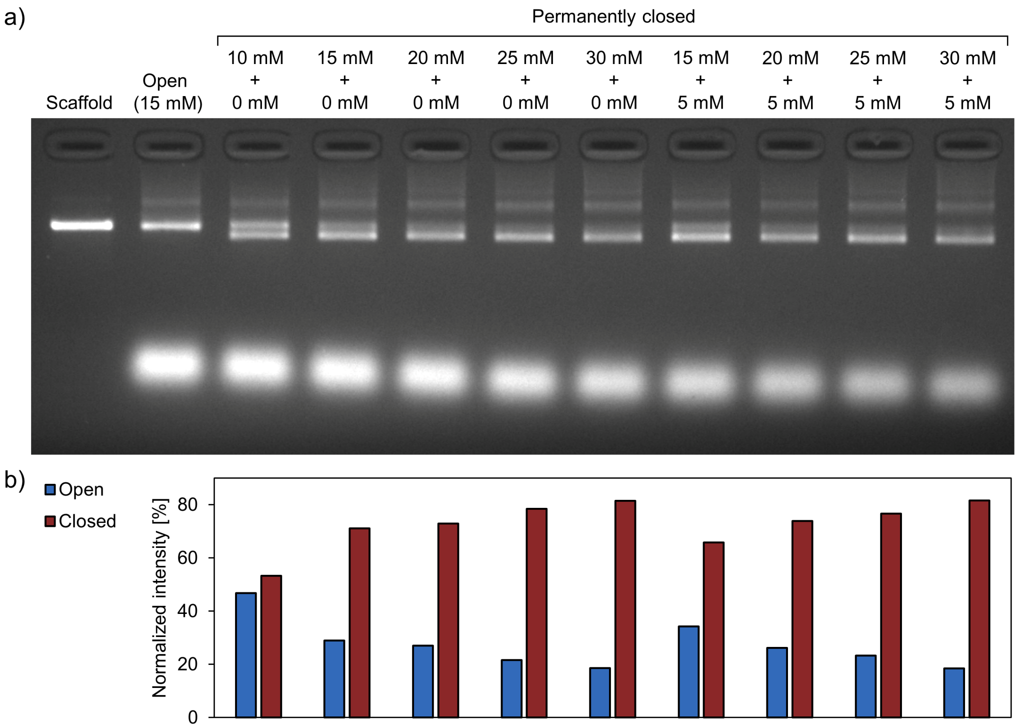

Figure S1. Characterization of permanently closed DNA origami units folded at different $MgCl_2$ and NaCl concentrations. (a) Agarose gel electrophoresis of folded, unpurified structures. The top concentration is the $MgCl_2$ concentration and the bottom the NaCl concentration. The DNA origami concentration in the gel is 11.1 nM and the gel was run at pH 8.2. (b) Ethidium bromide intensity of the bands corresponding to the closed and open configurations. The total intensity of the two bands corresponding to the open and the closed configuration was always set to 100 %.

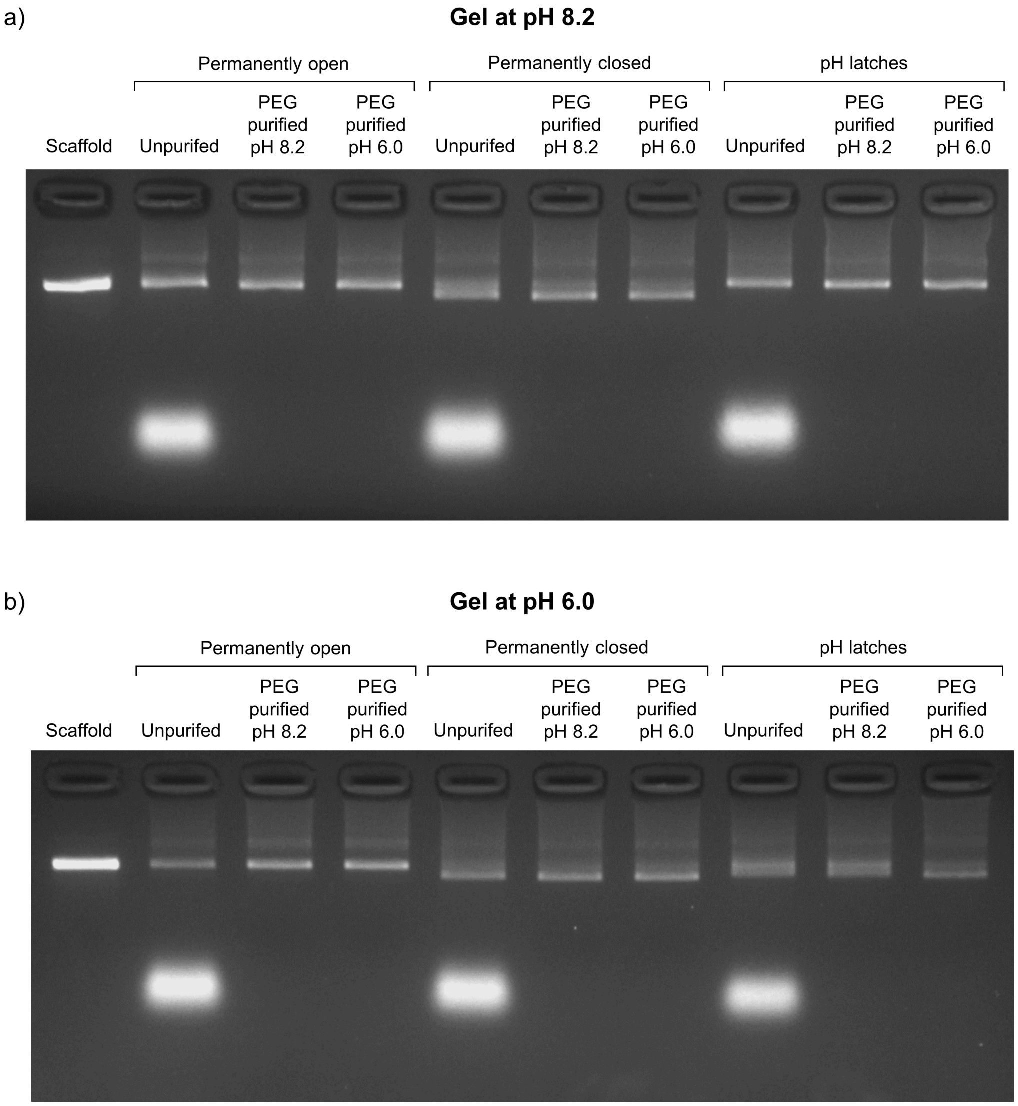

Figure S2. Characterization of the DNA origami units by agarose gel electrophoresis at (a) pH 8.2 and (b) pH 6.0. Selected parts of the gels are also shown in the article in Figure 1b. The DNA origami concentration in the gels is 15.0 nM.

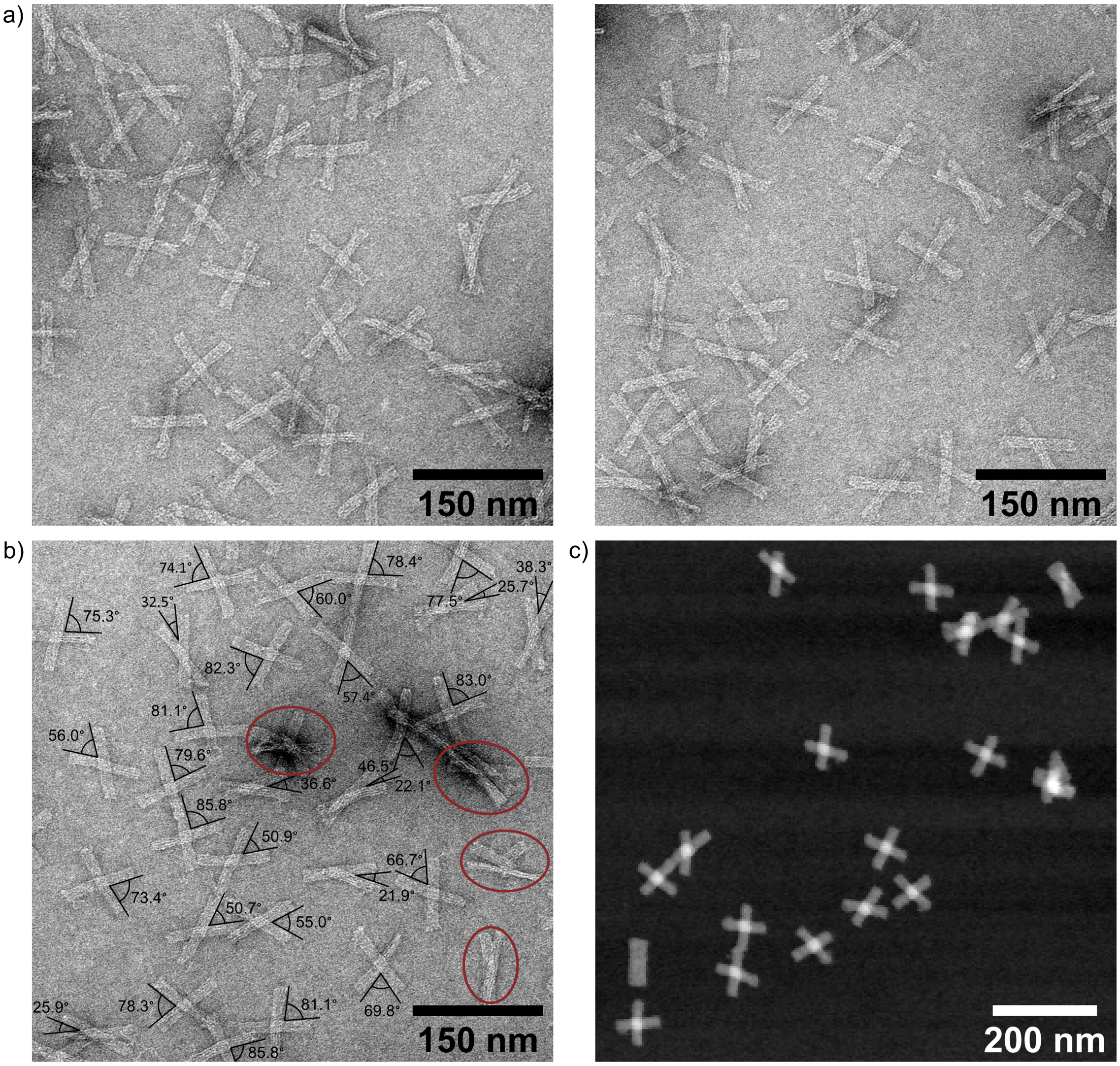

Figure S3. Characterization of the DNA origami unit with pH latches (poly-T passivated) at pH 8.2. (a) TEM images of the unit (c = 5.0 nM) in 1× FOB (1× TAE, 20 mM $MgCl_2$, 5 mM NaCl) at pH 8.2. (b) Examples of determined angles, α, between the two arms of the unit. The encircled structures were excluded from the analysis. (c) AFM image of the unit (c = 1 nM) in 1× FOB at pH 8.2. The TEM images are negatively stained with uranyl formate (2 % (w/v)).

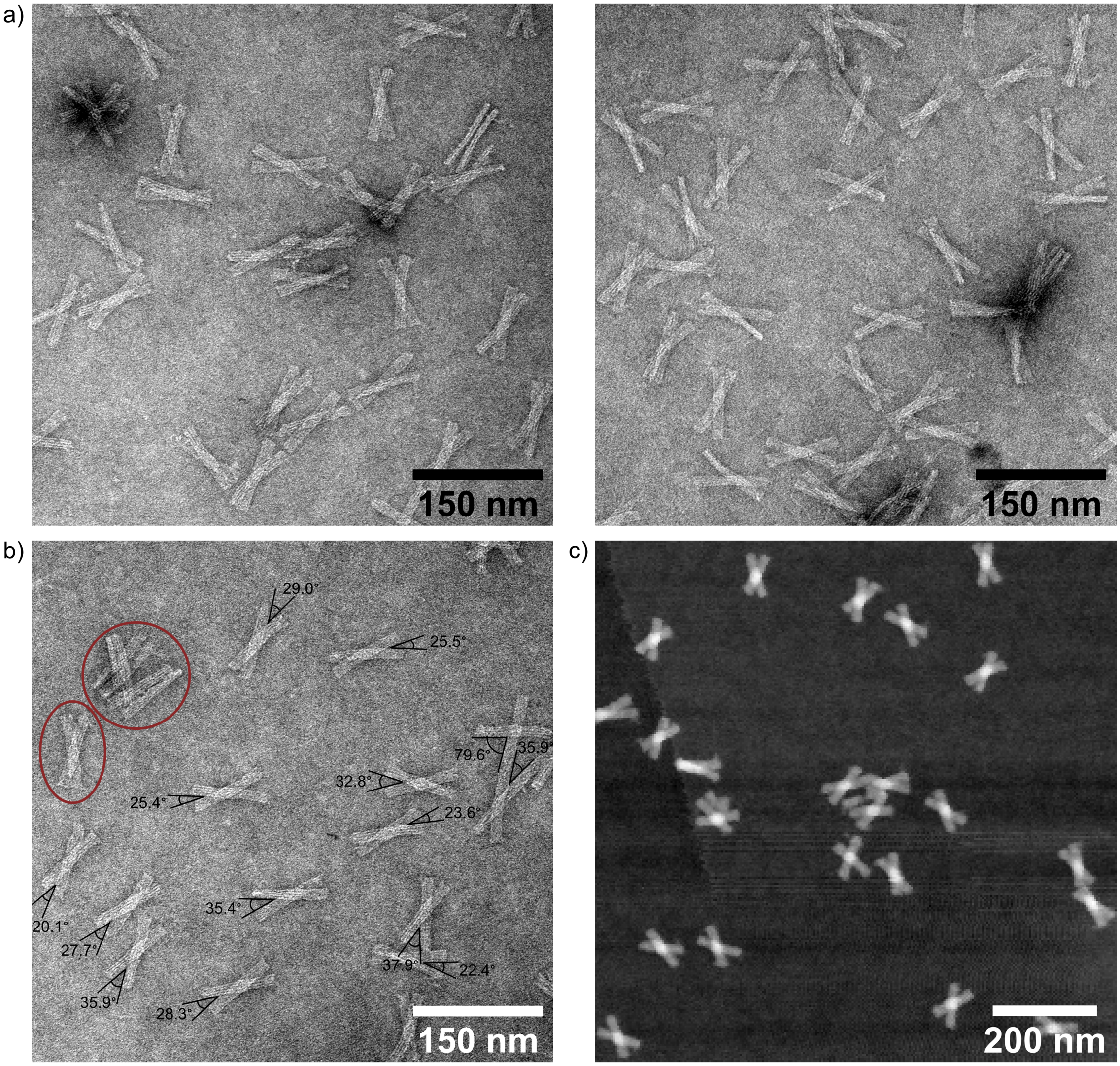

Figure S4. Characterization of the DNA origami unit with pH latches (poly-T passivated) at pH 6.0. (a) TEM images of the unit (c = 5.0 nM) in 1× FOB (1× TAE, 20 mM $MgCl_2$, 5 mM NaCl) at pH 6.0. (b) Examples of determined angles, α, between the two arms of the unit. The encircled structures were excluded from the analysis. (c) AFM image of the unit (c = 2 nM) in 1× FOB at pH 6.0. The TEM images are negatively stained with uranyl formate (2 % (w/v)).

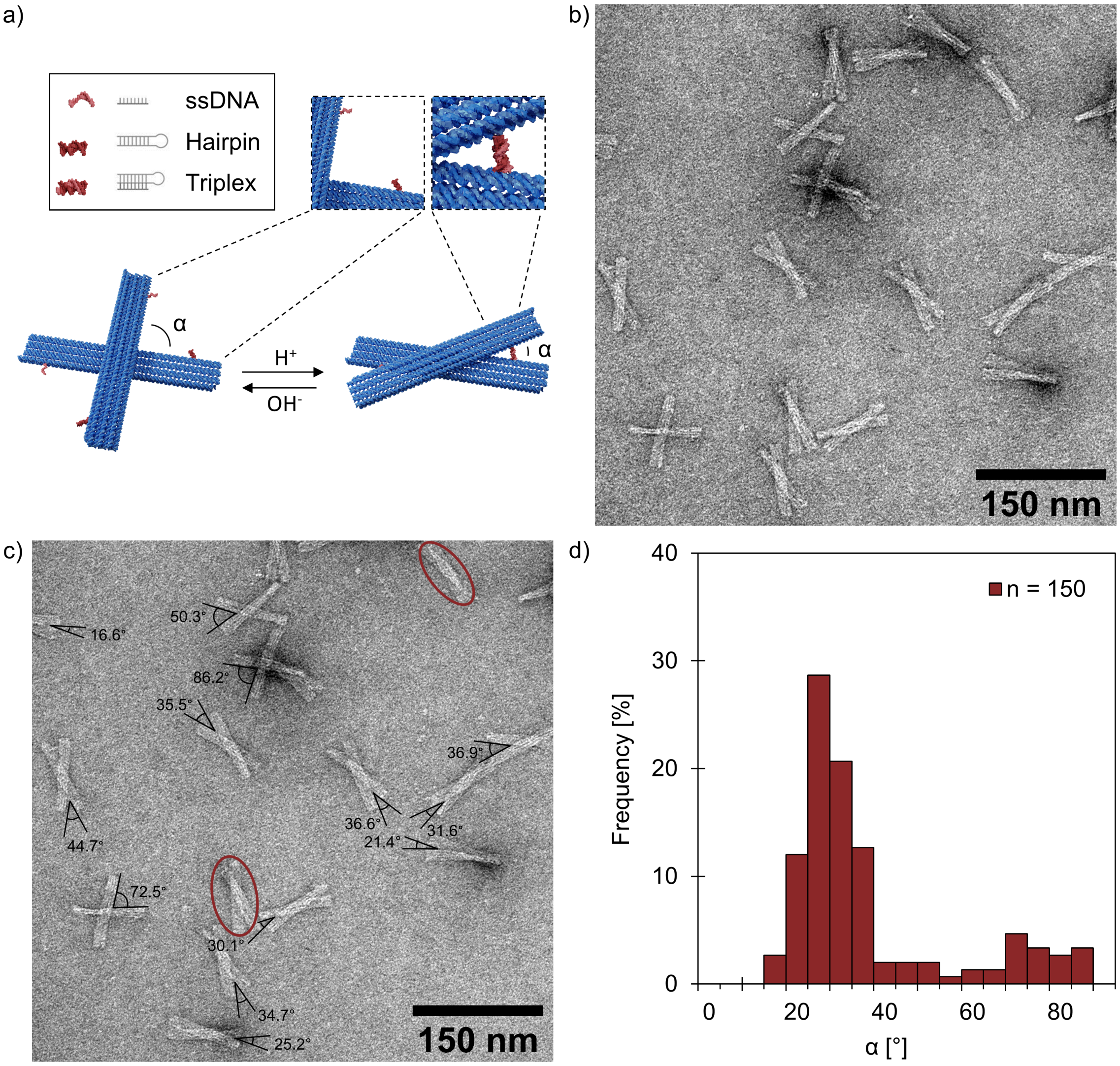

Figure S5. Characterization of the DNA origami unit with different pH latch configuration (poly-T passivated) at pH 6.0. (a) The pH latches are at different positions in the unit and therefore the arms of the unit will be locked in a reverse direction (clockwise instead of counterclockwise). (b) TEM images of the unit (c = 5.0 nM) in 1× FOB (1× TAE, 20 mM $MgCl_2$, 5 mM NaCl) at pH 6.0. (c) Examples of determined angles, α, between the two arms of the unit. The encircled structure was excluded from the analysis. (d) The distribution of the angle, α, between the two arms of the unit. The number of individual structures analyzed for the sample is n = 150. The TEM images are negatively stained with uranyl formate (2 % (w/v)).

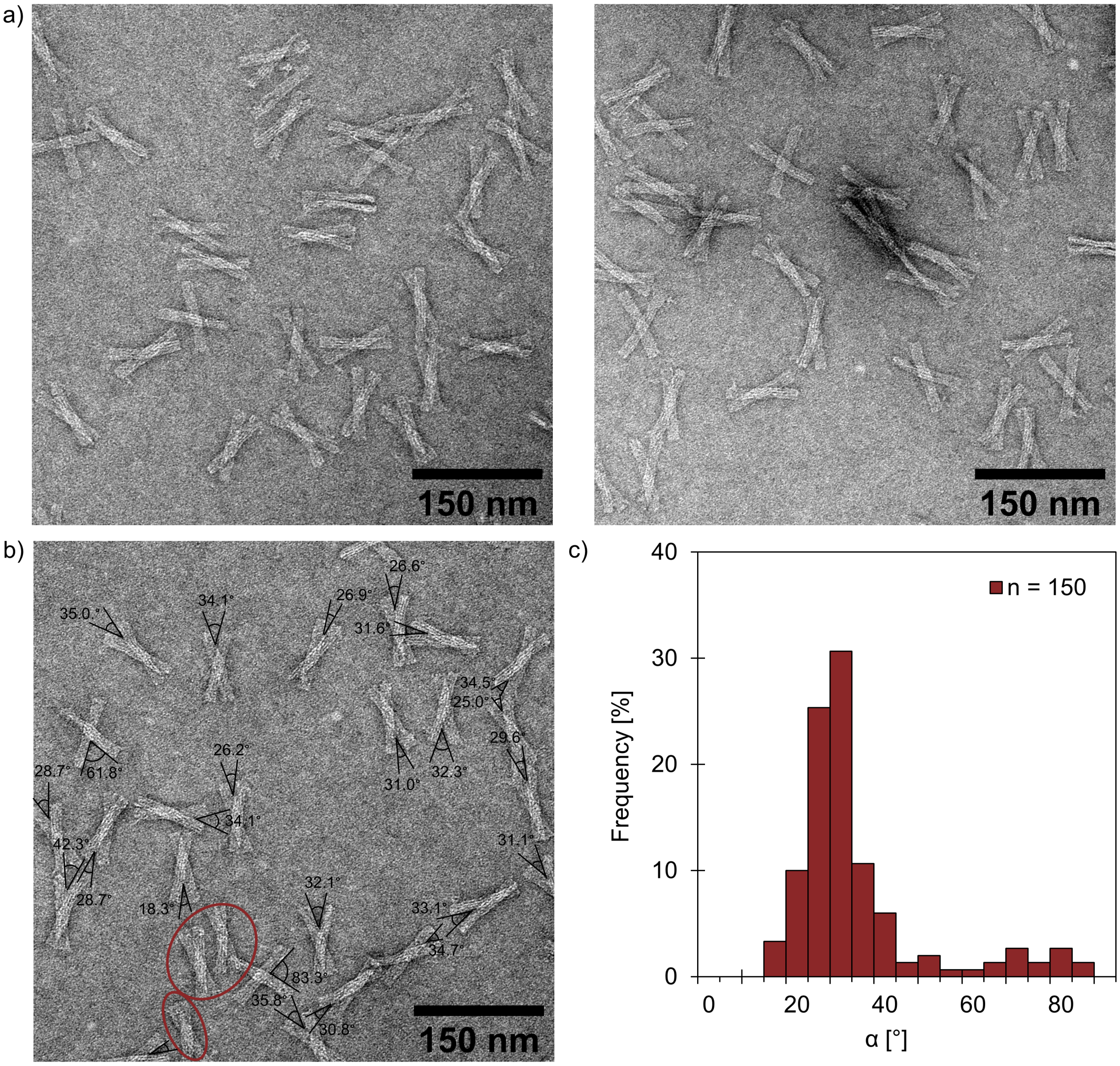

Figure S6. Characterization of the permanently closed DNA origami unit (poly-T passivated) at pH 8.2. (a) TEM images of the unit (c = 5.0 nM) in 1× FOB (1× TAE, 20 mM $MgCl_2$, 5 mM NaCl) at pH 8.2. (b) Examples of determined angles, α, between the two arms of the unit. The encircled structures were excluded from the analysis. (c) The distribution of the angle, α, between the two arms of the unit. The number of individual structures analyzed for the sample is n = 150. The TEM images are negatively stained with uranyl formate (2 % (w/v)).

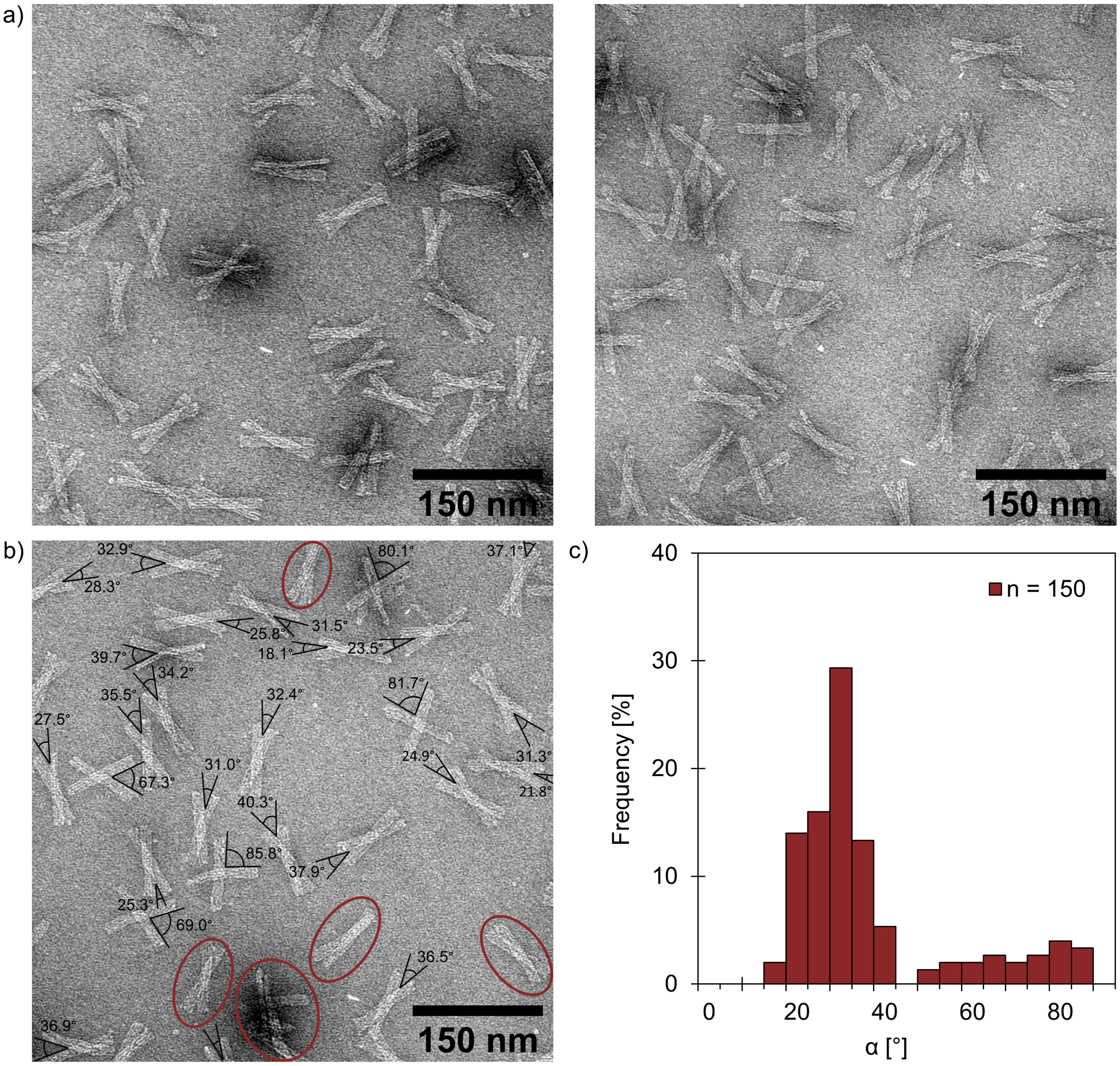

Figure S7. Characterization of the permanently closed DNA origami unit (poly-T passivated) at pH 6.0. (a) TEM images of the unit (c = 5.0 nM) in 1× FOB (1× TAE, 20 mM $MgCl_2$, 5 mM NaCl) at pH 6.0. (b) Examples of determined angles, α, between the two arms of the unit. The encircled structures were excluded from the analysis. (c) The distribution of the angle, α, between the two arms of the unit. The number of individual structures analyzed for the sample is n = 150. The TEM images are negatively stained with uranyl formate (2 % (w/v)).

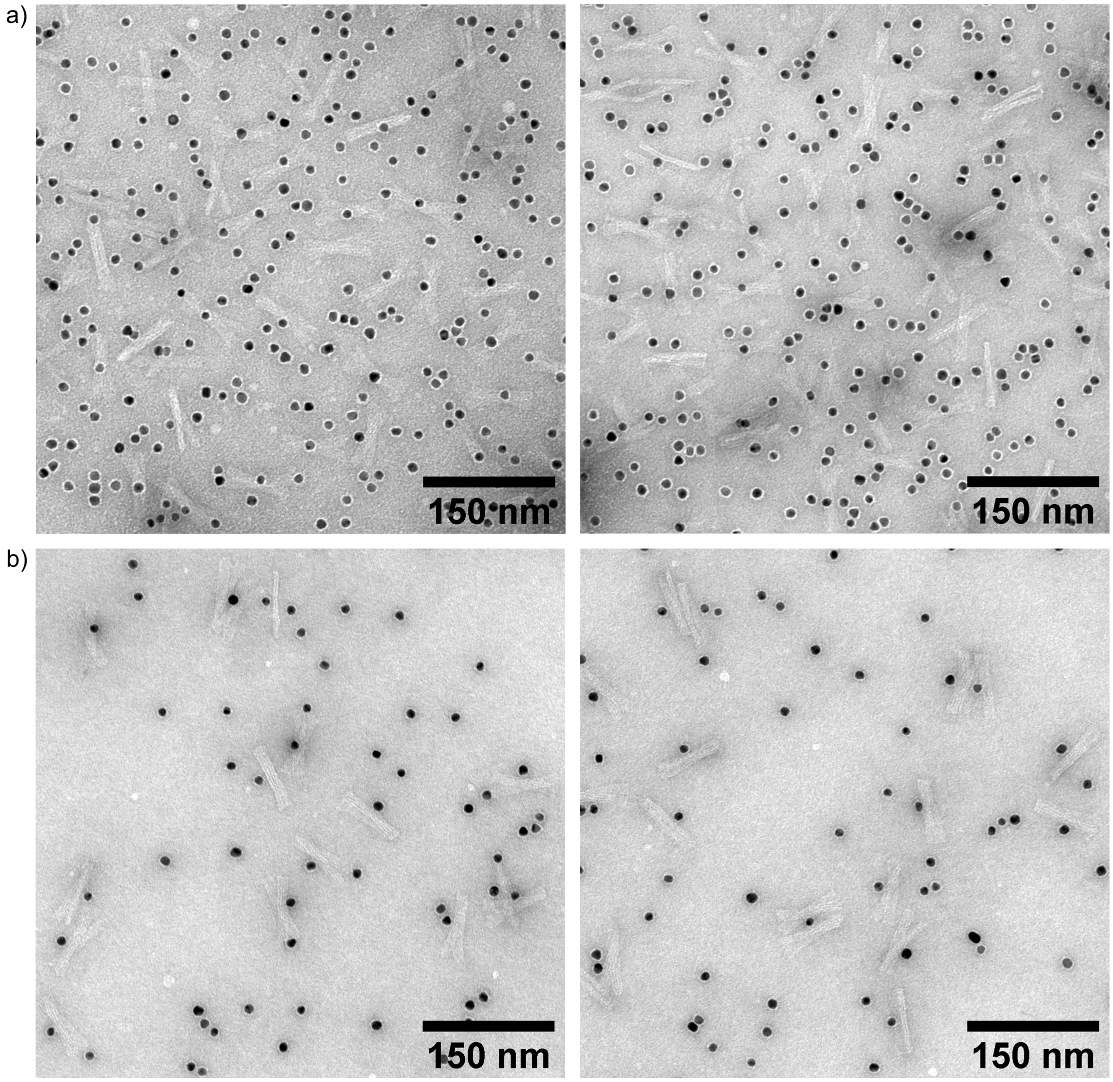

Figure S8. Characterization of the permanently closed DNA origami unit with scaffold loops and an anchored AuNP in the middle. TEM images of (a) unpurified units (c = 7.6 nM). (b) units that have been PEG-purified twice (c = 5.4 nM before the PEG purifications). The units are in 1× FOB (1× TAE, 20 mM $MgCl_2$, 5 mM NaCl) at pH 8.2 and the TEM images are negatively stained with uranyl formate (2 % (w/v)).

4. pH-Responsive DNA Origami Dimers

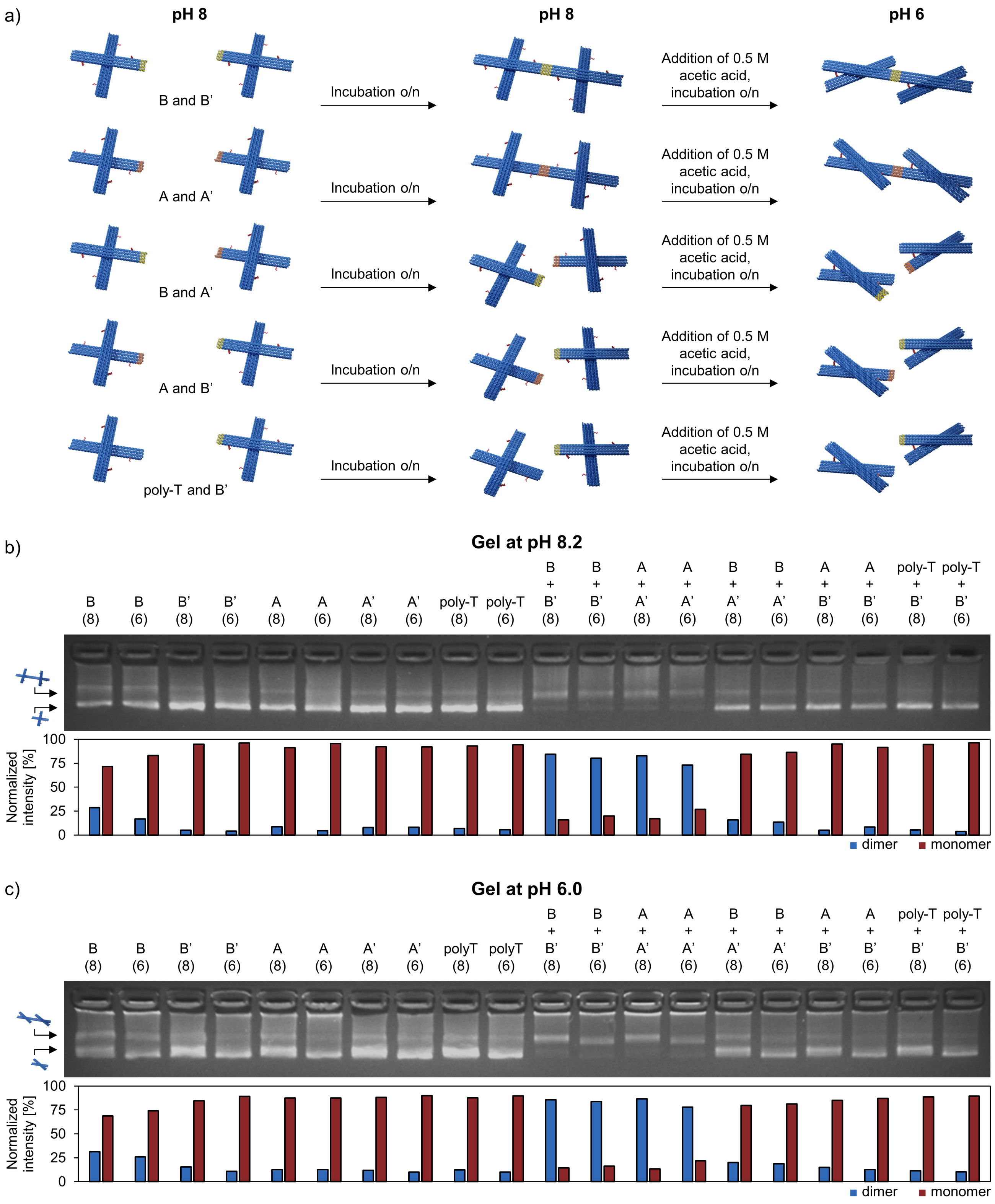

Figure S9. Characterization of the dimer formation at pH 8.2 by agarose gel electrophoresis. (a) Conceptual illustration of the dimerization when different DNA origami units are used. Dimers are selectively formed only for units with shape complementarity and appropriate connector oligonucleotides (mixture of B and B' units or mixture of A and A' units). The dimers formed at pH 8.2 are analyzed by agarose gel electrophoresis at (b) pH 8.2 and (c) pH 6.0 both before (samples marked '8') and after the pH has been decreased to pH 6 by 0.5 M acetic acid (samples marked '6'). The DNA origami concentrations in the gel are 15.0 nM for individual units and 5.4 nM (pH 6) or 5.9 nM (pH 8) in unit mixtures. Ethidium bromide intensity of the monomer and the dimer bands are shown below respective gel image. The total intensity of the monomer and the dimer band was always set to 100 %. Selected parts of the gels are also shown in the article in Figure 2b.

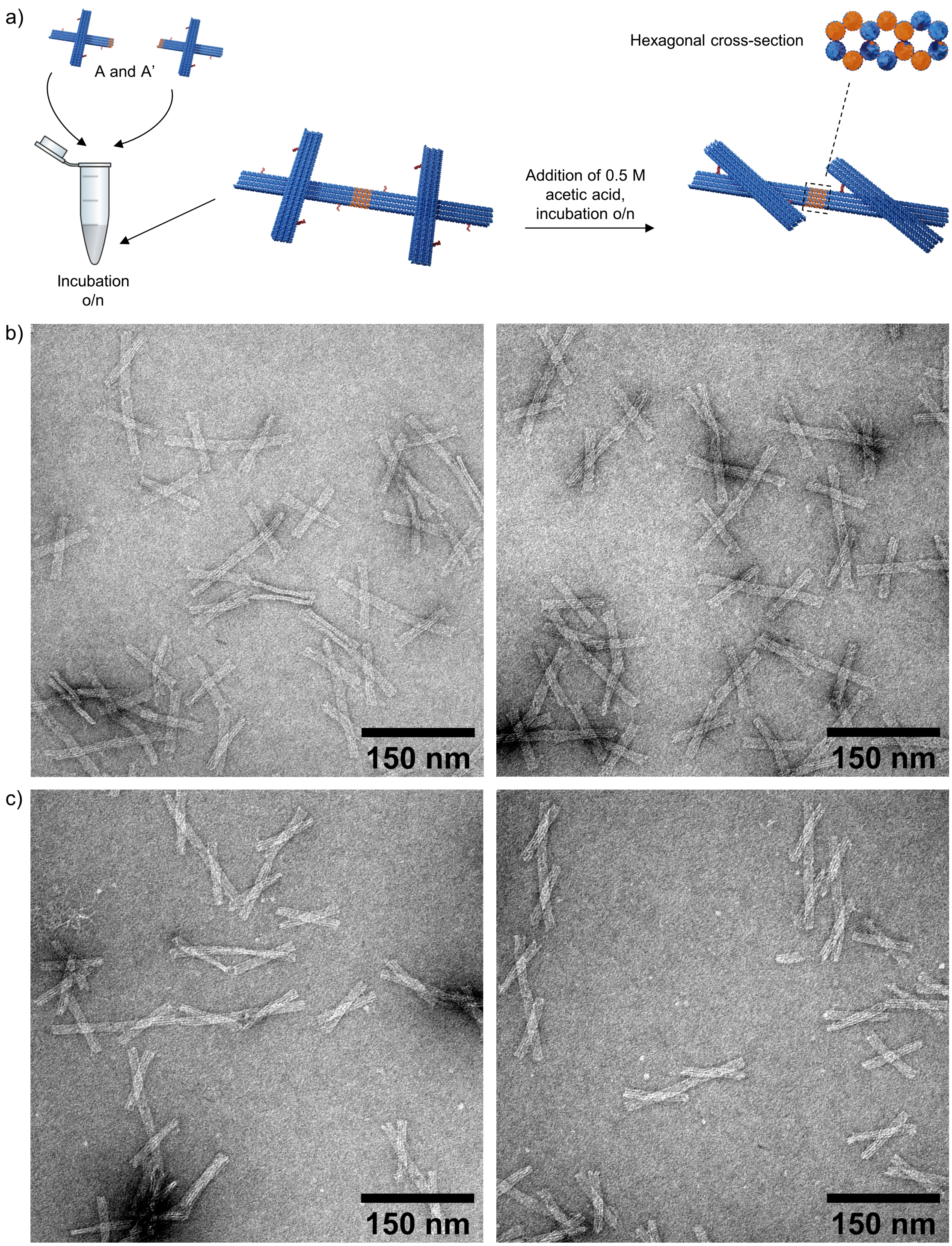

Figure S10. Dimer formation at pH 8.2 using A and A' DNA origami units. (a) Dimers are formed by mixing equimolar amounts of both units (A and A'). By adding acetic acid to the dimer solution, the arms could be locked into the closed configuration also after the dimerization. (b) TEM images of the dimers formed at pH 8.2 (c_{dimer} = 5.7 nM). (c) TEM images of the same dimer solution as in b) after the pH has been decreased to 6 (c_{dimer} = 5.4 nM). The TEM images are negatively stained with 2% (w/v) uranyl formate.

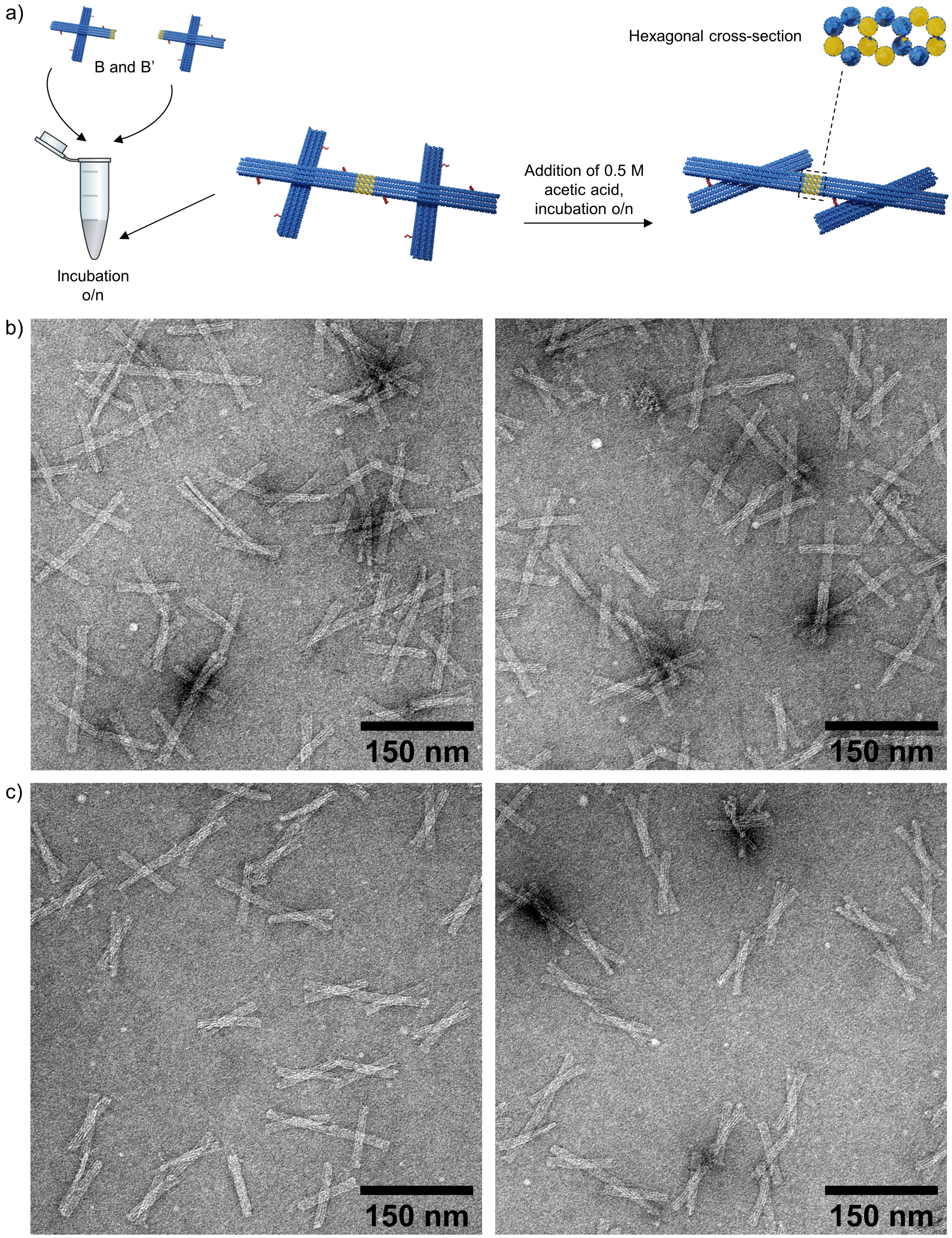

Figure S11. Dimer formation at pH 8.2 using B and B' DNA origami units. (a) Dimers are formed by mixing equimolar amounts of both units (B and B'). By adding acetic acid to the dimer solution, the arms could be locked into the closed configuration also after the dimerization. (b) TEM images of the dimers formed at pH 8.2 (c_{dimer} = 5.7 nM). (c) TEM images of the same dimer solution as in b) after the pH has been decreased to 6 (c_{dimer} = 5.4 nM). The TEM images are negatively stained with 2% (w/v) uranyl formate.

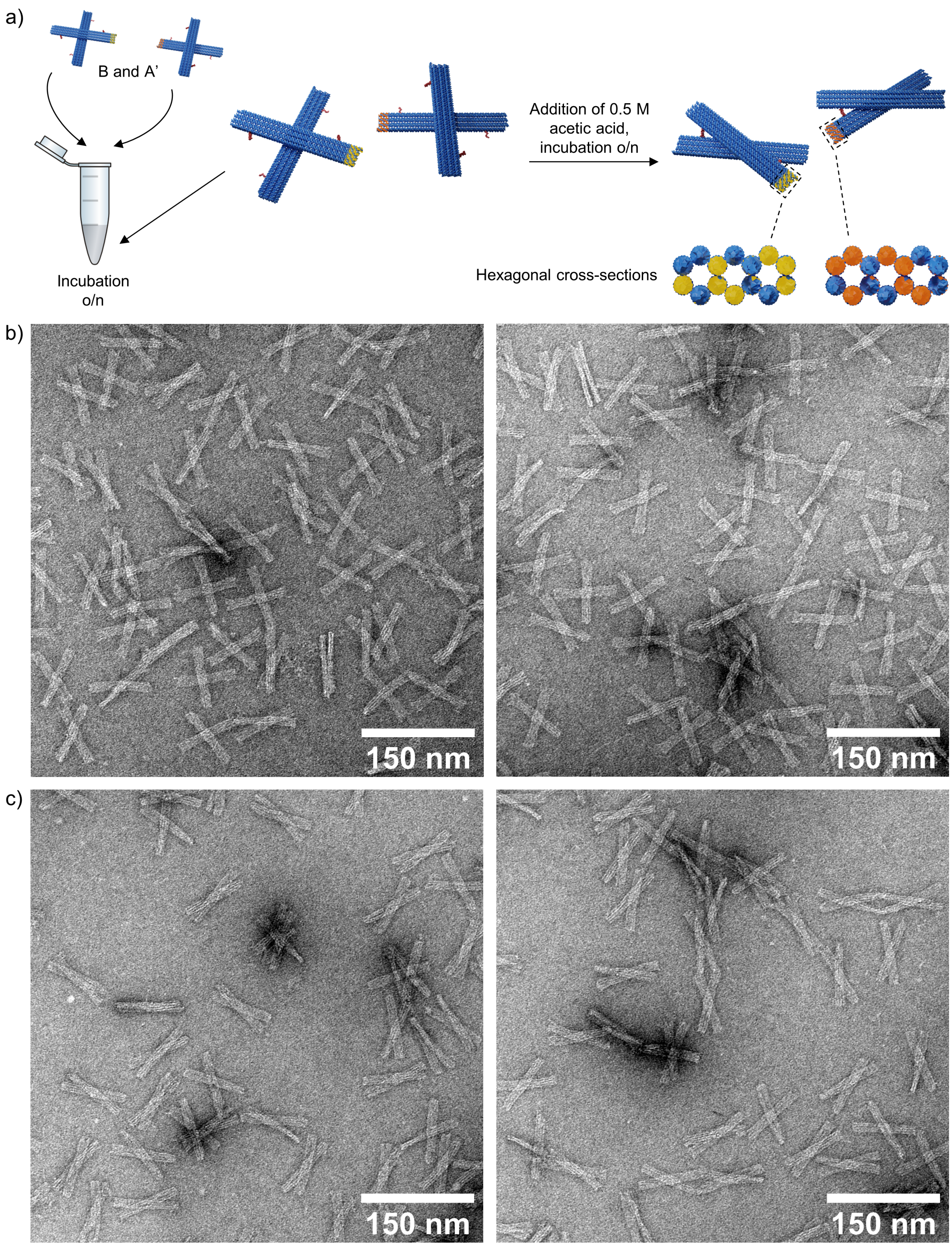

Figure S12. Mixture of B and A' DNA origami units at pH 8.2. (a) No dimers are formed by mixing equimolar amounts of units B and A'. By adding acetic acid to the DNA origami mixture, the units will adopt the closed configuration. (b) TEM images of the DNA origami mixture at pH 8.2 (c_{unit} = 5.7 nM). (c) TEM images of the same DNA origami mixture as in b) after the pH has been decreased to 6 (c_{unit} = 5.4 nM). The TEM images are negatively stained with 2% (w/v) uranyl formate.

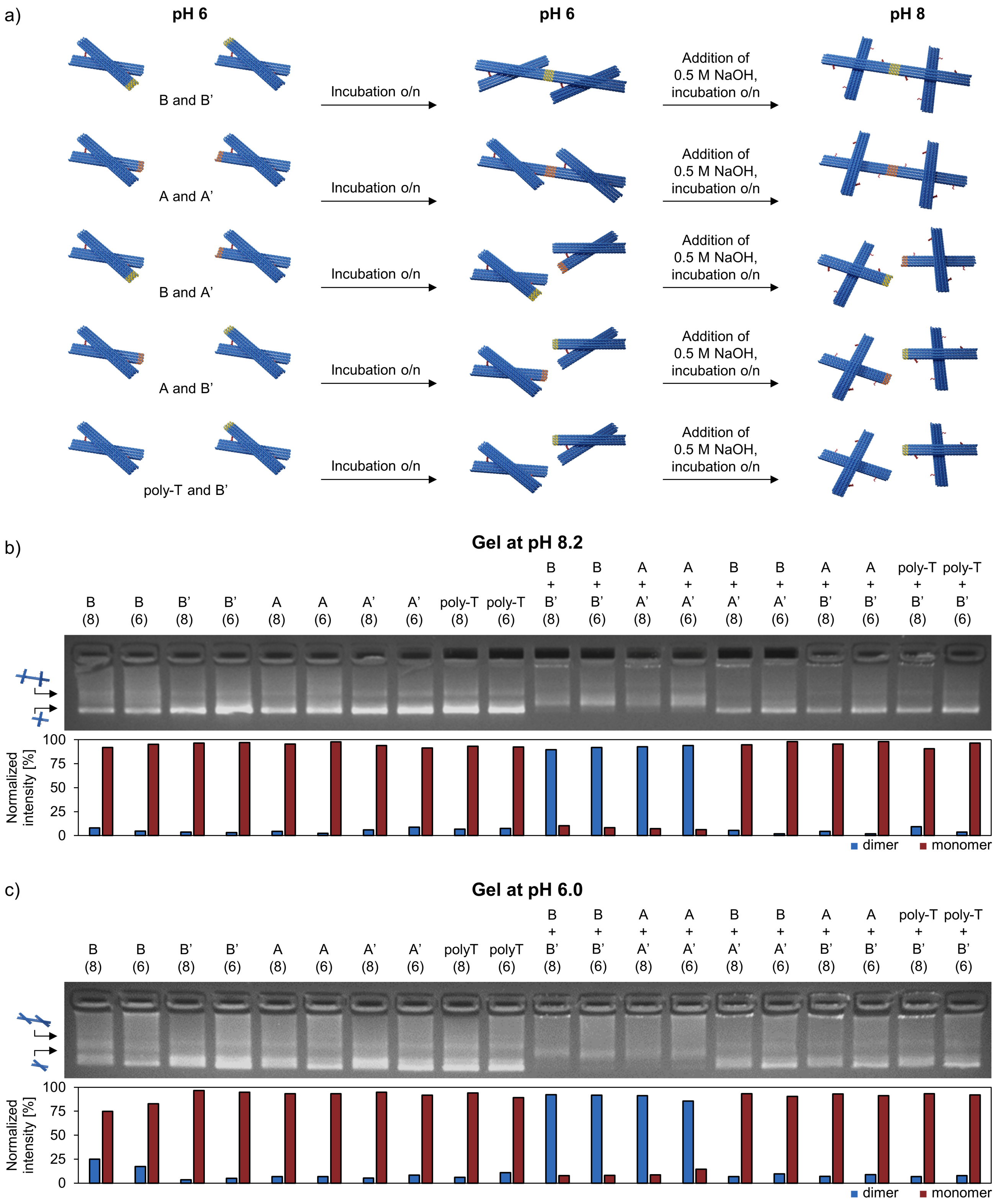

Figure S13. Characterization of the dimer formation at pH 6.0 by agarose gel electrophoresis. (a) Conceptual illustration of the dimerization when different DNA origami units are used. Dimers are selectively formed only for units with shape complementarity and appropriate connector oligonucleotides (mixture of B and B' units or mixture of A and A' units). The dimers formed at pH 6.0 are analyzed by agarose gel electrophoresis at (b) pH 8.2 and (c) pH 6.0 both before (samples marked '6') and after the pH has been increased to pH 8 by 0.5 M NaOH (samples marked '8'). The DNA origami concentrations in the gel are 15.0 nM for individual units and 5.4 nM (pH 8) or 5.9 nM (pH 6) in unit mixtures. Ethidium bromide intensity of the monomer and the dimer bands are shown below respective gel image. The total intensity of the monomer and the dimer band was always set to 100 %.

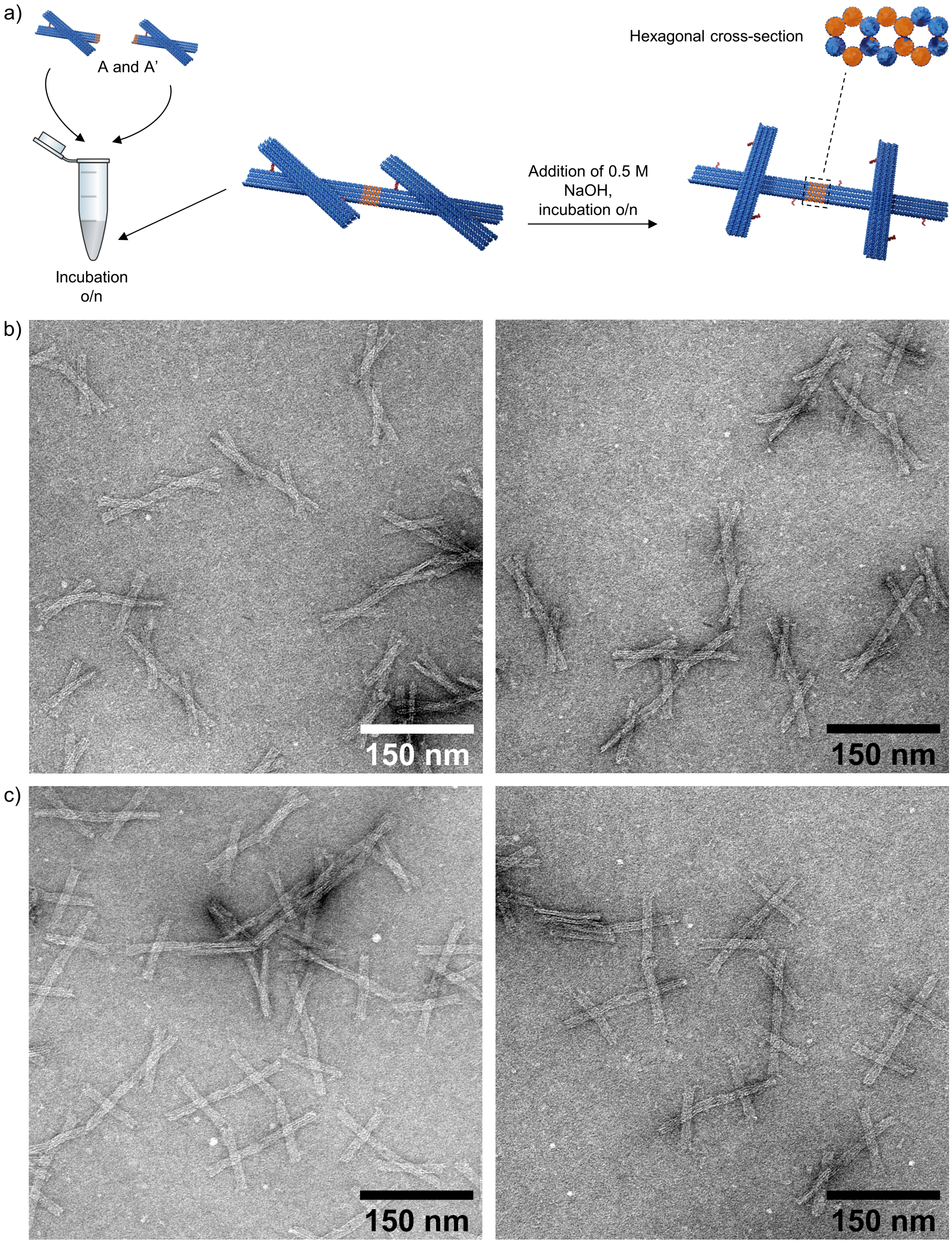

Figure S14. Dimer formation at pH 6.0 using A and A' DNA origami units. (a) Dimers are formed by mixing equimolar amounts of both units (A and A'). By adding NaOH to the dimer solution, the arms could be released again also after the dimerization. (b) TEM images of the dimers formed at pH 6.0 (c_{dimer} = 5.7 nM). (c) TEM images of the same dimer solution as in b) after the pH has been increased to 8 (c_{dimer} = 5.4 nM). The TEM images are negatively stained with 2% (w/v) uranyl formate.

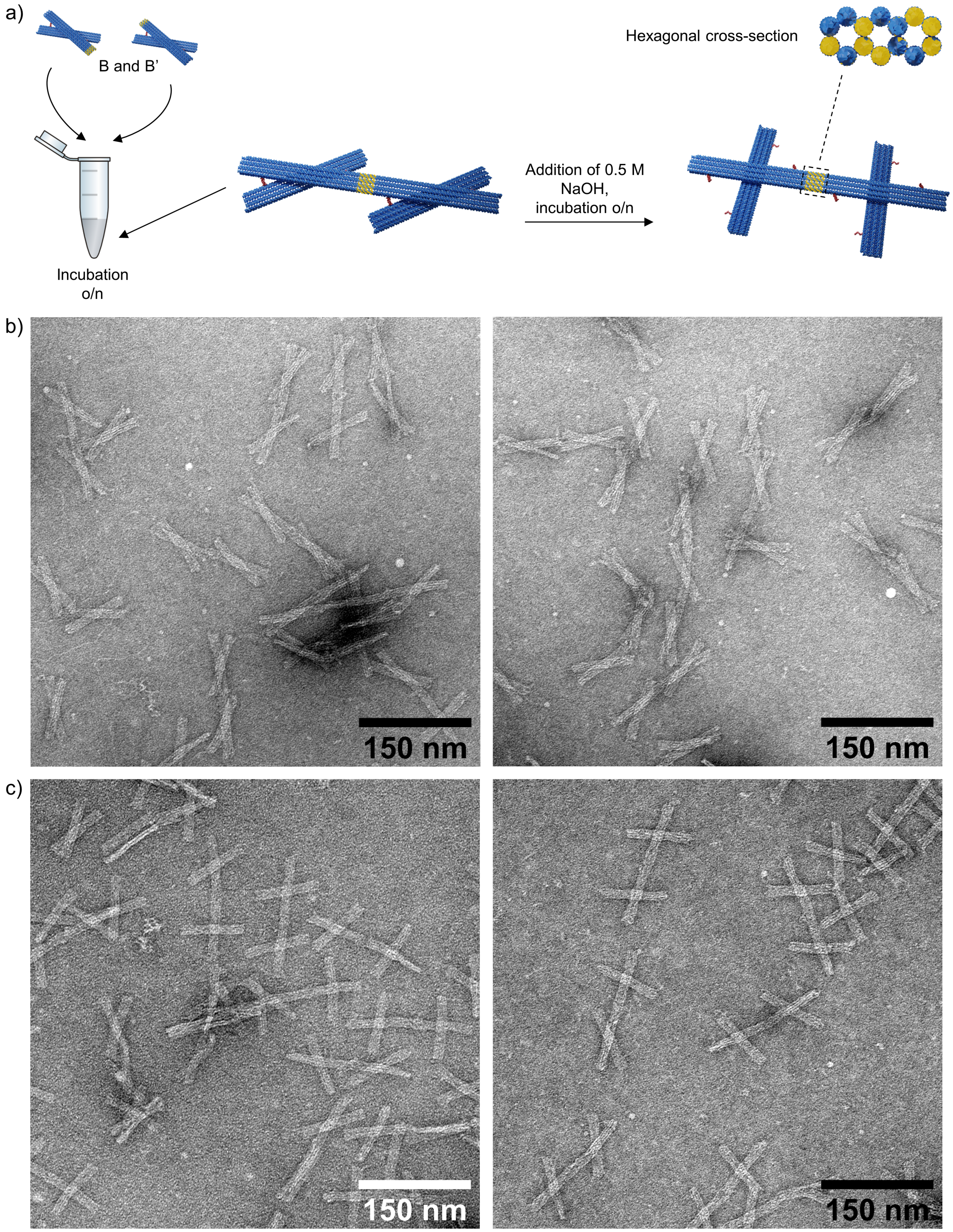

Figure S15. Dimer formation at pH 6.0 using B and B' DNA origami units. (a) Dimers are formed by mixing equimolar amounts of both units (B and B'). By adding NaOH to the dimer solution, the arms could be released again also after the dimerization. (b) TEM images of dimers formed at pH 6.0 (c_{dimer} = 5.7 nM). (c) TEM images of the same dimer solution as in b) after the pH has been increased to 8 (c_{dimer} = 5.4 nM). The TEM images are negatively stained with 2% (w/v) uranyl formate.

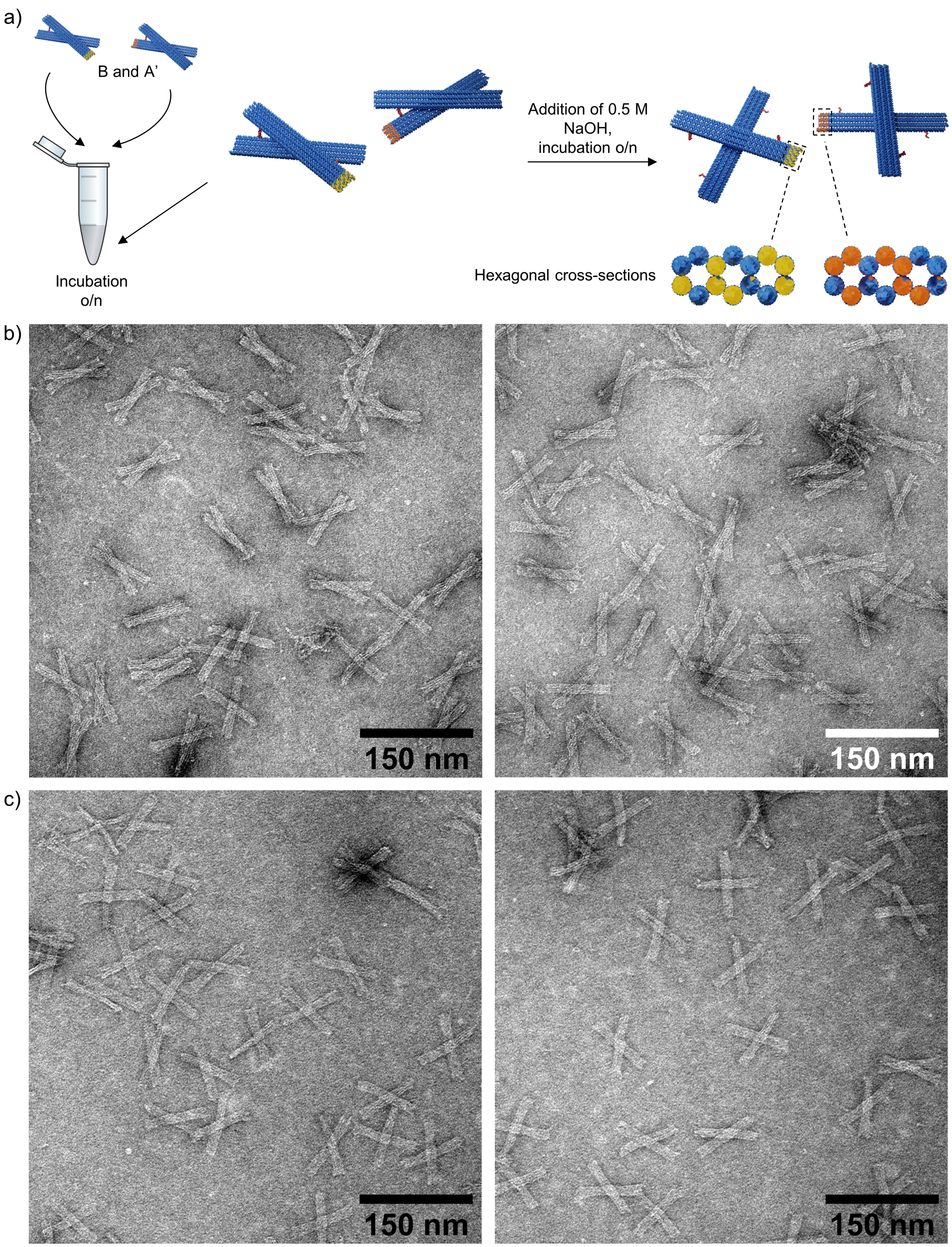

Figure S16. Mixture of B and A' DNA origami units at pH 6.0 (a) No dimers are formed by mixing equimolar amounts of units B and A'. By adding NaOH to the DNA origami mixture, the units will adopt the open configuration. (b) TEM images of the DNA origami mixture at pH 6.0 (c_{unit} = 5.7 nM). (c) TEM images of the same DNA origami mixture as in b) after the pH has been increased to 6 (c_{unit} = 5.4 nM). The TEM images are negatively stained with 2% (w/v) uranyl formate.

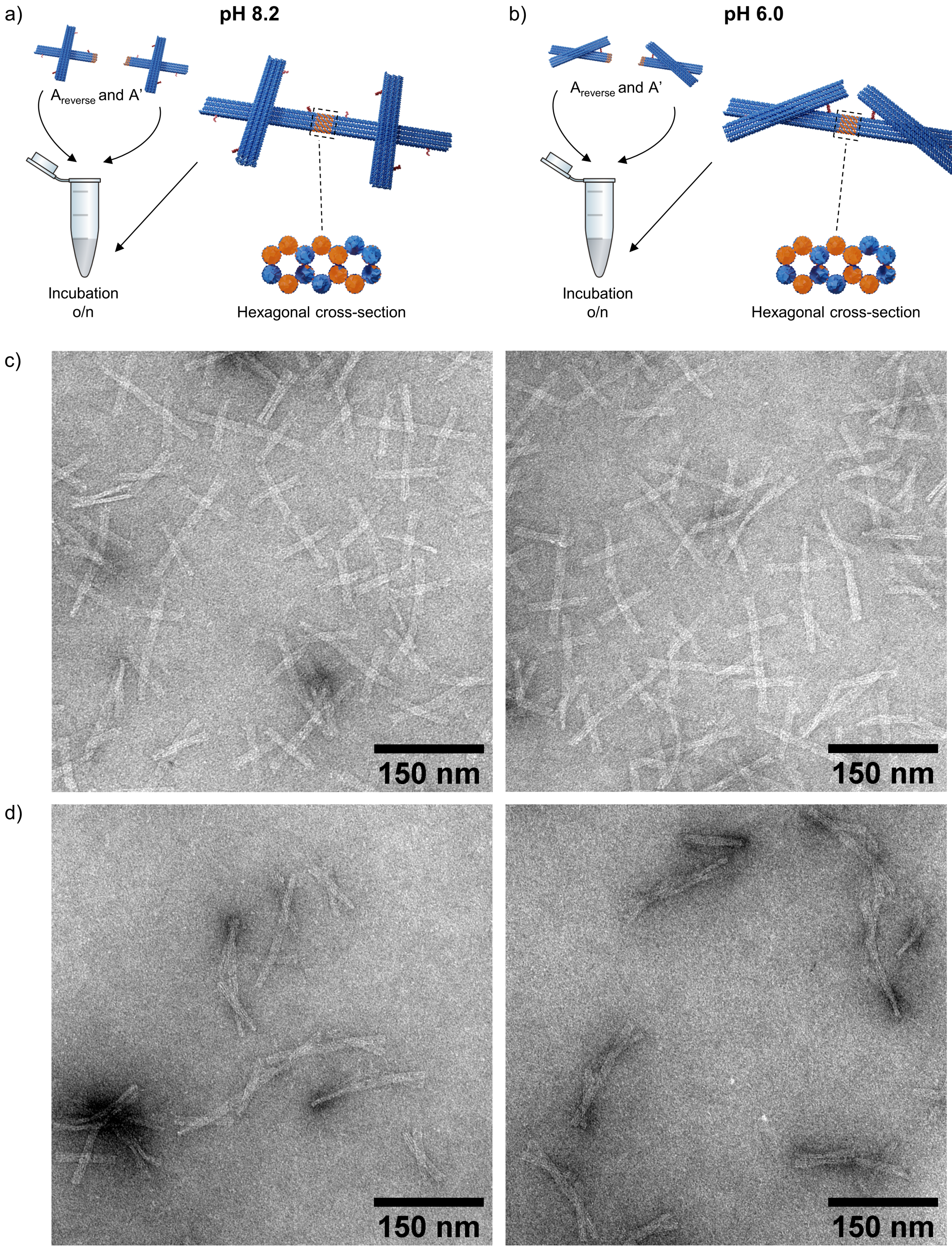

Figure S17. Dimer formation using units with different types of pH locks. Dimers are formed using equimolar amounts of units $A_{reverse}$ and A’. The $A_{reverse}$ unit has the reversed pH latches, whereas the A’ unit have the regular pH latches. The dimers assembled at (a) pH 8.2 have their arms in an open configuration, whereas the dimers assembled at (b) pH 6.0 have arms that point towards each other. TEM images of the dimers formed at (c) pH 8.2 (c_{dimer} = 5.7 nM) and (d) pH 6.0 (c_{dimer} = 5.7 nM). The TEM images are negatively stained with 2% (w/v) uranyl formate.

5. pH-Responsive 1D DNA Origami Arrays

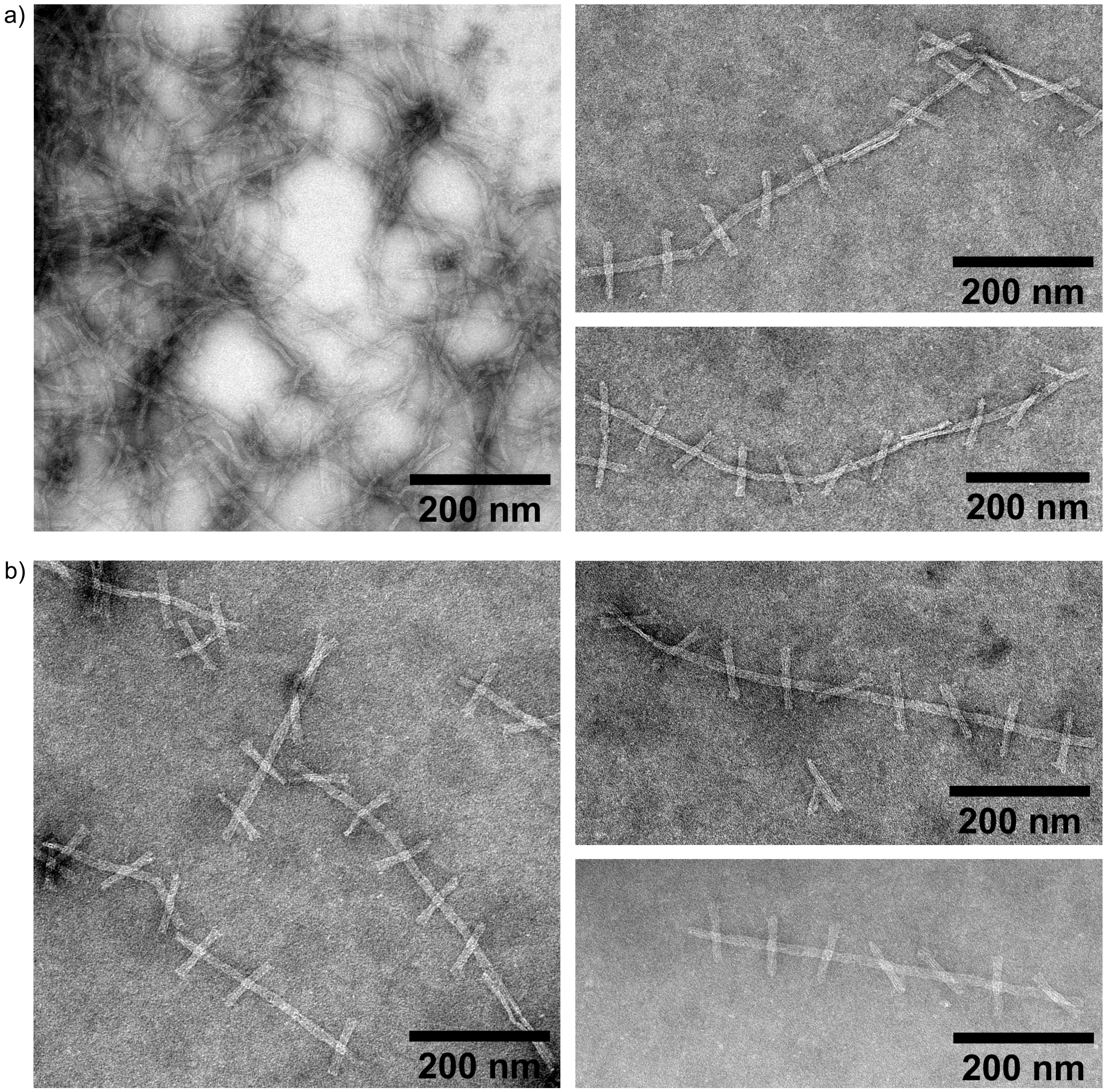

Figure S18. TEM images of linear 1D arrays formed in solution at pH 8.2 (25 h incubation at room temperature). The 1D chains were assembled using DNA origami unit concentrations of (a) 10.0 nM (diluted 1:2 in 1× FOB (1× TAE, 20 mM $MgCl_2$, 5 mM NaCl) before deposited onto the TEM grid) (b) 5.0 nM. The TEM images are negatively stained with 2% (w/v) uranyl formate.

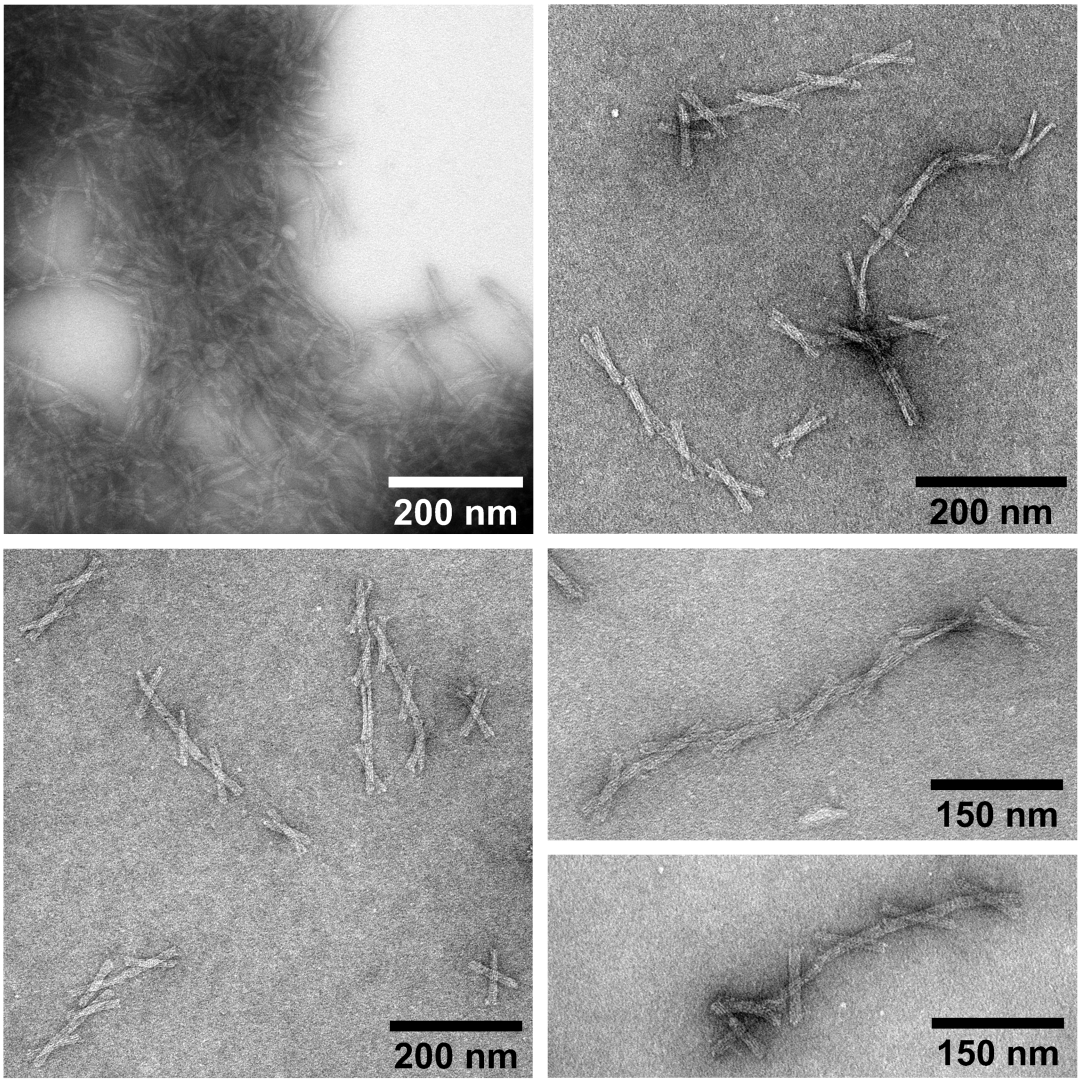

Figure S19. TEM images of linear 1D arrays formed in solution at pH 6.0 (24 h incubation at room temperature). The 1D chains were assembled using a DNA origami unit concentration of 2.0 nM. The TEM images are negatively stained with 2% (w/v) uranyl formate.

a)
b)

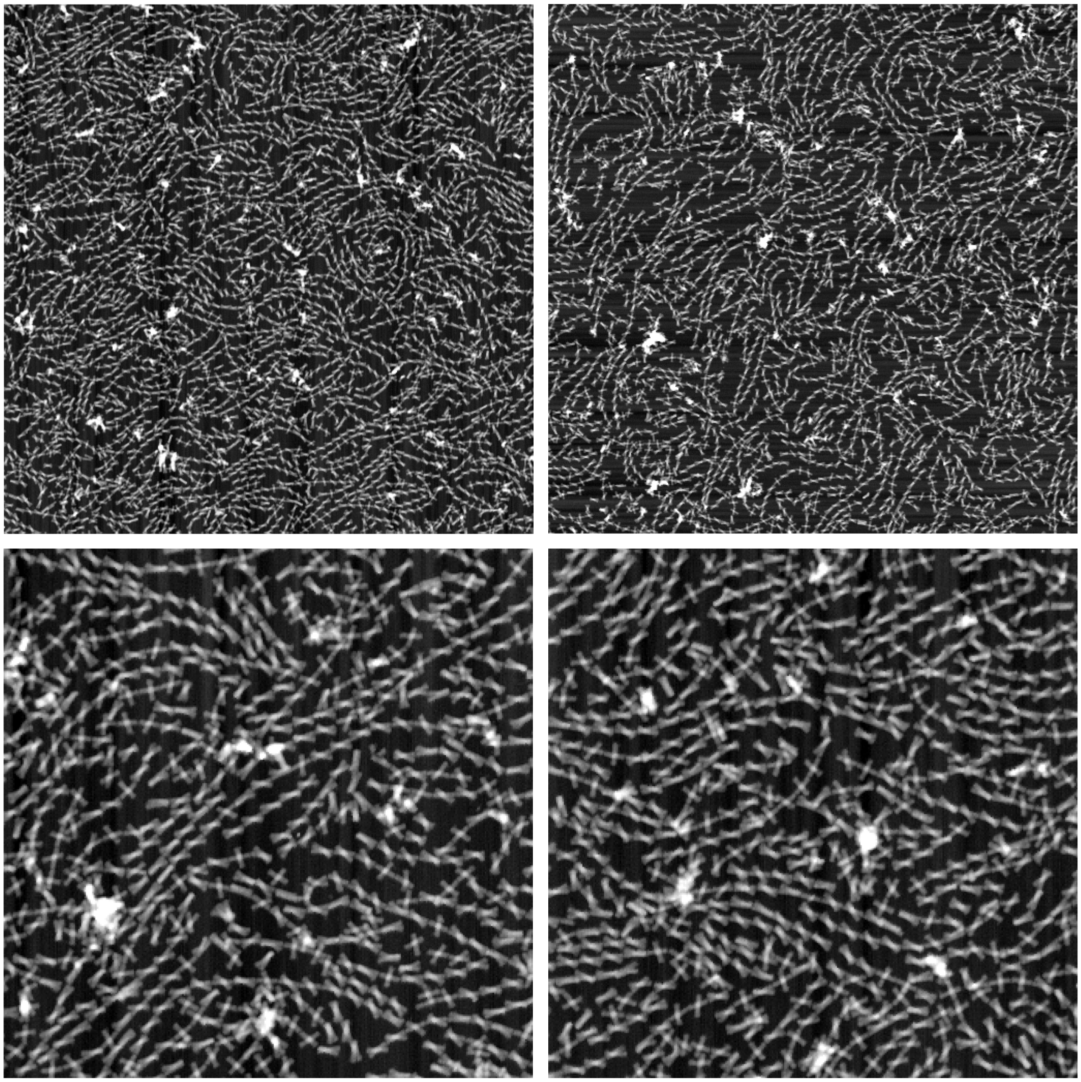

Figure S20. AFM images demonstrating the 1D array formation on the mica surface at pH 6.0. The DNA origami concentration in the sample is 2.0 nM and the assembly is done in 1$\times$ TAE containing 12.5 mM $MgCl_2$ and 75 mM NaCl. The lattice was formed on the mica surface during 3 h. (a) Large-scale AFM image (5 μm $\times$ 5 μm) of the formed linear chains. (b) Magnified AFM images (2 μm $\times$ 2 μm) of the assembled 1D arrays.

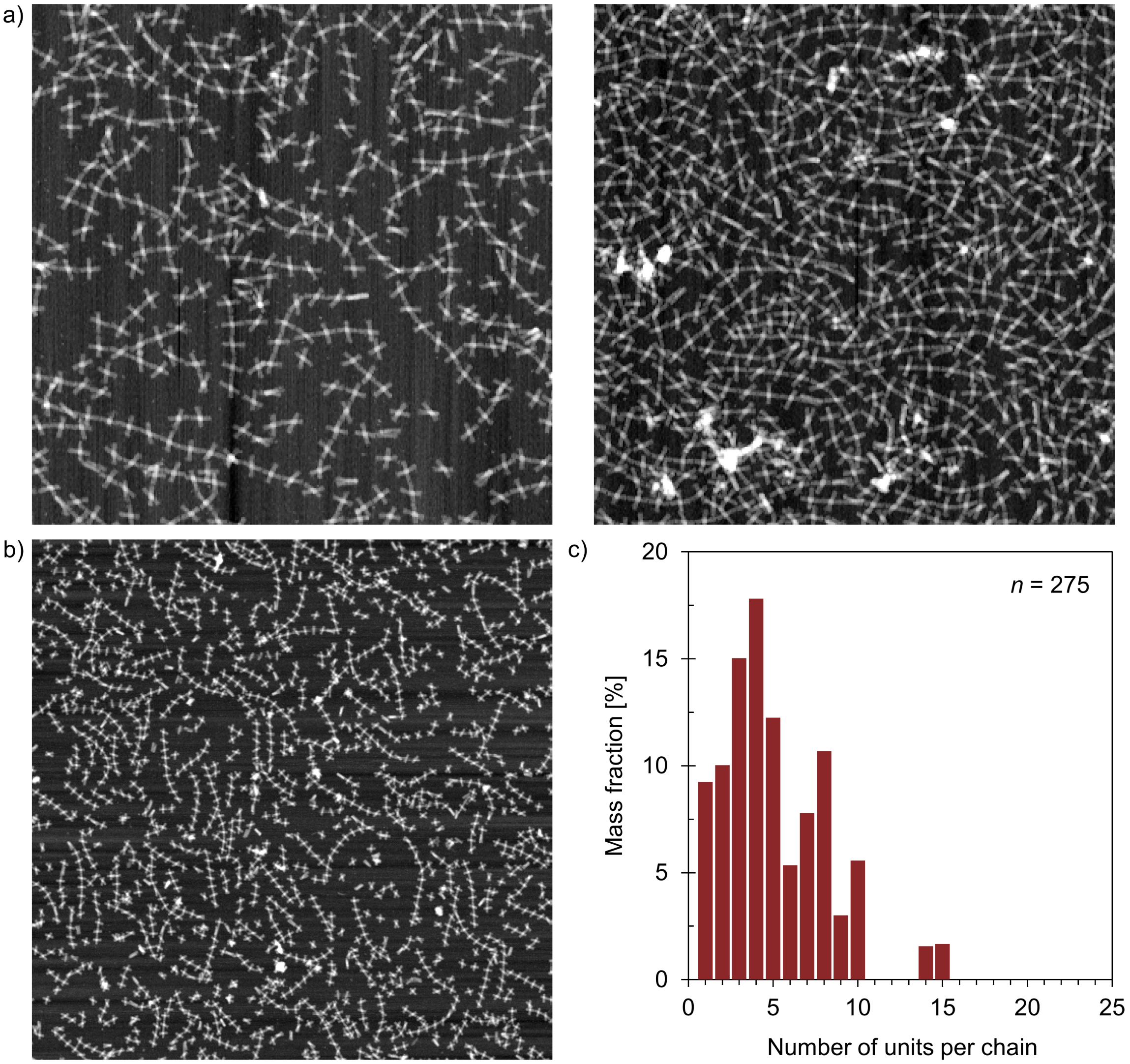

Figure S21. AFM images demonstrating the 1D array formation on the mica surface at pH 8.2 using the DNA origami unit with pH latches. The DNA origami concentration in the sample is 2.0 nM and the assembly is done in 1$\times$ TAE containing 10 mM $MgCl_2$ and 75 mM NaCl. The lattice was formed on the mica surface during 3 h. (a) AFM images (2 μm $\times$ 2 μm) of the assembled 1D arrays. (b) Large-scale AFM image (5 μm $\times$ 5 μm) of the formed linear chains. (c) Observed chain length distribution for the 1D arrays assembled at pH 8.2 (determined from AFM images).

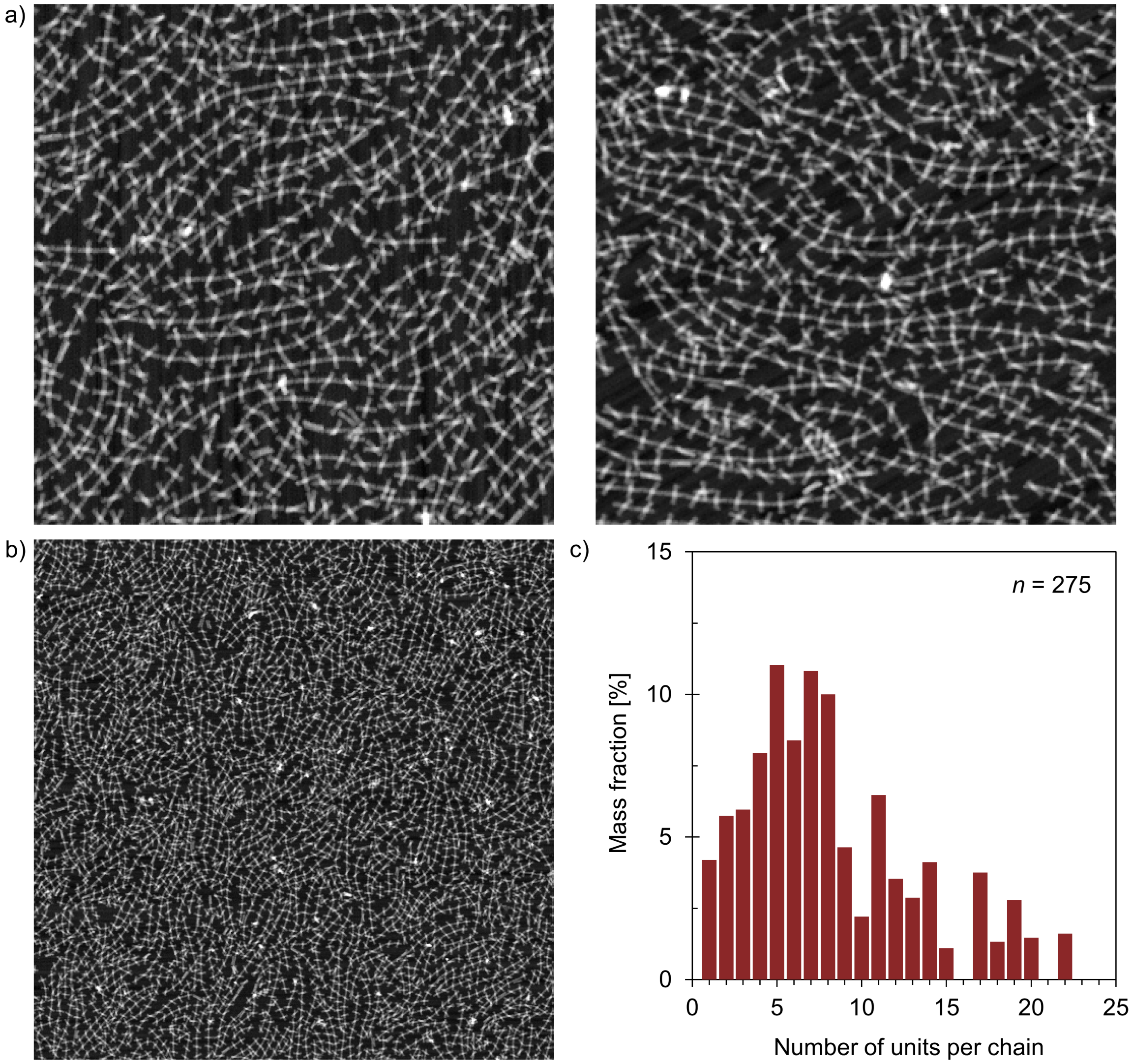

Figure S22. AFM images demonstrating the 1D array formation on the mica surface at pH 8.2 using the DNA origami unit with reverse pH latches. The DNA origami concentration in the sample is 2.0 nM and the assembly is done in 1× TAE containing 10 mM $MgCl_2$ and 75 mM NaCl. The lattice was formed on the mica surface during 5 h. (a) AFM images (2 μm × 2 μm) of the assembled 1D arrays. (b) Large-scale AFM image (5 μm × 5 μm) of the formed linear chains. (c) Observed chain length distribution for the 1D arrays assembled at pH 8.2 (determined from AFM images).

6. pH-Responsive and Reconfigurable 2D DNA Origami Lattices

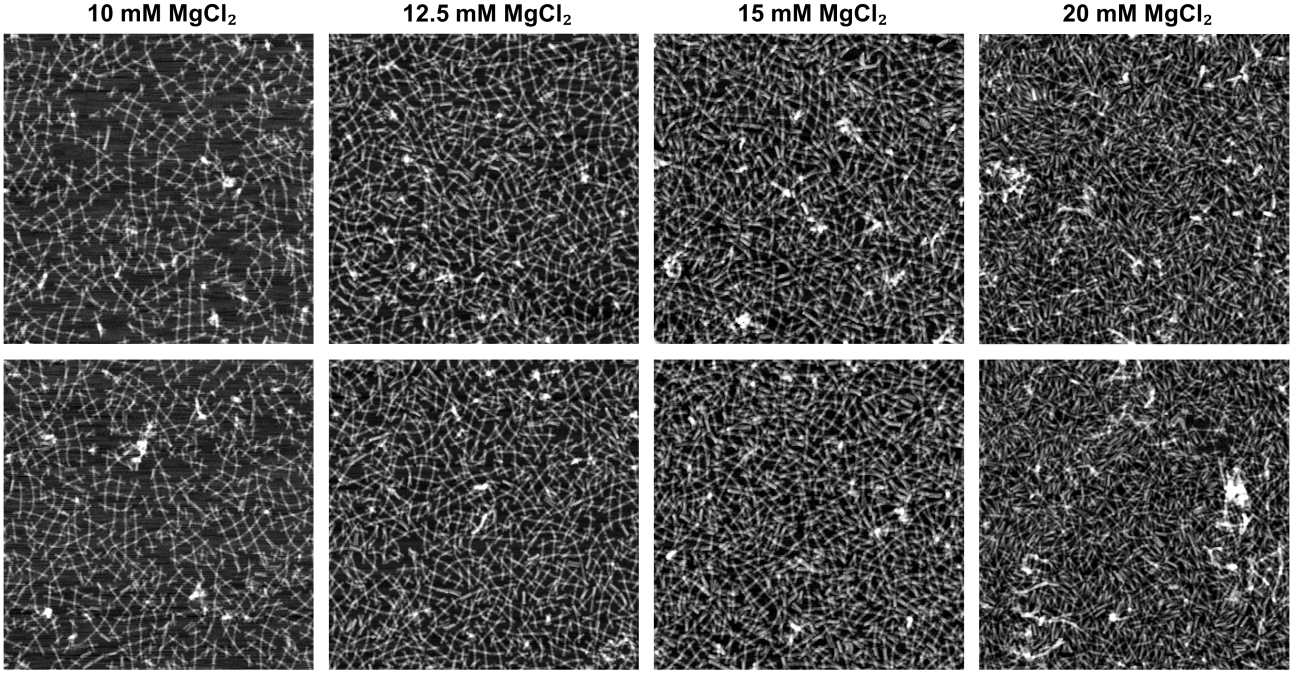

Figure S23. AFM images demonstrating the 2D lattice formation on the mica surface at pH 8.2 using the DNA origami unit with pH latches and different $MgCl_2$ concentrations. The DNA origami concentration in the sample is 2.0 nM and the assembly is done in 1× TAE containing $MgCl_2$ and 75 mM NaCl. The lattice was formed on the mica surface during 3 h. The size of the images is 2 μm × 2 μm.

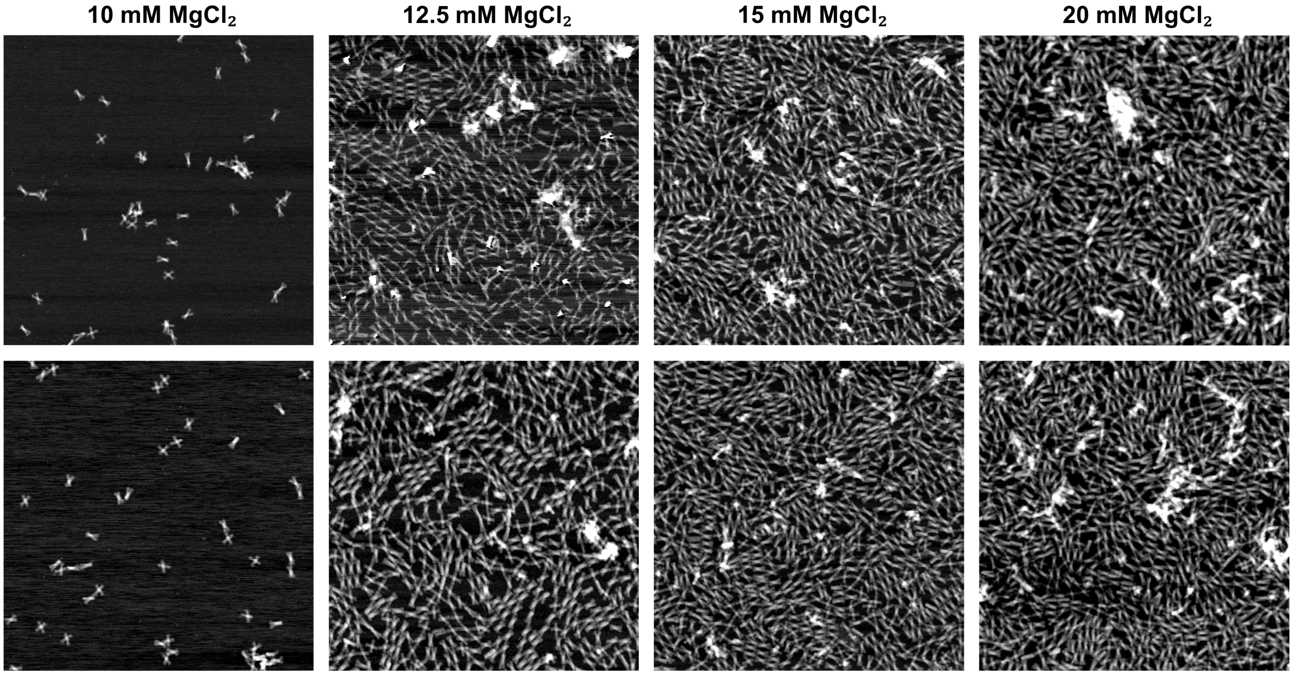

Figure S24. AFM images demonstrating the 2D lattice formation on the mica surface at pH 6.0 using the DNA origami unit with pH latches and different $MgCl_2$ concentrations. The DNA origami concentration in the sample is 2.0 nM and the assembly is done in $1\times$ TAE containing $MgCl_2$ and 75 mM NaCl. The lattice was formed on the mica surface during 3 h. The size of the images is 2 μm $\times$ 2 μm.

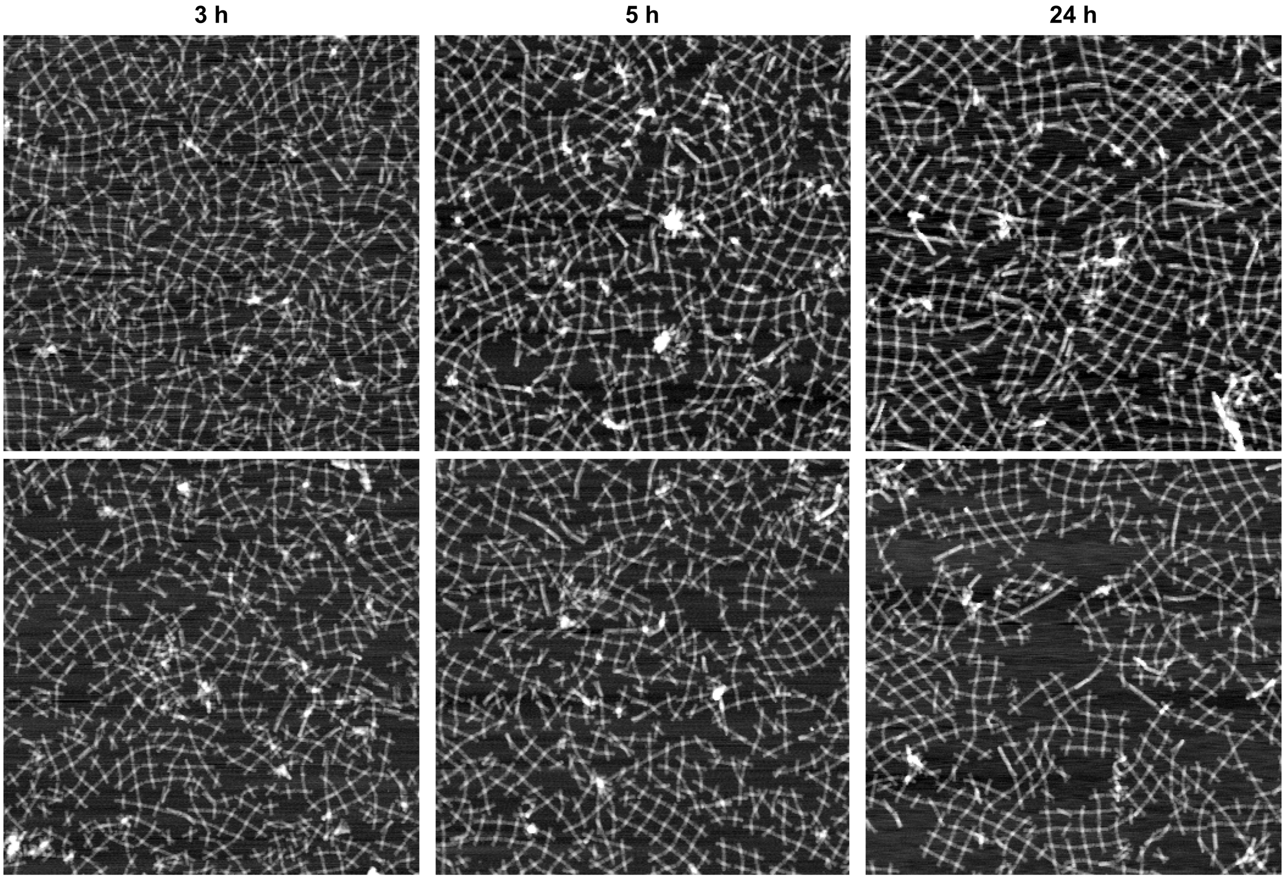

Figure S25. AFM images demonstrating the 2D lattice formation on mica at pH 8.2 using the DNA origami unit with pH latches and different incubation times. The DNA origami concentration in the sample is 2.0 nM and the assembly is done in 1× TAE containing 10 mM $MgCl_2$ and 75 mM NaCl. The size of the images is 2 μm × 2 μm.

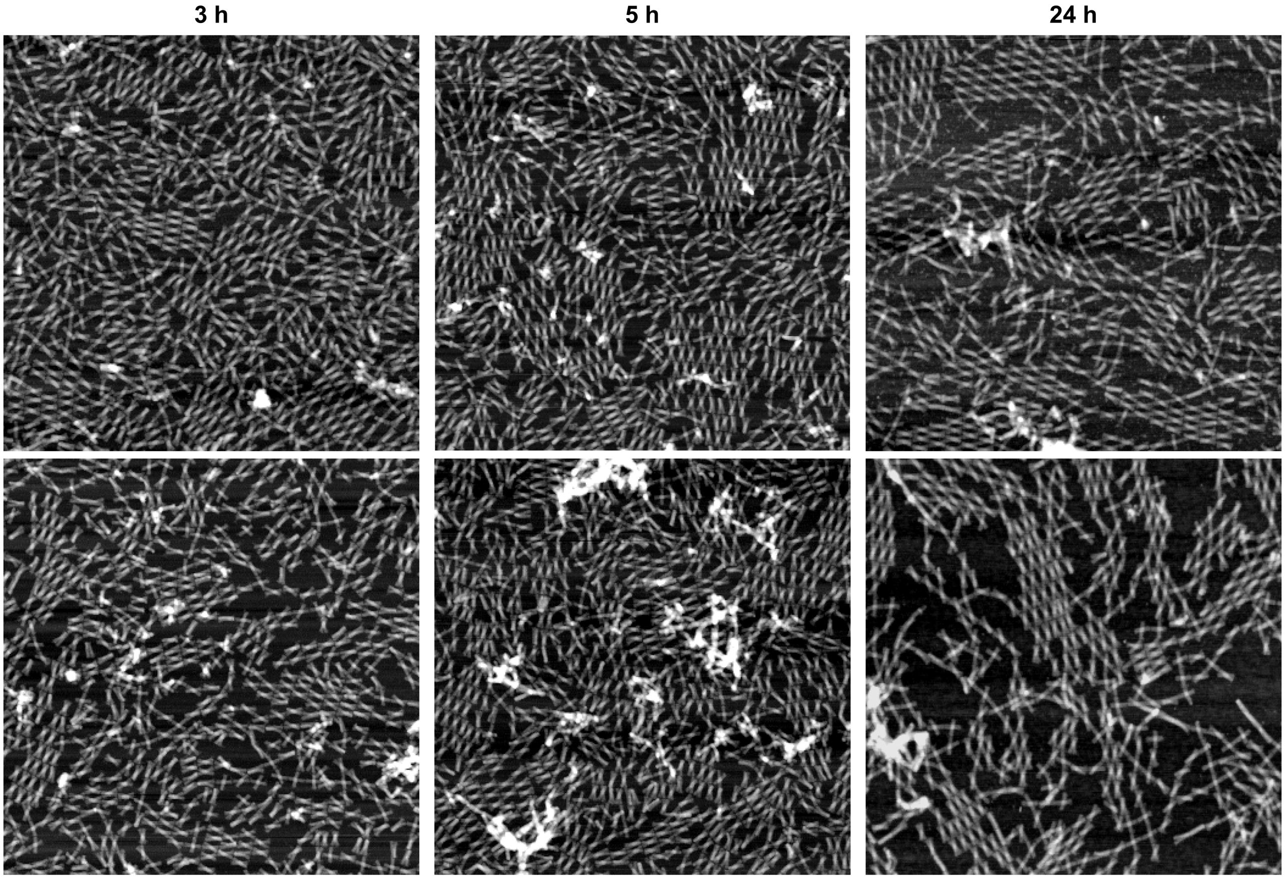

Figure S26. AFM images demonstrating the 2D lattice formation on mica at pH 6.0 using the DNA origami unit with pH latches and different incubation times. The DNA origami concentration in the sample is 2.0 nM and the assembly is done in 1× TAE containing 12.5 mM $MgCl_2$ and 75 mM NaCl. The size of the images is 2 μm × 2 μm.

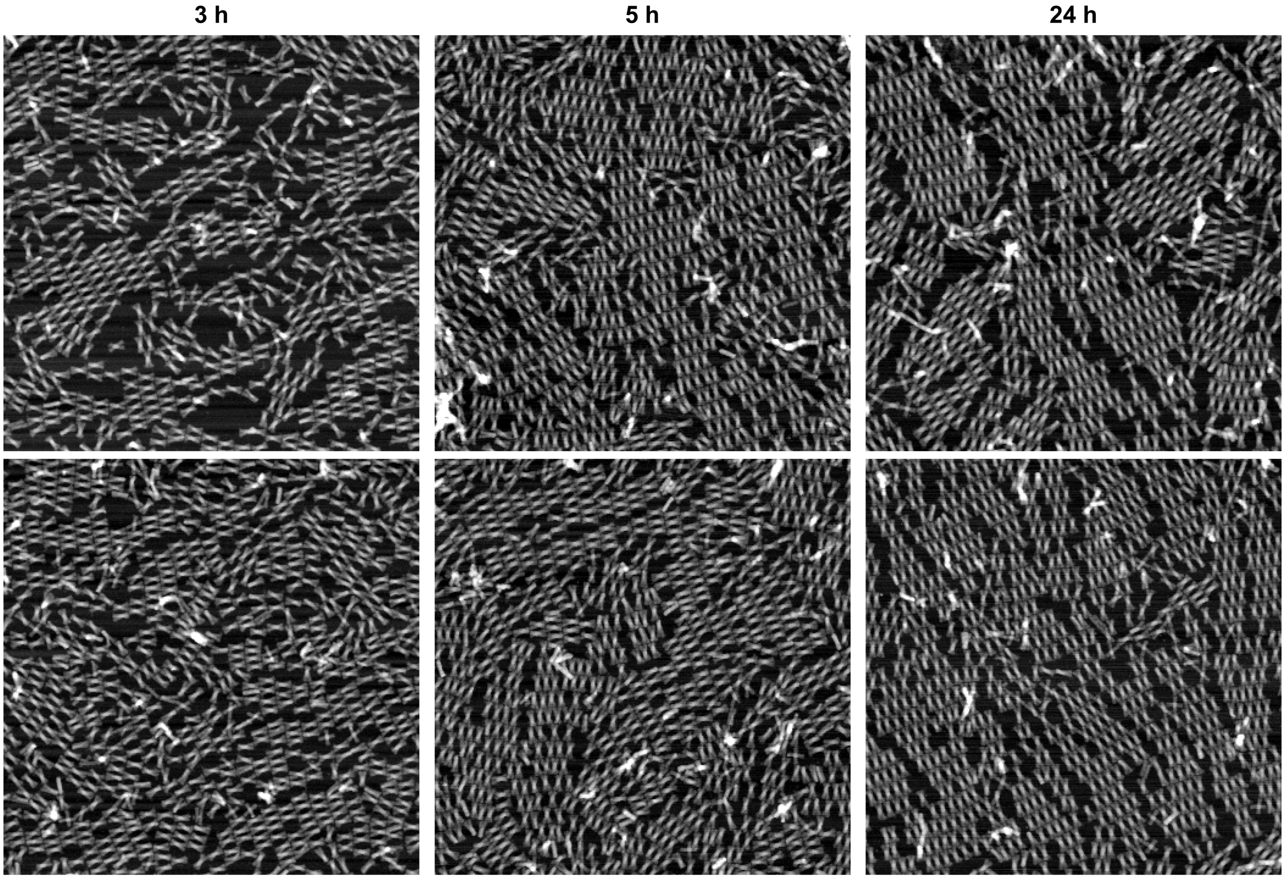

Figure S27. AFM images demonstrating the 2D lattice formation on the mica surface at pH 8.2 using the permanently closed DNA origami unit and different incubation times. The DNA origami concentration in the sample is 2.0 nM and the assembly is done in 1× TAE containing 10 mM $MgCl_2$ and 75 mM NaCl. The size of the images is 2 μm × 2 μm.

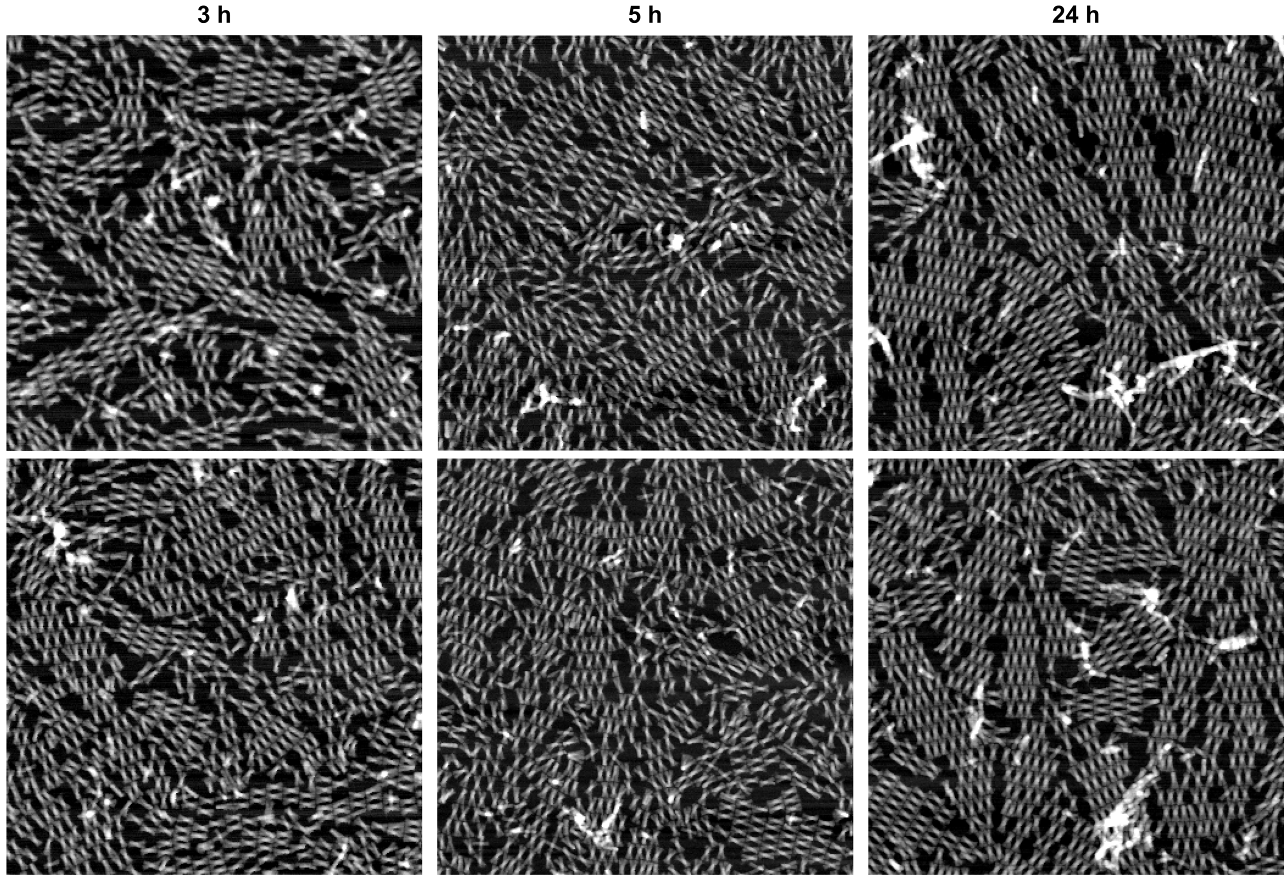

Figure S28. AFM images demonstrating the 2D lattice formation on the mica surface at pH 6.0 using the permanently closed DNA origami unit and different incubation times. The DNA origami concentration in the sample is 2.0 nM and the assembly is done in 1× TAE containing 12.5 mM $MgCl_2$ and 75 mM NaCl. The size of the images is 2 μm × 2 μm.

a)

b)

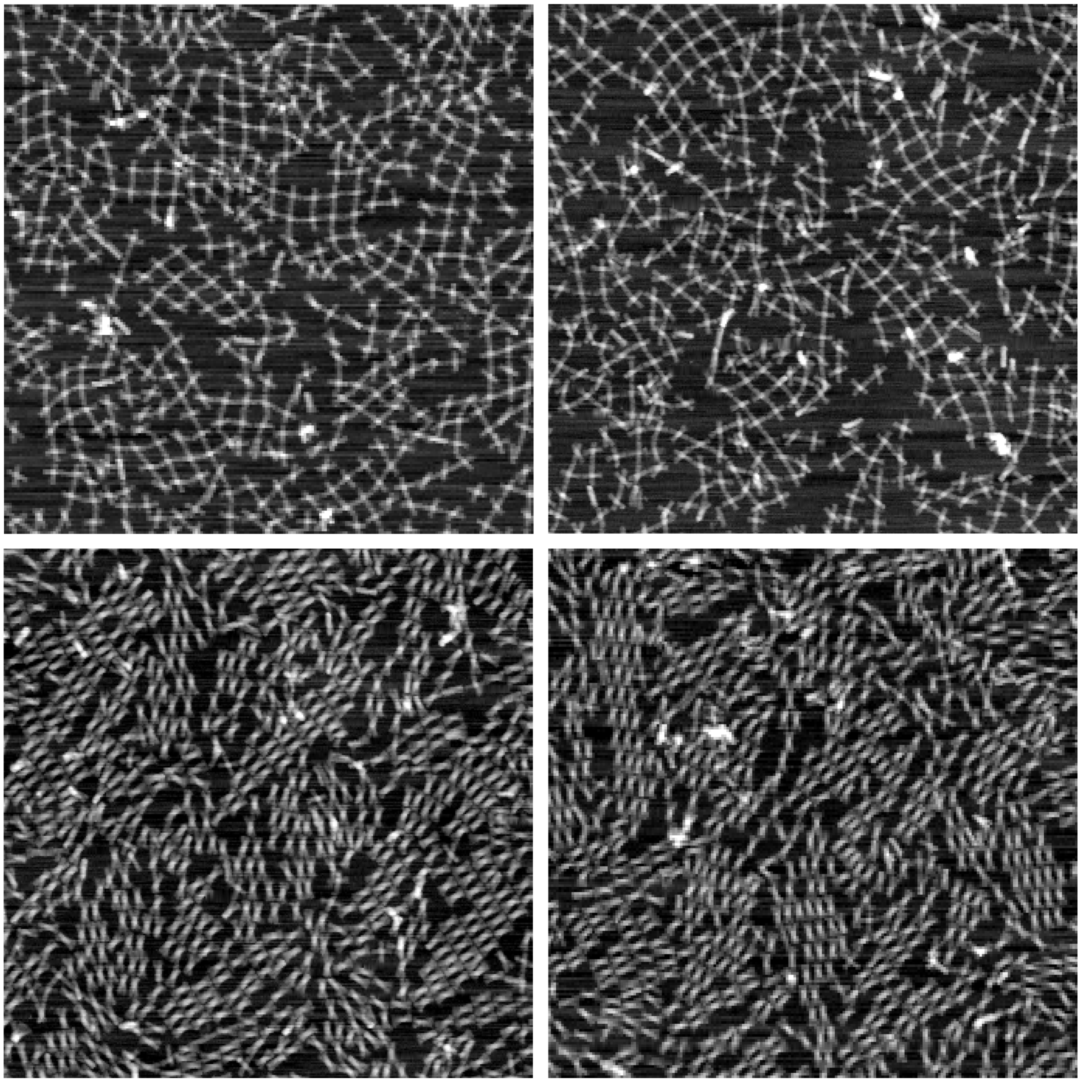

Figure S29. AFM images demonstrating the 2D lattice formation on mica using $CaCl_2$ instead of $MgCl_2$. (a) 2D lattice formation on mica at pH 8.2 using the DNA origami unit with pH latches. (b) 2D lattice formation on mica at pH 8.2 using the permanently closed DNA origami unit. The DNA origami concentration in the samples is 2.0 nM and the assembly is done in 1$\times$ TAE containing 10 mM $CaCl_2$ and 75 mM NaCl. The lattices were formed on the mica surface during 3 h. The size of the images is 2 μm $\times$ 2 μm.

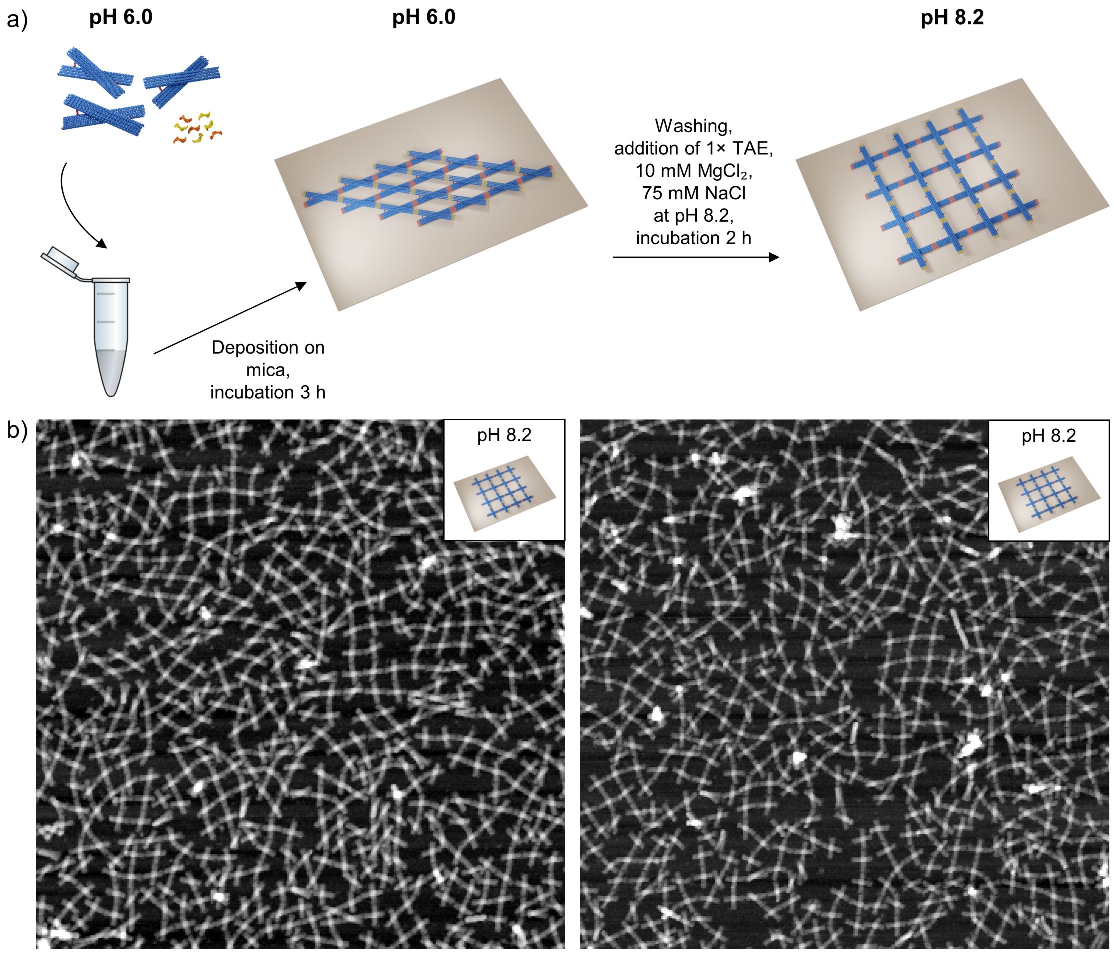

Figure S30. AFM images demonstrating the pH-responsiveness of the assembled lattices. (a) The lattices are assembled at pH 6.0 during 3 h. Weakly and unbound assemblies are washed away from the surface, after which 1$\times$ TAE, 10 mM $MgCl_2$ and 75 mM NaCl at pH 8.2 is deposited onto the surface to increase the pH to 8.2. To allow the lattice to rearrange, the sample is incubated for additional 2 h. (b) AFM images of the formed lattice after the pH has been increased to 8.2. The size of the images is 2 μm $\times$ 2 μm.

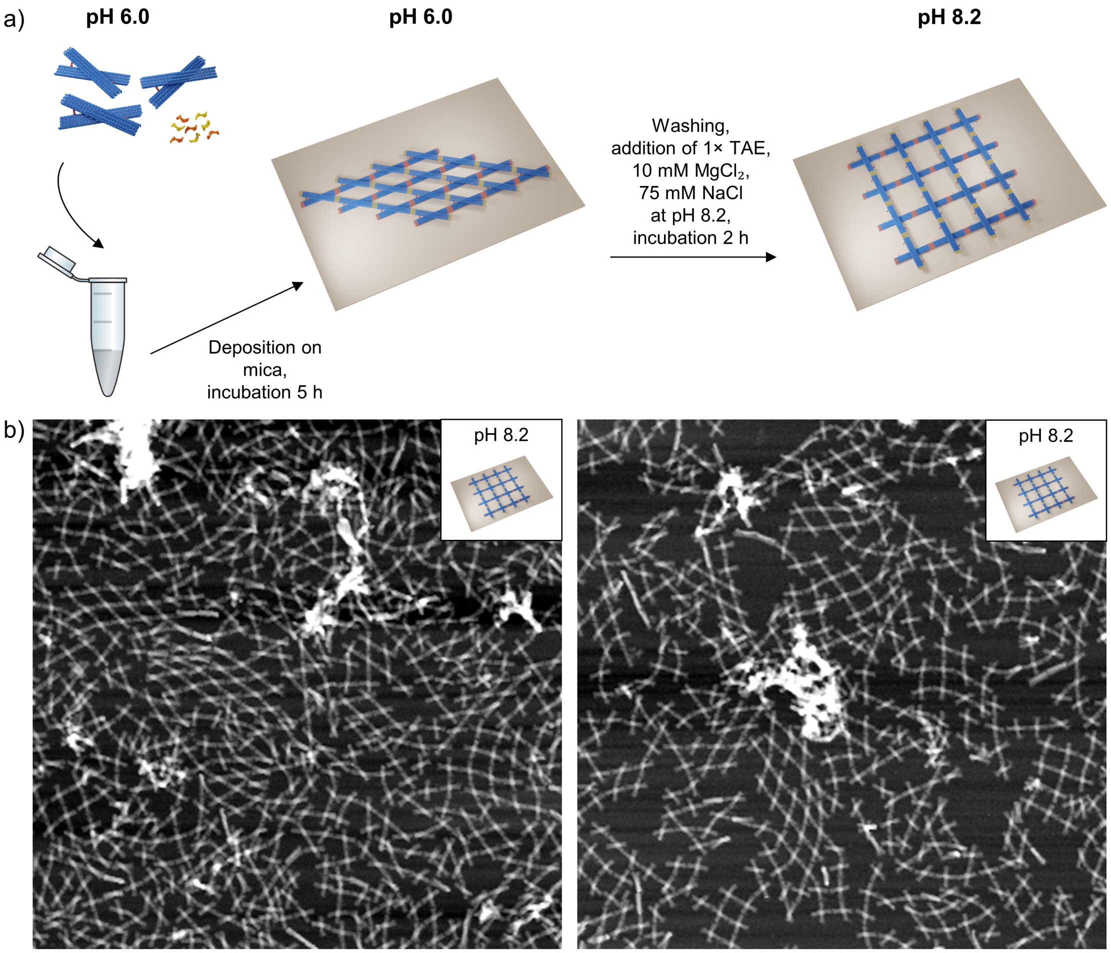

Figure S31. AFM images demonstrating the pH-responsiveness of the assembled lattices. (a) The lattices are assembled at pH 6.0 during 5 h. Weakly and unbound assemblies are washed away from the surface, after which 1× TAE, 10 mM $MgCl_2$ and 75 mM NaCl at pH 8.2 is deposited onto the surface to increase the pH to 8.2. To allow the lattice to rearrange, the sample is incubated for additional 2 h. (b) AFM images of the formed lattice after the pH has been increased to 8.2. The size of the images is 2 μm × 2 μm.

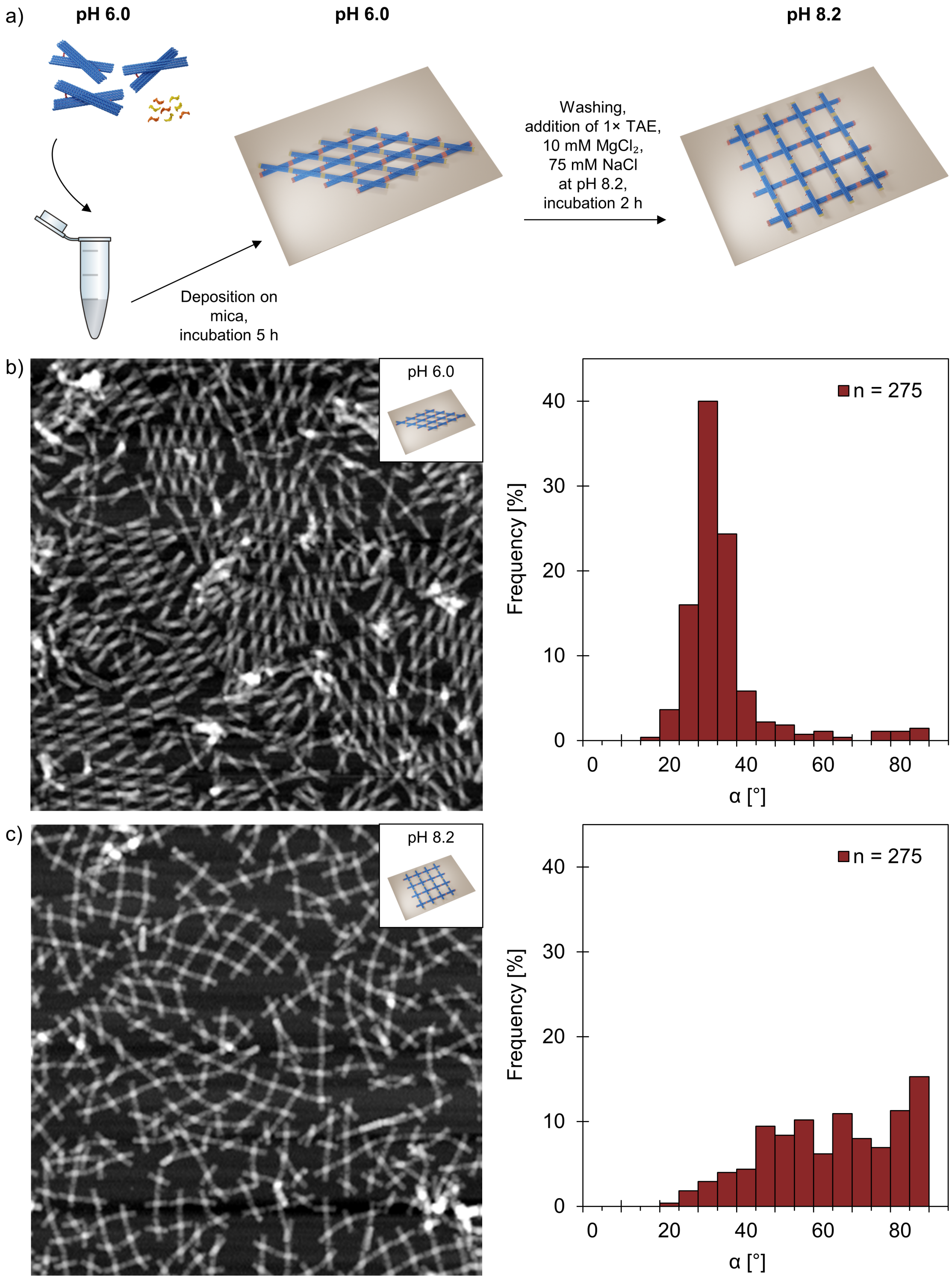

Figure S32. Statistical analysis of pH-responsive and reconfigurable 2D DNA origami lattices. (a) The lattices are assembled at pH 6.0 during 5 h. Weakly and unbound assemblies are washed away from the surface, after which 1× TAE, 10 mM $MgCl_2$ and 75 mM NaCl at pH 8.2 is deposited onto the surface to increase the pH to 8.2. To allow the lattice to rearrange, the sample is incubated for additional 2 h. AFM image and statistical analysis of (b) the formed lattice at pH 6.0 (c) the formed lattice after the pH has been increased to 8.2. The size of the AFM images in the right panel is 1.5 μm × 1.5 μm. The same AFM images are also shown in the article in Figure 4a. The right panel shows the distribution of the angle, α, between the two arms of the unit for units incorporated into lattices (have another unit connected to both of the arms between which the angle is measured). The angle distribution is determined from AFM images and the number of individual structures analyzed for each sample is *n* = 275.

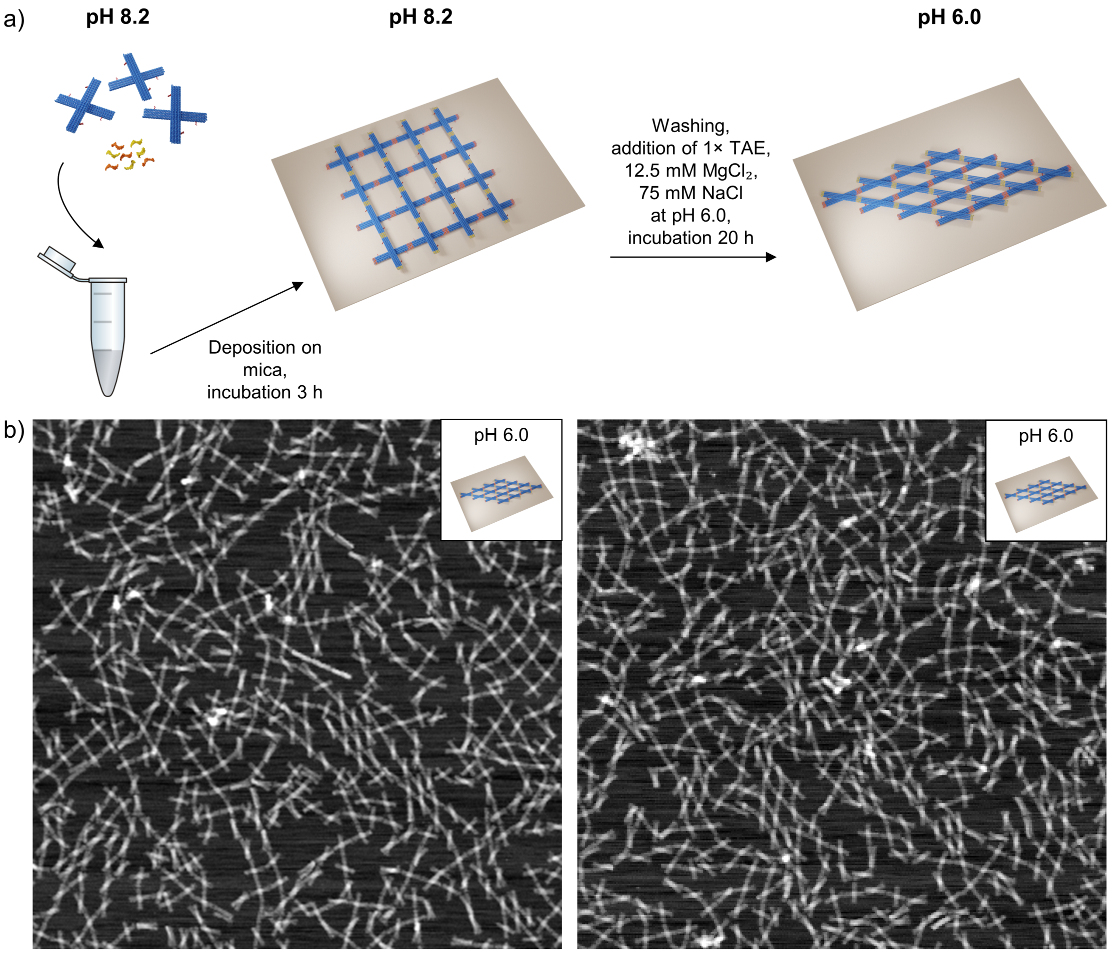

Figure S33. AFM images demonstrating the pH-responsiveness of the assembled lattices. (a) The lattices are assembled at pH 8.2 during 3 h. Weakly and unbound assemblies are washed away from the surface, after which 1× TAE, 12.5 mM $MgCl_2$ and 75 mM NaCl at pH 6.0 is deposited onto the surface to decrease the pH to 6.0. To allow the lattice to rearrange, the sample is incubated for additional 20 h. (b) AFM images of the formed lattice after the pH has been decreased to 6.0. The size of the images is 2 μm $\times$ 2 μm.

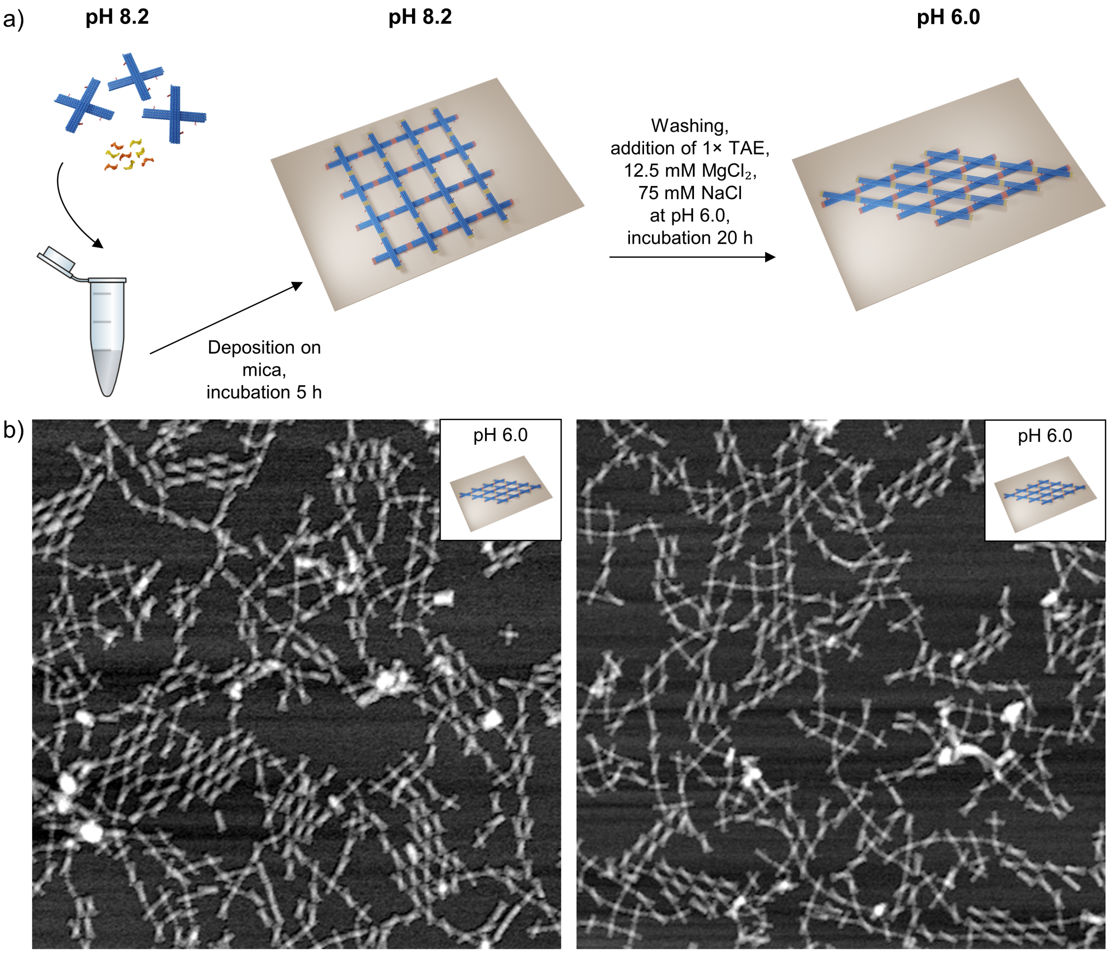

Figure S34. AFM images demonstrating the pH-responsiveness of the assembled lattices. (a) The lattices are assembled at pH 8.2 during 5 h. Weakly and unbound assemblies are washed away from the surface, after which 1× TAE, 12.5 mM $MgCl_2$ and 75 mM NaCl at pH 6.0 is deposited onto the surface to decrease the pH to 6.0. To allow the lattice to rearrange, the sample is incubated for additional 20 h. (b) AFM images of the formed lattice after the pH has been decreased to 6.0. The size of the images is 2 μm × 2 μm.

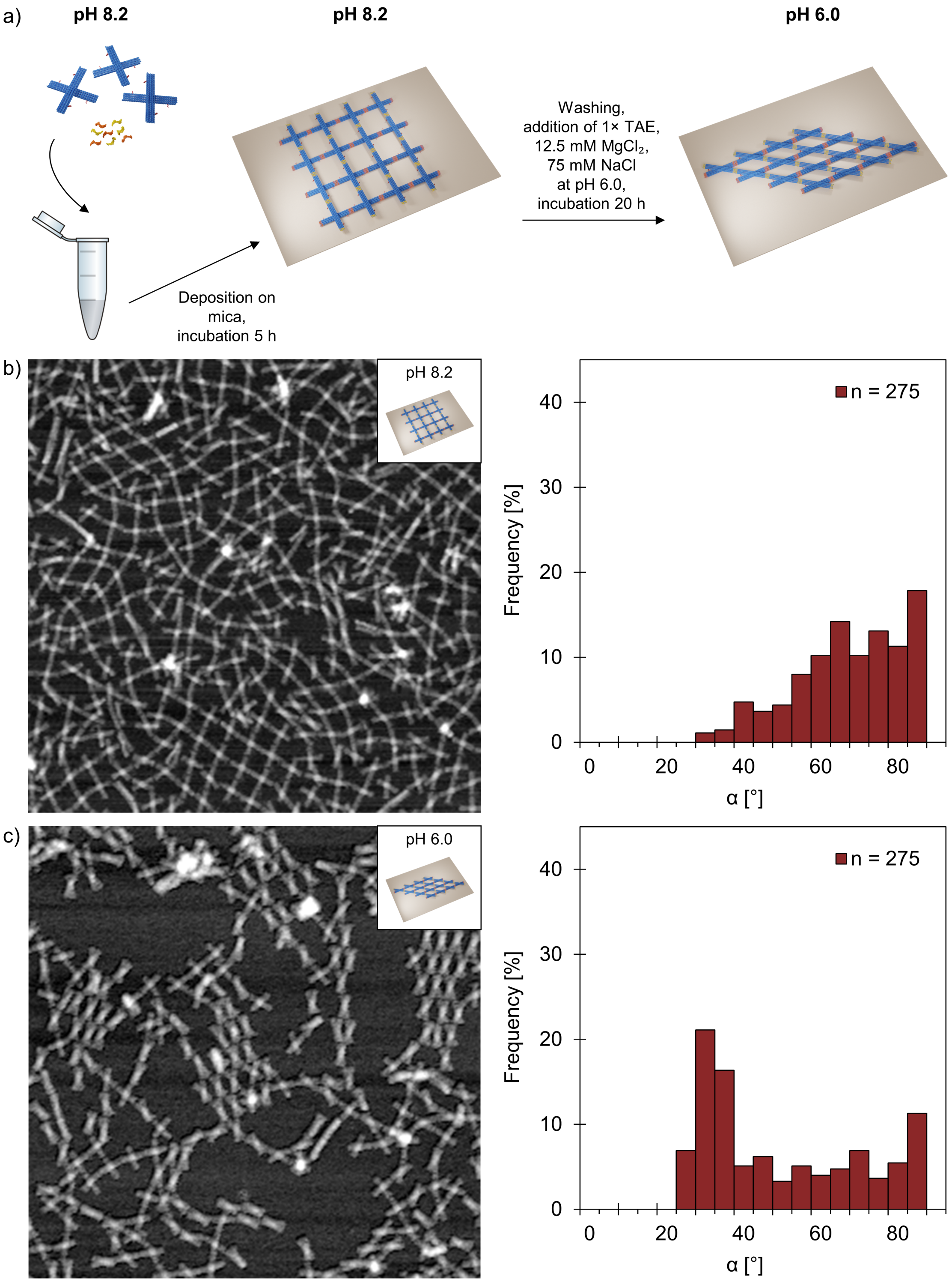

Figure S35. AFM images demonstrating the pH-responsiveness of the assembled lattices. (a) The lattices are assembled at pH 8.2 during 5 h. Weakly and unbound assemblies are washed away from the surface, after which 1 $\times$ TAE, 12.5 mM $MgCl_2$ and 75 mM NaCl at pH 6.0 is deposited onto the surface to decrease the pH to 6.0. To allow the lattice to rearrange, the sample is incubated for additional 20 h. AFM image and statistical analysis of (b) the formed lattice at pH 8.2 (c) the formed lattice after the pH has been increased to 6.0. The size of the AFM images in the right panel is 1.5 μm $\times$ 1.5 μm. The same AFM images are also shown in the article in Figure 4b. The right panel shows the distribution of the angle, α, between the two arms of the unit for units incorporated into lattices (have another unit connected to both of the arms between which the angle is measured). The angle distribution is determined from AFM images and the number of individual structures analyzed for each sample is n = 275.

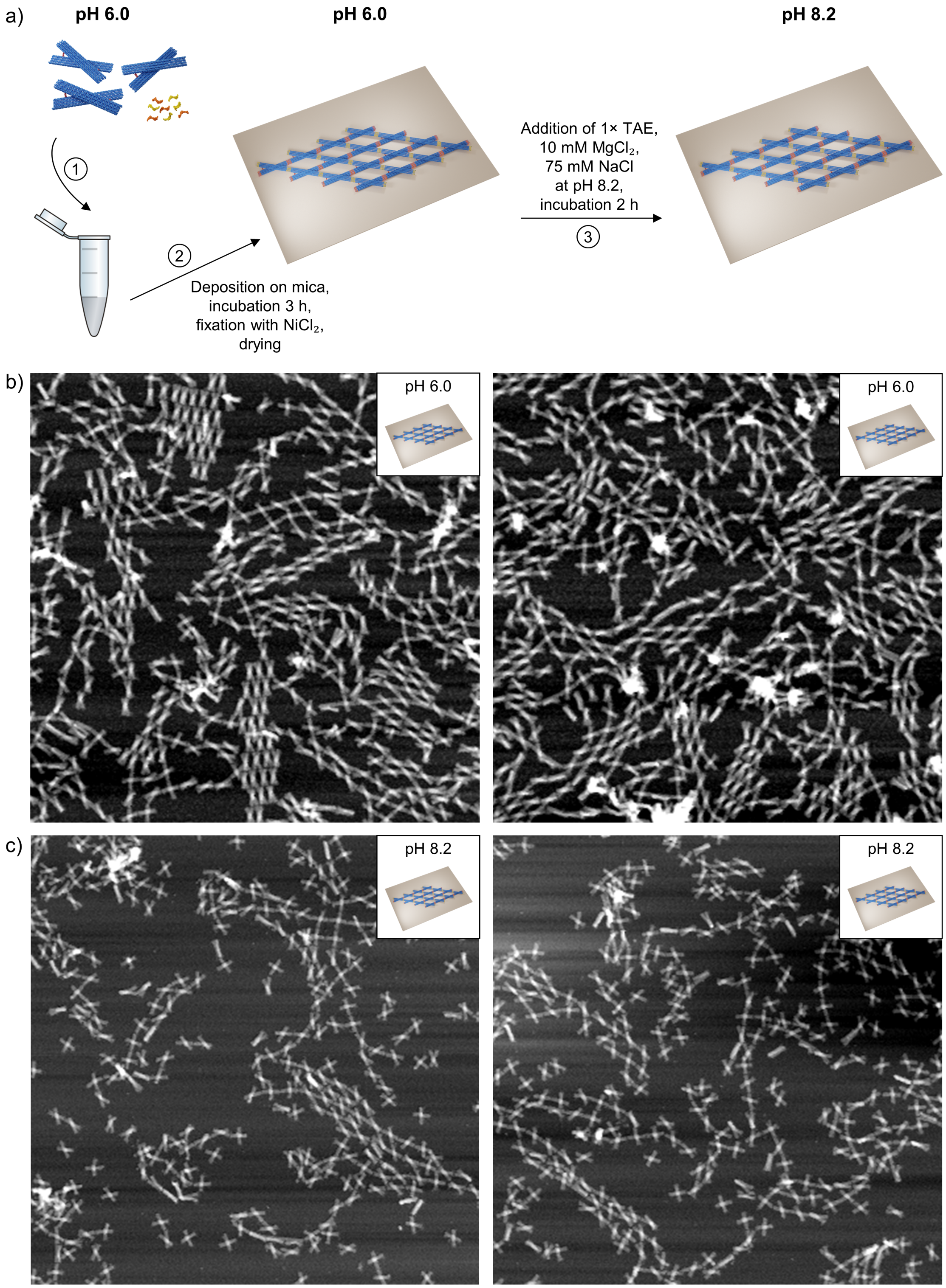

Figure S36. AFM images demonstrating that lattices attached to the mica substrate with $NiCl_2$ is not pH-responsive and reconfigurable if rehydrated. (a) The lattices are assembled at pH 6.0 during 3 h. Weakly and unbound assemblies are washed away from the surface, after which the sample is attached to the mica substrate with $NiCl_2$ and dried. 1× TAE, 10 mM $MgCl_2$ and 75 mM NaCl at pH 8.2 is deposited onto the surface to rehydrate the lattice and to increase the pH to 8.2. To allow the lattice to rearrange the sample is incubated for 2 h. (b) AFM images of lattices assembled at pH 6.0 during 3 h (after step 2 in the schematics). The lattices are attached to the mica substrate with $NiCl_2$. (c) AFM images of the same sample after rehydration with 1× TAE, 10 mM $MgCl_2$ and 75 mM NaCl at pH 8.2 for 2 h (after step 3 in the schematics). The lattices are attached to the mica substrate with $NiCl_2$ before drying and rehydration. Therefore, the lattices will not change its conformation even if the pH is increased. The size of the images is 2 μm × 2 μm.

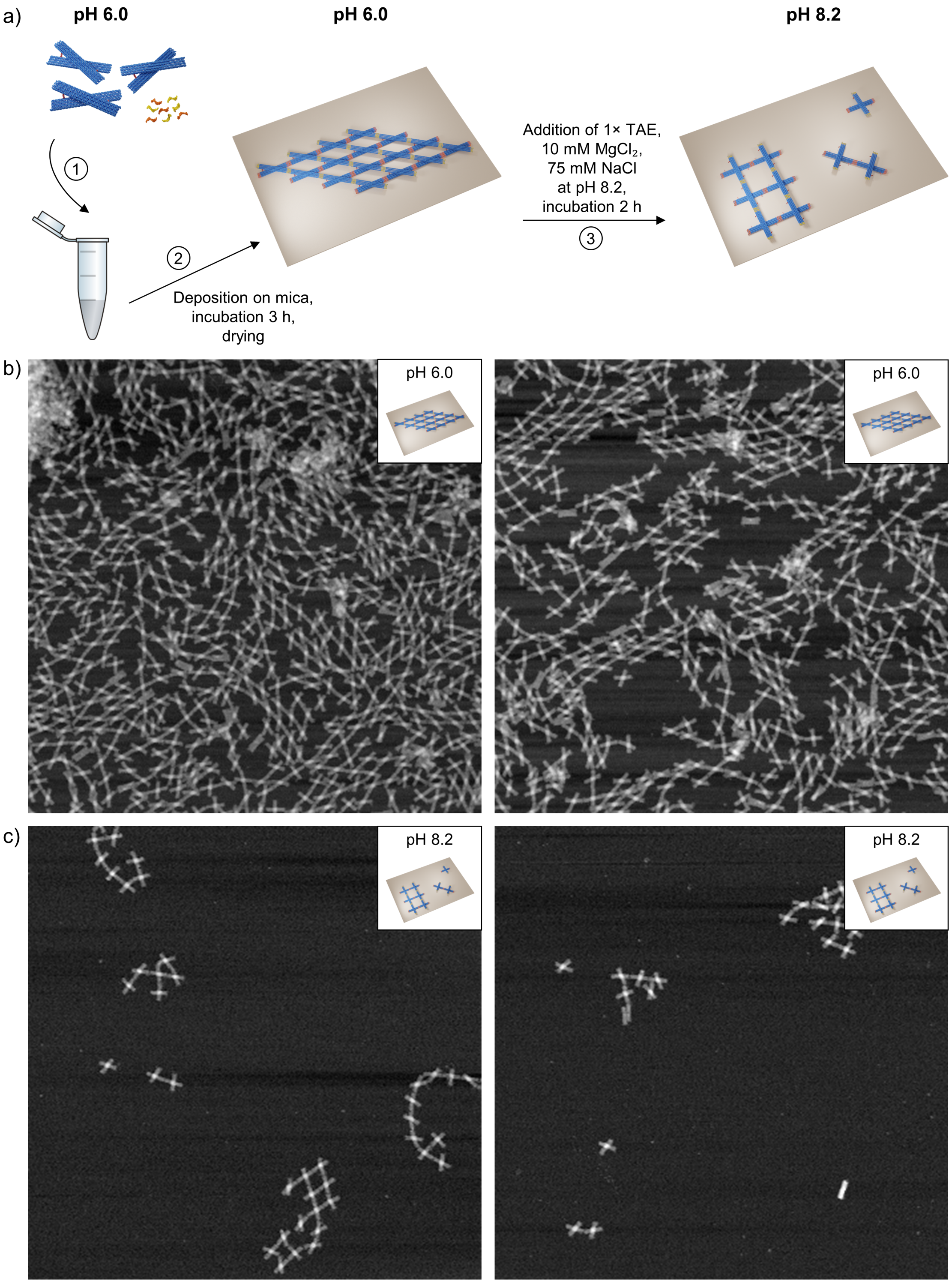

Figure S37. AFM images demonstrating the pH-responsiveness and reconfigurability of rehydrated lattices. (a) The lattices are assembled at pH 6.0 during 3 h. Weakly and unbound assemblies are washed away from the surface, after which the sample is dried. 1× TAE, 10 mM $MgCl_2$ and 75 mM NaCl at pH 8.2 is deposited onto the surface to rehydrate the lattice and to increase the pH to 8.2. To allow the lattice to rearrange the sample is incubated for 2 h. (b) AFM images of lattices assembled at pH 6.0 during 3 h (after step 2 in the schematics). The lattices are not attached to the mica substrate with $NiCl_2$. (c) AFM images of the same sample after rehydration with 1× TAE, 10 mM $MgCl_2$ and 75 mM NaCl at pH 8.2 for 2 h (after step 3 in the schematics). This relative amount of Na^+ and Mg^{2+} in the rehydration buffer is not sufficient to keep the DNA origami adsorbed to the surface after the rehydration. The size of the images is 2 μm × 2 μm.

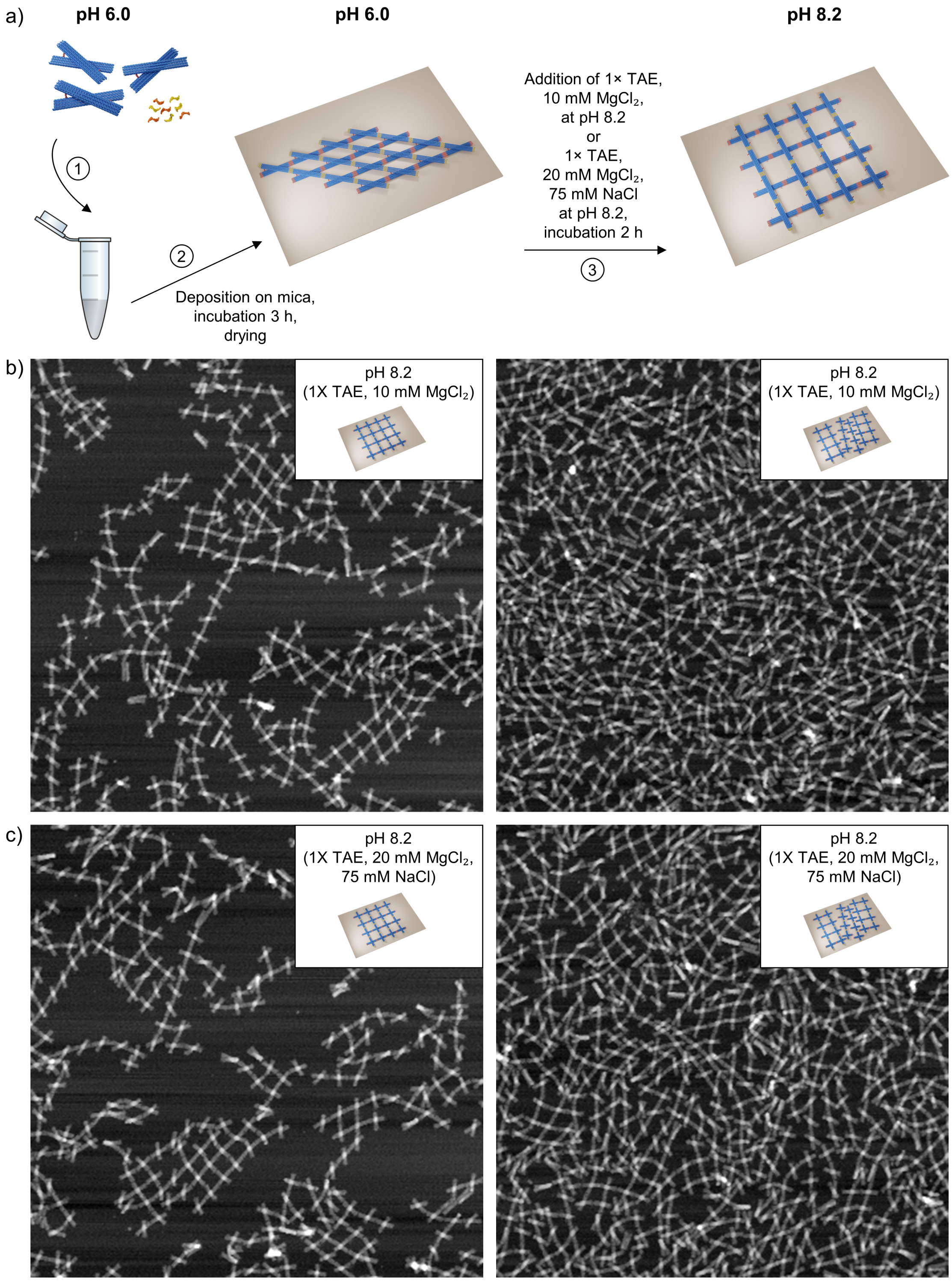

Figure S38. AFM images demonstrating the pH-responsiveness and reconfigurability of rehydrated lattices. (a) The lattices are assembled at pH 6.0 during 3 h. Weakly and unbound assemblies are washed away from the surface, after which the sample is dried. 1× TAE and 10 mM $MgCl_2$ at pH 8.2 or 1× TAE, 20 mM $MgCl_2$ and 75 mM NaCl at pH 8.2 is deposited onto the surface to rehydrate the lattice and to increase the pH to 8.2. To allow the lattice to rearrange the sample is incubated for 2 h. (b) AFM images of the sample after rehydration with 1× TAE and 10 mM $MgCl_2$ at pH 8.2 for 2 h (after step 3 in the schematics). (c) AFM images of the sample after rehydration with 1× TAE, 20 mM $MgCl_2$ and 75 mM NaCl at pH 8.2 for 2 h (after step 3 in the schematics). The lattices are not attached to the mica substrate with $NiCl_2$ before drying and rehydration. Therefore, the lattices have a tendency to move on the substrate and come closely together during the washing step (shown in the AFM images in the right panel). The size of the images is 2 μm × 2 μm.

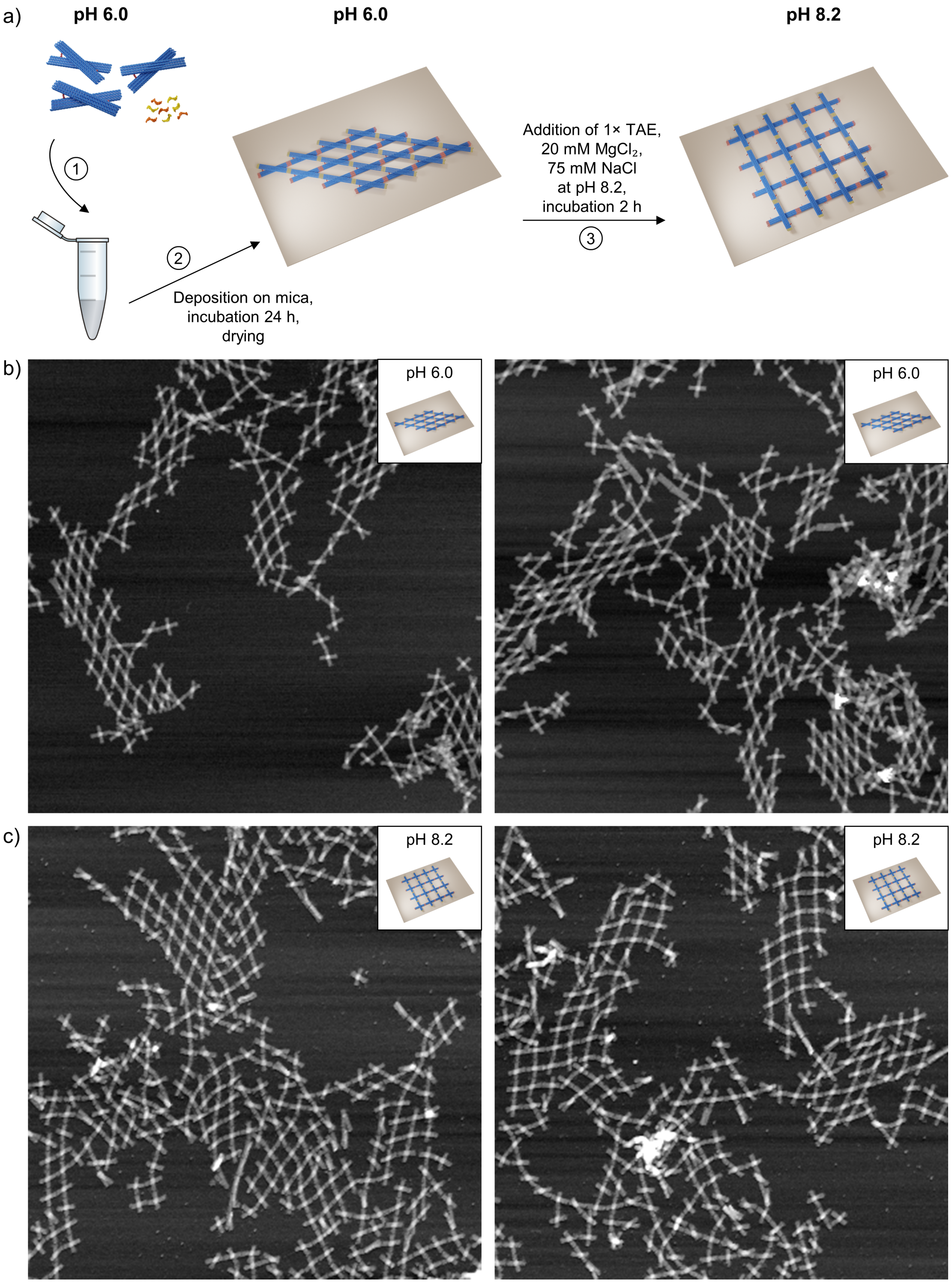

Figure S39. AFM images demonstrating the pH-responsiveness and reconfigurability of rehydrated lattices. (a) The lattices are assembled at pH 6.0 during 24 h. Weakly and unbound assemblies are washed away from the surface, after which the sample is dried. 1× TAE, 20 mM $MgCl_2$ and 75 mM NaCl at pH 8.2 is deposited onto the surface to rehydrate the lattice and to increase the pH to 8.2. To allow the lattice to rearrange the sample is incubated for 2 h. (b) AFM images of lattices assembled at pH 6.0 during 24 h (after step 2 in the schematics). The lattices are not attached to the mica substrate with $NiCl_2$. (c) AFM images of the same sample after rehydration with 1× TAE, 20 mM $MgCl_2$ and 75 mM NaCl at pH 8.2 for 2 h (after step 3 in the schematics). The size of the images is 2 μm × 2 μm.

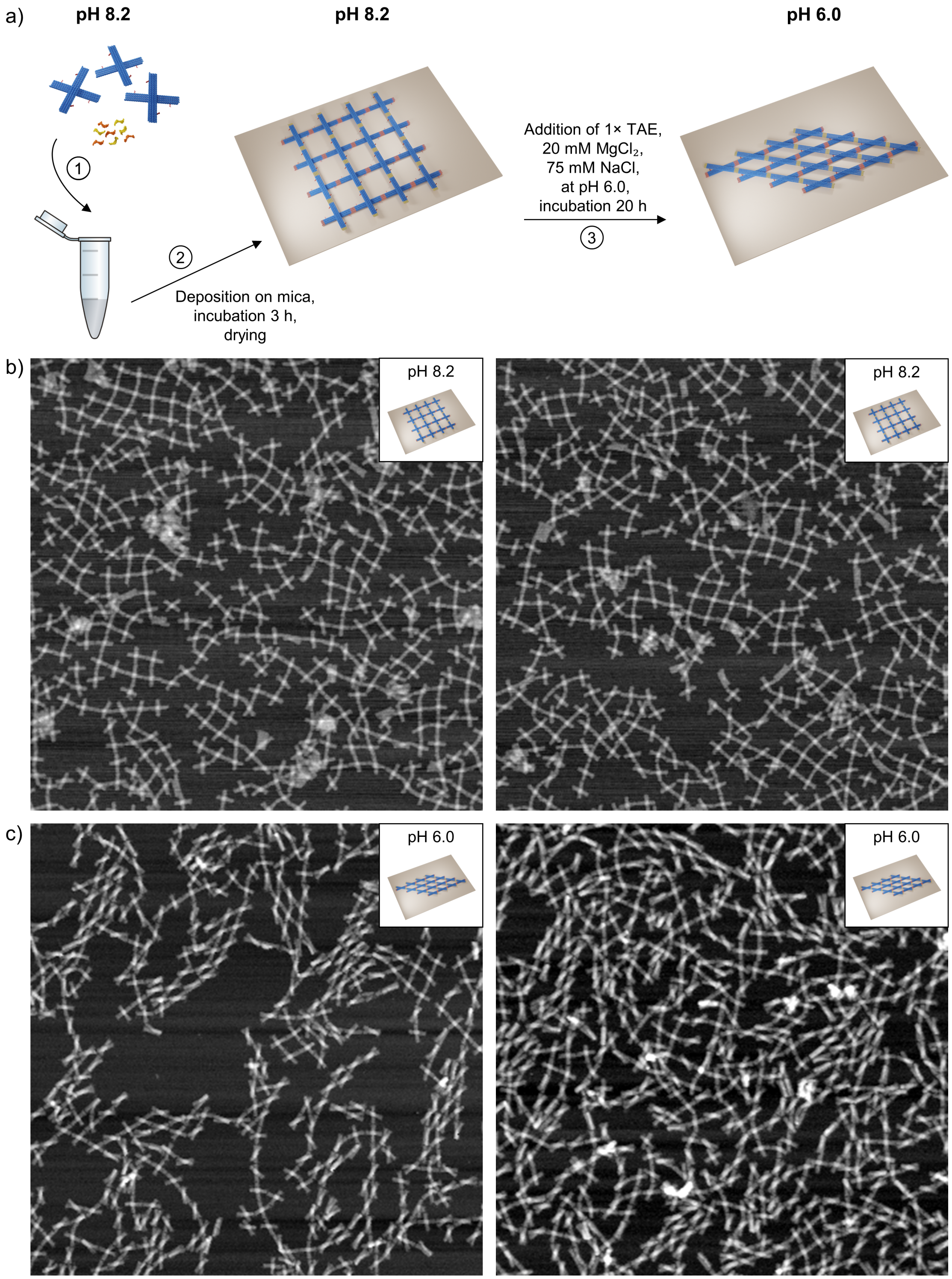

Figure S40. AFM images demonstrating the pH-responsiveness and reconfigurability of rehydrated lattices. (a) The lattices are assembled at pH 8.2 during 3 h. Weakly and unbound assemblies are washed away from the surface, after which the sample is dried. $1\times$ TAE, 20 mM $MgCl_2$ and 75 mM NaCl at pH 6.0 is deposited onto the surface to rehydrate the lattice and to decrease the pH to 6.0. To allow the lattice to rearrange the sample is incubated for 20 h. (b) AFM images of lattices assembled at pH 8.2 during 3 h (after step 2 in the schematics). The lattices are not attached to the mica substrate with $NiCl_2$. (c) AFM images of the same sample after rehydration with $1\times$ TAE, 20 mM $MgCl_2$ and 75 mM NaCl at pH 6.0 for 20 h (after step 3 in the schematics). The size of the images is 2 μm $\times$ 2 μm.

7. DNA-Templated, pH-Responsive, and Reconfigurable 2D AuNP Lattices

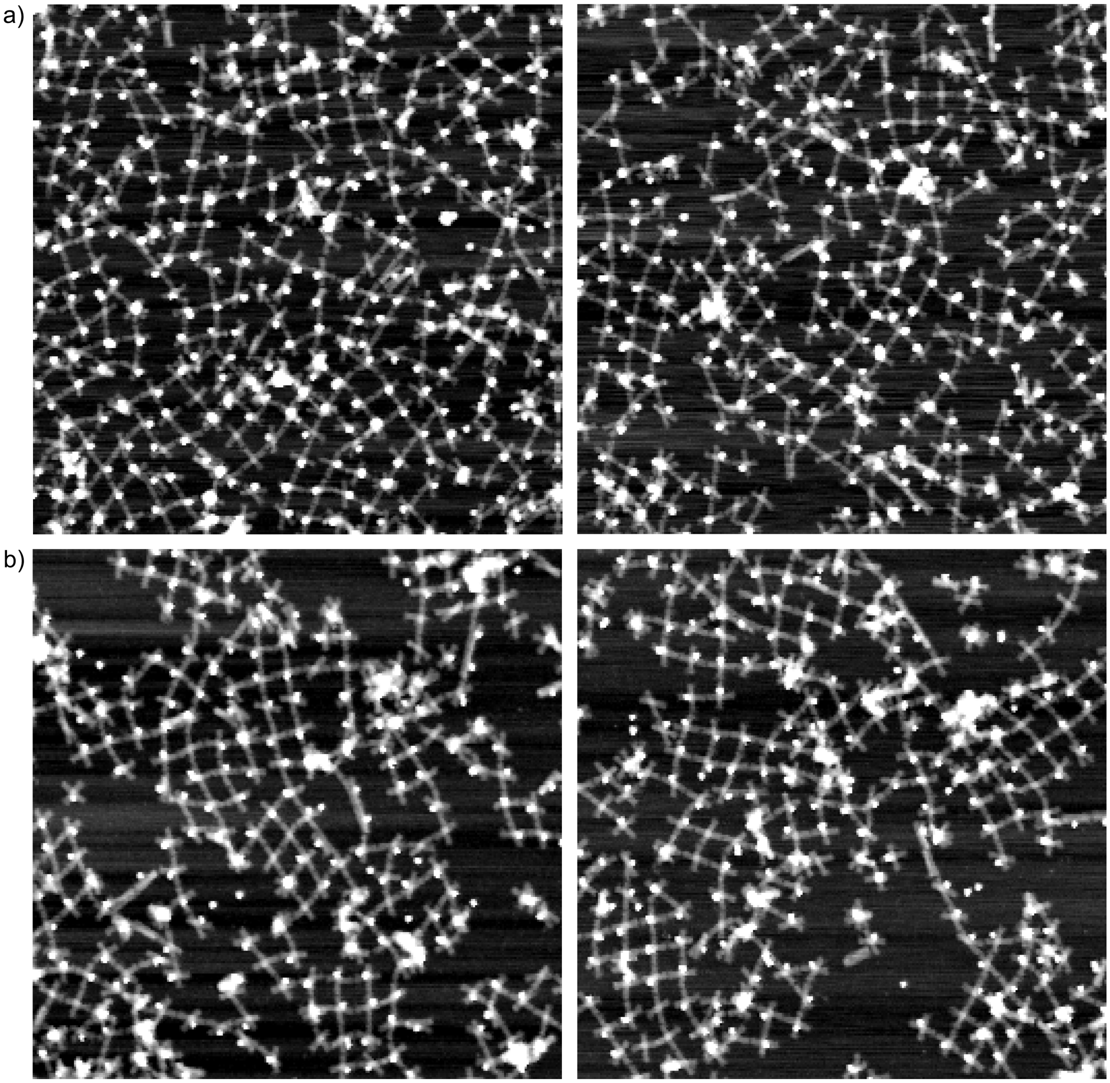

Figure S41. AFM images of AuNP lattices formed on a mica substrate at pH 8.2 using the DNA origami unit with pH latches. The assembly is done in 1× TAE containing 10 mM $MgCl_2$ and 75 mM NaCl. The size of the images is 1.5 μm × 1.5 μm. The DNA origami concentration in the samples is (a) 2.0 nM and (b) 3.0 nM (calculated based on the concentration in the conjugation step, assuming no loss during the PEG purification).

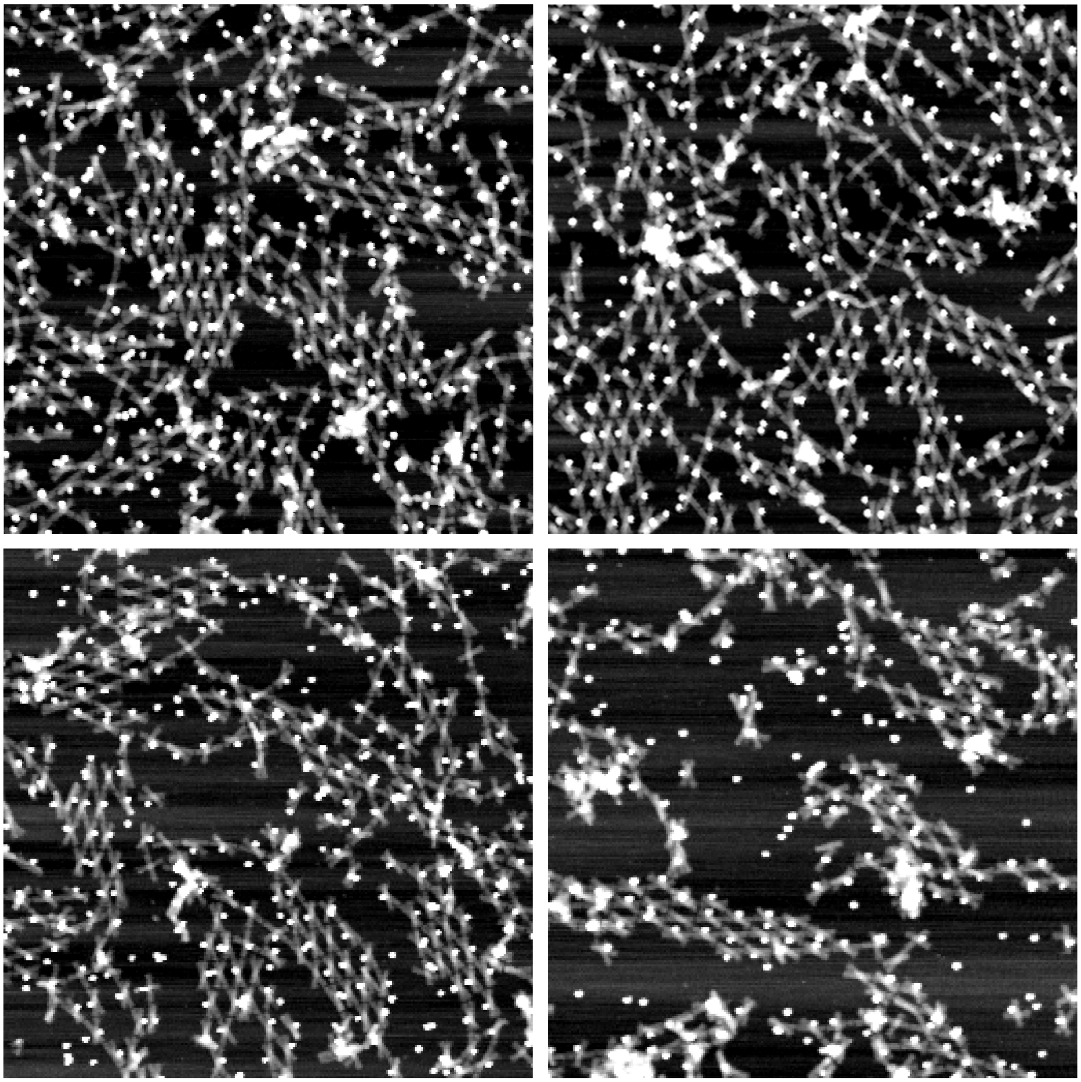

Figure S42. AFM images of AuNP lattices formed on a mica substrate at pH 6.0 using the DNA origami unit with pH latches. The assembly is done in 1× TAE containing 12.5 mM $MgCl_2$ and 75 mM NaCl. The size of the images is 1.5 μm × 1.5 μm. The DNA origami concentration is 3.0 nM (calculated based on the concentration in the conjugation step, assuming no loss during the PEG purification).

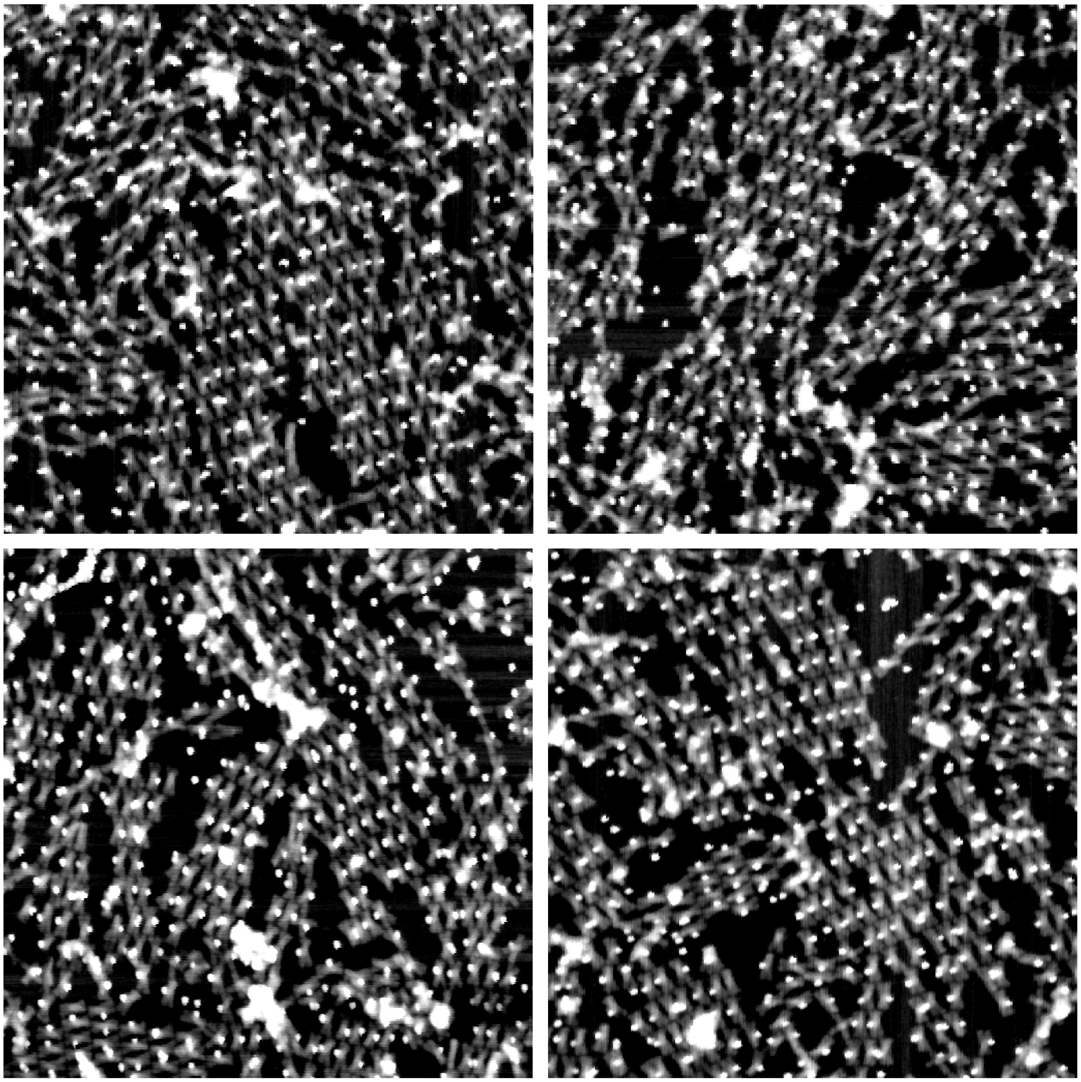

Figure S43. AFM images of AuNP lattices formed on a mica substrate at pH 8.2 using the permanently closed unit. The assembly is done in 1× TAE containing 10 mM $MgCl_2$ and 75 mM NaCl. The size of the images is 1.5 µm × 1.5 µm. The DNA origami concentration is 3.0 nM (calculated based on the concentration in the conjugation step, assuming no loss during the PEG purification).

8. DNA Origami Unit Design

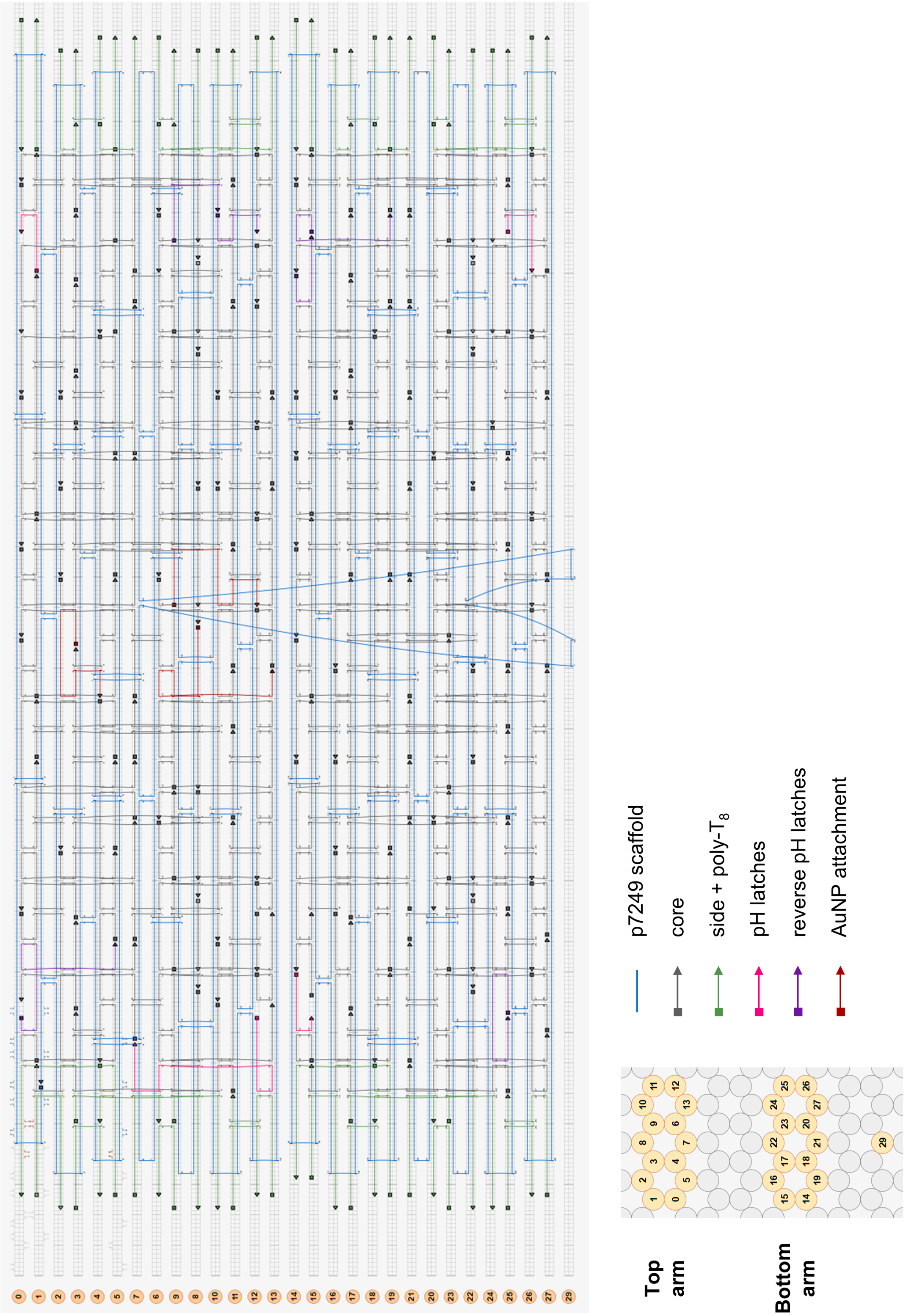

Figure S44. The caDNAno design for the DNA origami unit. The unit shown is the poly-T passivated (8-nt polythymine extensions at each helix to avoid end-to-end stacking) version and the staple strands are colored based on their type and/or function.

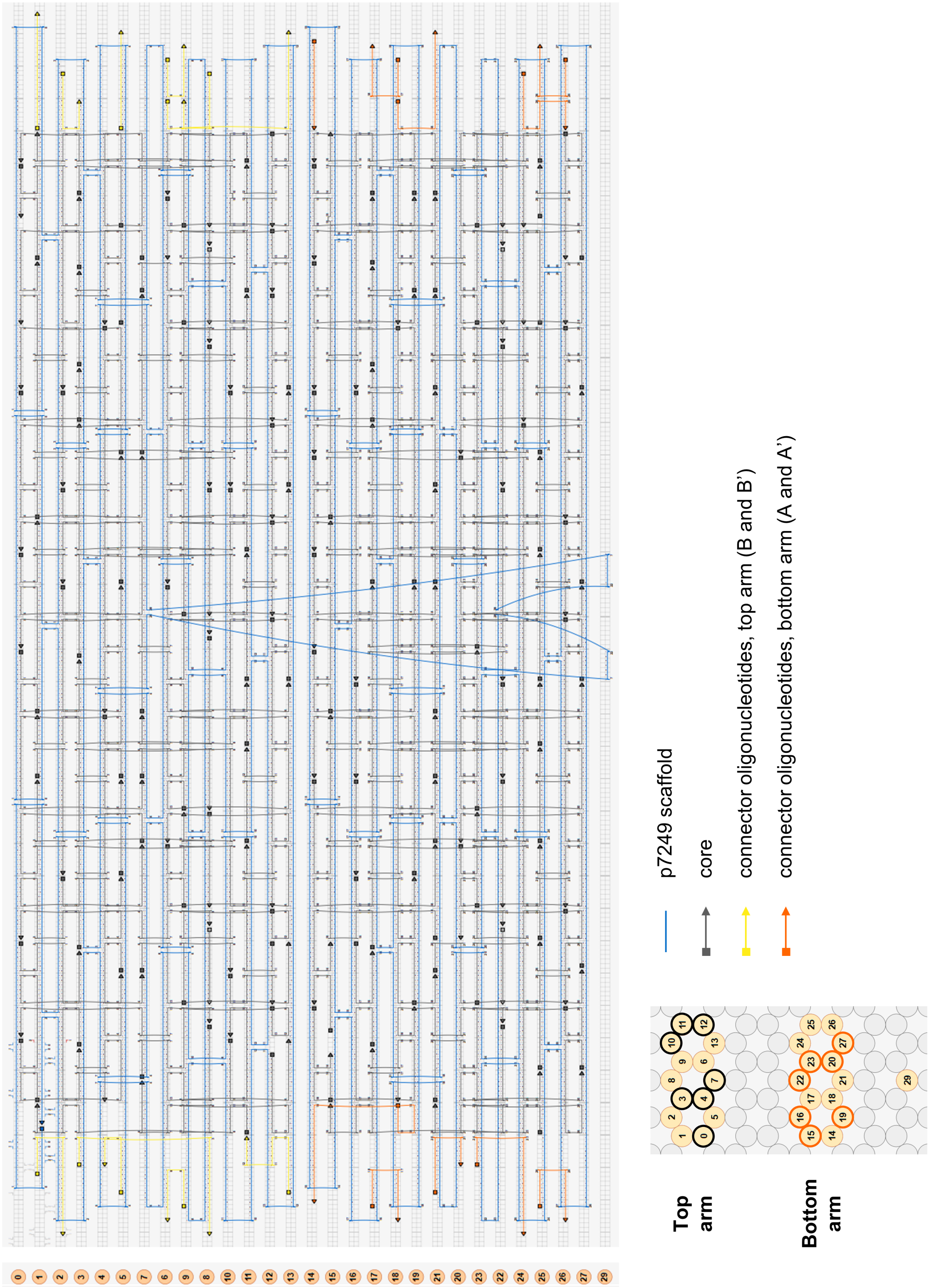

Figure S45. The design and position of the connector oligonucleotides used to selectively link the units together. Each of the two arms have connector oligonucleotides on seven of the helices and these helices are indicated in the helix map shown in the bottom-right part of the figure. 14 (7 per arm) of the connector oligonucleotides have a 3-nt overhang (in the 3' end) complementary to the scaffold sequence on the opposite end of the arm.

All versions of the DNA origami unit contain the core staple strands listed in **Table S2**. Depending on the unit type and wanted functionality, some of the core staple strands in **Table S3** could be replaced with staple strands in **Table S4** (pH latches), **Table S5** (sequences for permanently closing the unit), **Table S6** (reverse pH latches) or **Table S7** (AuNP attachment strands). The pH latches and the sequences for permanently closing the unit has been adapted from Ijäs et al. (3). The side strands with the poly-T_8 overhangs are listed in **Table S8**. For selectively connecting several units together, these side strands could be replaced with the connector oligonucleotides in **Table S9**. The replacement is always done for the whole set of staple strands of the same type, for example, the whole set "A of poly-T passivated side-strands" would always be replaced with the whole set "bottom arm, A".

Table S2. The sequences for the core staple strands. The staple strands are written in the 5' to 3' direction and the Start – End location refers to the position in the caDNAno design.

Type	Start - end	Sequence
core	0[146] – 5[160]	CGATTTTAAACAGGGAAGCCCTGAATCTATCCAAAGCAAATC
core	0[167] – 2[161]	TATCCCATACCAACCCAGCTA
core	0[202] – 3[207]	TTAACGCTTCATCAATATAATATTATCAATTTT
core	0[251] – 3[244]	GCAAAAGGCCTGATCATCGGGAGAAACACTTAGAT
core	0[83] – 3[81]	GTTTGGATTCCGAAAGGCGAAAATCCTGAATCAGAGCGGG
core	1[119] – 1[118]	TTTACAGACCCTGAACAAAGTCAGAGGGTCAAAAGAACAGCC
core	1[133] – 3[144]	AACATAATTGTTTACATTACCGCGCCCAATAGCAAACAAG
core	1[175] – 5[188]	AGCGTCTACAGCCATATCCGGTATTCTA
core	1[49] – 3[60]	TGTAGCGACGTGGACGCGGGGAGAGGCGTAAAGGGGCTTT
core	10[181] – 12[175]	TCAGCTACAGACGAGCAGAGG
core	10[202] – 13[202]	TATGCGTTATACACCGCGTTAAATAAGAGCGGAACCAGTGCC
core	10[62] – 10[63]	TGTTAACCTACATTACACGACCAGTAAGCTTGCATGTGAAAT
core	10[76] – 12[70]	TTCCTGTGCCTGCAACGACGG
core	11[105] – 6[105]	GAATCGGTGCGGGCAGTTGGGTAACGCCAGCCCTATACACCG
core	11[126] – 1[132]	CTATTACAGGCTGCCCAGGCAATCGCACCAGAGAGACTGAACAGAGAAT
core	11[140] – 11[139]	GGCGAAGTACCGACCAACAATAGATATCGCCATTCGCCAGCT
core	11[168] – 1[174]	TTCTGTCATGCAGAGGTCACGCTGCCAGCGGGAGGTTTGCACGCTAACG
core	11[224] – 8[234]	GTAGGTCTAAAATACTTTACACAACTCGTGAC
core	11[252] – 1[258]	AACAACTTGAGGATATCGCAAATTTTAGTTTTCAGCCTTTTATGCTTTG
core	11[42] – 1[48]	ACCAGTCTTGACGCAAACGCTTGGTAATAGCACGTCGCTTAACAGCAAG
core	12[153] – 0[147]	AGAATATGCAGAAGTACGAGCCCGAAGCCAATGAAATAGCAATAAGAAA
core	12[174] – 0[168]	CATTTTCAGGCGGTGTAATGGTAACAACCTCCCGAGAAGGCTTATTATT
core	12[195] – 10[182]	AACATGTCCTGCAAAAAACAAACGGCGGAGATGGGAACATGT
core	12[223] – 7[223]	ATTGAGAATCGCCAGAGCCAGAACATTAAGCGGAATTATCCA
core	12[237] – 6[231]	GGAAGGTTCTAAAGATTAATT
core	12[258] – 0[252]	TCAACAGACCTCAAATGCAAATAACCTCAATTGCGAACCTACTACCTGA
core	12[69] – 0[63]	CCAGTGCAATATTTCATAAAGGAACGGTGGCCGATGTTTGCGTCCACTA
core	12[83] – 8[87]	TTGTAAAGGTCGACTCTAGAGATGGTCAAATTAACCATC
core	12[90] – 0[84]	CACGACGGTCTTTATCCATCAAGAAGTGTTCACCGGTGGTTTTGTTCCA
core	13[140] – 7[132]	ACCACCAAAAAGGGGGATGTGCTGCAAGAAAATACCAATAATTATTAAA
core	13[182] – 12[196]	AACACCGAATTTAGCGACAATAAAAATTCTTACCACAACGCC
core	13[203] – 8[213]	ACGCTGATATTTAAGTATAAAGCCAACGCCTGTTTCCGGAATAAAT
core	14[118] – 22[119]	CTAGCGGTGGCATCAATTCTAGGGCGCGTGAACGGTACCAGA
core	14[146] – 19[160]	ATCACCACTATTTTTGAGAGATAAAAATCCGGAGAAATCATA
core	14[167] – 17[160]	AGAAAGGTTTTAGATTTATTTCAACGCATGTACCC
core	14[188] – 21[181]	AGGTAAATAAATGCAATACTTTTGCGGGAAAACATGCATAAATATTTTG
core	14[202] – 17[207]	ATTTTGTCCGCGACCTGCTCCGATAAATTCATC
core	14[251] – 17[244]	CTTGAGACAAAGCTATATTCATTACCCAAATGCAG
core	15[133] – 22[140]	AGGGTAGTCAATATTAGTAGCATTAACAAAAACAGGCATGTCAAGCAGA
core	15[175] – 14[189]	TATATTTGATTCAAAATTAGCAAAATTAAGCAAATAATGTGT
core	15[49] – 17[60]	TACAGGATCTGAATGTCATAGCCCCCTTGAAGGATTTATT

core	15[64] – 14[70]	GTAAGAGCCGCCGGCCAGAA
core	16[118] – 16[119]	GTCTGGAGCAAAGACAGACGATTTGATAAATTAATCCTGAGA
core	16[139] – 14[119]	CTATCAGAAAACTAGATATTTTCATTTGCTAATAGGATATTCAACCGTT
core	16[202] – 16[203]	CAACATTATTACGTAATGCCTGAAAGAACTGGCTCAACGGAA
core	16[83] – 17[81]	ACCAGAACCACCACGAAAGTATTAAG
core	16[97] – 21[104]	CCCTCAGAGAACCGCGCCTCCTGCCGTCGAGAGGG
core	17[161] – 27[160]	CGGTTGTTAGCCAGCTTTCAACATTCCAGATTCCCCGGTTTA
core	17[196] – 21[202]	GAAAGATTGTGTCGCGCGAAA
core	17[208] – 19[223]	AGTTGAGATACGAACTATTATACCAGTCAGCATTGTGTTAGCCG
core	17[230] – 18[217]	ATTCAACTAAAAAAATCTACGTTAATAAATTAGGAAAGGTATC
core	17[245] – 22[238]	ATACACGCGGAATCGTCATAA
core	17[61] – 18[49]	CTGAAACATCATAAGTTTTAACGGGGTCAGGAACCTATATCAAG
core	17[82] – 19[97]	GCCACCCTCAGCCGCCTGACAGGAGGTTGAAACAAATAAATCAC
core	18[216] – 14[203]	ATCGCCTATGTTACAATTACCTTATGCG
core	19[161] – 21[160]	CAGGCAACGGATAATCAGAAATATTTAA
core	19[224] – 17[229]	GAACGAGGAGGCATTACCAC
core	19[245] – 21[244]	TCATAAGGGTAACGCCAAAAGATAACCC
core	19[70] – 21[76]	TTTGCCATCTTTTCCCAGGCTGAGACTCGCTCAGT
core	19[98] – 16[98]	CGGAACCAGAGCGGCCTTGATATTCACAGGCAGGTGCCACCA
core	2[160] – 8[154]	CAATTTTAAGCCCAATAATTGTTTGAGGGGACGAC
core	2[181] – 7[188]	TTGCTATTTTTGAAAGCGAACCCGTCGGATTCTCC
core	2[202] – 2[203]	TCTGTAAATCGTCAGCCTAATTTAATTTCATTTGACCTTGCT
core	2[97] – 7[104]	TGGTTTGGAGAGTTATTGCCCTTTTTATAATCAGT
core	20[104] – 26[91]	TATAGCCTAGTACCAATTATTCATTAAAAAGGTAAATAGAAA
core	20[188] – 26[175]	TTTTTGTCCATCAAATAATGCTGTAGCTAGAGCTTACAGGTC
core	21[105] – 22[91]	TTGATCGAACACCACCGGAACCCACCCTCCCTCAGAACCGCC
core	21[119] – 23[111]	TCAATAAACCATTAAACGGAATATTACGCAGTATGTTAGCGAGCAAACG
core	21[133] – 23[146]	AGCTAAGATTGTATCGAACGAGGGTCTG
core	21[161] – 26[154]	ATTGTAATTCAGTTTATAATTTTTTAAATATGCAAGGTCATTCCTTTAA
core	21[182] – 22[182]	TTAAAATTCGCAAGAATAAAGCCTCAGATATGACCAATTCGC
core	21[203] – 24[196]	CAAAGTACCCAGCGATCAAAAAGCAAAGCGGATTG
core	21[224] – 22[234]	AGAGCAACTAAAACCTCTACGAAGGCACATAT
core	21[245] – 25[251]	TCGTTTAGCAAAGAGGCAAAAACGCATAACCGATACACCCTCCAACGGC
core	21[49] – 22[66]	CAGAAGGATTAGCGTAGCAAGAATGGGAATTAGAGAGAG
core	21[77] – 27[77]	ACCAGGCCACCAGTAGCACCAAACAACTA
core	21[84] – 15[83]	GGATAAGCTCAGAGATAATCAAAATCCTCATTAAACCAGCAT
core	22[118] – 21[118]	AGGAAACCGAGGCACACAAGAGAATCGAAGCTGAACAAATGG
core	22[139] – 27[139]	TAGCCGAAAGAACTGTAGATTAAAAGGA
core	22[181] – 25[188]	GTCTGGCAGGAACGTAAATCAAGGTGAATTTCTTAAACTCCAAATTGCT
core	22[212] – 17[195]	CTTTAAACAGTTCAGAAAACGAATCTAGGTA
core	22[233] – 22[217]	TCATTGAATCCCCCTCA
core	22[65] – 27[55]	CCACCACCCTCATTTTCCCAGTACGCCGGAAAGCGGAG
core	22[86] – 22[70]	TCAGAACCGCCACCCTC
core	23[112] – 25[125]	CAATAATGATACATAATCTCCAAAAAAAACGGAATTACATAC
core	23[147] – 16[140]	GAAGTTTGAAAAGTAATCATAAGGATCTACAAAGG
core	23[217] – 26[217]	CTTTACCACTCATCCAATGACAACAACCGCCGTTTTAATTCG
core	23[238] – 24[238]	CAACCTAAAACGCATTAAACGGGTAAAA
core	23[70] – 26[70]	CCAGCAAAATCACCTATCACCGTCACCGATTCAACCCAGCGC
core	23[91] – 25[104]	GGAGGTTCGGAATATAATAATTTTTTCAACAATCAATATTGA
core	24[195] – 27[202]	CATCAATAAGAATGACCATAAATTATACTGATACC
core	25[105] – 20[105]	CGAAACGTAGAAAAAAGTTTATTTTGTCCGTTGAATTATAAG
core	25[126] – 15[132]	ATAAAGGGACTCCTTACCCAAACAAAGTTAATCGTGTCATTGGCCGGAG
core	25[140] – 25[139]	ATATATTGATAAGACTAAAGTACGGTCATGATTAATGGCAAC
core	25[168] – 15[174]	ATGGCTTCAACATGAACCAATCTTCCTGTGTACCAAGAAGCCACCCTCA
core	25[189] – 20[189]	GAAAAAGATTAAGACCAGACCGGAAGCAAACAGCTCATTAAA
core	25[217] – 24[217]	CTTCAAATATCGATCGGAACGTACGTAATGCCAGACTATTAT
core	25[252] – 15[258]	TACAGAGGTTTCCAGAGGGGGAATACTGCATAGGCGAACCGGGCTCATT

core	25[42] – 15[48]	AAAGTTTCCTGTAGTTCGTCAAGGGATATATTTCGTGCCTTGTTGATGA
core	25[84] – 22[87]	GGGAGGGGGTGAATGTACTCAACCC
core	26[153] – 14[147]	TTGCTCCATTGTATAATTCTGAAGCAAAAGCCCCATCCAATACAGTCAA
core	26[174] – 14[168]	AGGATTAGCTTTCGGCTCATTACGTTAAGCTAAATGGCAAAGAAGGGTG
core	26[216] – 21[223]	AGCTTCAGCGCCGATTTGACCCAACGGAGATTTTA
core	26[258] – 14[252]	GGATCGTTATTCGGAAGTTTTCCAGACGCAGACCAGGAACCGAATTGGG
core	26[69] – 19[69]	CAAAGACAGAAAGGTTACCATGGGTTTTCTCAAGAATTAGCG
core	26[90] – 20[84]	ATTCATATTGCGAAGGTGTAT
core	27[140] – 21[132]	GCCTTTATTAAAGAAACGCAAAGACACCAGGCTCCTAGTTTGCCTGTTT
core	27[161] – 25[167]	TCAGCTTGAGAGTATTTGCGG
core	27[203] – 22[213]	GATAGTTAAGCGAAGGAAGCCCGAAAGAAGTCAGAATCAGGTAATG
core	27[231] – 14[231]	ATCGCCCGAATACACACTATCGAATTACGCGCAGAATTTCAA
core	27[56] – 21[48]	TGAGAATAAAGACGTTAGTAAATGAATTCAGTTTCACGTCACGTAGCGA
core	27[78] – 25[83]	AAGGAATGGTTTACGATTGA
core	3[196] – 10[203]	TTAATTAGATGATGAAACCACCAGAAGGTCATTTTATAAACAAGTATCA
core	3[208] – 0[217]	CCCTTAGAATGAATAAATTACCTTTTTTAAAGAAAAC
core	3[229] – 4[217]	TAGCGATAGATATAAATCAATATATGTGAGTCCTTGAATATCAT
core	3[245] – 8[238]	TAAGAGATTAATTTCATCTTC
core	3[61] – 4[49]	CCTCGTTAGTTGTAACCACCACACCCGCCGATAACGTATTTTCC
core	3[82] – 5[97]	TGGCCCTGACCCCAGCATCGGCAAAATCCCTGAGTGTTTCTTTT
core	4[132] – 5[118]	CACTCATTAGGAATACGTCAAAAATGAAAATAGTATTTTATT
core	4[216] – 0[203]	ATTCCTGCCTGATTAAAATTAATTACAT
core	5[119] – 8[112]	TTCATCGCGAGAACCTAATATTCCAGCCAGCTTTC
core	5[161] – 6[161]	AGATATACTAAGAGCAAGAAACAAATGTGAGCGAGGACCCAT
core	5[189] – 2[182]	AGAACAACAGTTACAAAATAATTCCAGAAGATTAG
core	5[217] – 3[228]	GTTTGGATTATACTTGAATTTAAACA
core	5[238] – 6[245]	AATGGAAGGGTTAGTACGCTGAGAAGAGAGAGACTACCTTTTTCTGCCC
core	5[77] – 7[76]	CGCCAGGCCAGCTAAACAGGAACGCCAG
core	5[98] – 2[98]	CACCAGTGAGACGTGCCCGAGATAGGGTTTATAAATCCACGC
core	6[104] – 12[91]	AGTAAAAGCAATACTCGAATTCGTAATCGATCCCCTCCCAGT
core	6[160] – 11[167]	CCTAATTATAAAACAGAGGTGGAGCCAGAAAGTAA
core	6[244] – 11[251]	GAACGTTCATCACCTTGCTGATTGAAAGGAGCACT
core	7[105] – 8[91]	GAGGCTCCCGGGCAACAGCTGGCAGCAATTGATTAGTAATAA
core	7[119] – 9[111]	GAACGGGCGGCTGTCCGGAAAGCAACTGTTGGGAAGGGCGGCTTCCGCT
core	7[133] – 8[150]	CCAAGGCTATCTTAATGTAGAATAGTCCTGAACAAGACA
core	7[189] – 8[182]	GTGGGAGGCGCGAGGCGTTTTGCCTTAATCGTAAC
core	7[224] – 11[223]	AAATCATTTAAAAGACTTCGAAACAATAGAAAAAGCTCAACA
core	7[231] – 1[230]	AGGTCTGTCAATAGTCTGAATTGAAACAAACATCATGGAAAC
core	7[54] – 8[66]	TTTAGACAGTGTAAAGATTCCGCTCACAATAACT
core	7[77] – 13[83]	AATCCTGCGACGAGCCGGAAGTTGAATGGCTATTA
core	8[111] – 7[118]	CGGCACTGCGGTAATTGAGCGAAGCAAGATTCCAA
core	8[181] – 13[181]	CGTGCATTTGGTGTATTGACCCAGTATT
core	8[212] – 3[195]	ACCGACCGTGTGATAAATAAGGCAATCGCTA
core	8[233] – 8[217]	CTAAATTTAATGGTTTG
core	8[65] – 13[62]	CAAACTATCGGCCTTGCCATGGAACCTGGGGTACGTGGCACAGAC
core	8[86] – 8[70]	ACTTGCCTGAGTAGAAG
core	9[112] – 11[125]	TCTGGTGCTTTCCTAAACATCGCCATTAGCGATTACTCTTCG
core	9[217] – 12[224]	CATAATTTTTGAGTCAGCAAATGAAAAATAGCTTA
core	9[70] – 10[77]	TCCACACAACATCATAGCTGT
core	9[91] – 11[104]	CGTTGTAGAGTCTGATGCGCGAACTGATAGGGTTTGGGTACC

Table S3. The sequences for the core strands that could be replaced to get a certain functionality. The staple strands are written in the 5' to 3' direction and the Start – End location refers to the position in the caDNAno design.

Type	Start - end	Sequence
core, pH latch 1.1	12[59] – 7[53]	TAAAAGGGACATTCTGGCGTAAGAATGCCTAACTCACTGCCCG C
core, pH latch 1.2	14[69] – 15[59]	TGGAAAGCGCAGTCGTGT
core, pH latch 2.1	1[231] – 0[240]	AGTACAACGGATTCAAGAT
core, pH latch 2.2	25[240] – [26]231	GGTAGAGCAGCGAAAGACA
core, reverse pH latch 1.1	0[62] – 5[76]	TTAAAGAGTCACGCTGCGCTGATGGTGGACAAGAGTATTGGG
core, reverse pH latch 1.2	25[56] – 25[55]	TTTCCAAGGGCGACACTTGAGCCATTACTACAACGTGTCGTC
core, reverse pH latch 2.1	9[238] – 12[238]	TATTAAATCCTTCATTAGAAGTATTAGATCTTTAGGAATTGA
core, reverse pH latch 2.2	14[230] – 19[244]	CTTTAATGACGTTGGGAAGTCAACGTAATGGTTTACGGTCAA
core, AuNP attachment	3[145] – 4[133]	AATTGAGTTATGCATTAGACGGGAGAATTAATAACCCTATACCG
core, AuNP attachment	8[149] – 13[139]	GTATCGGCCTCAGGAAGAAGCGCCAACCAATCGAACGA
core, AuNP attachment	9[154] – 12[254]	GAAAAATAATATTAACGCGCCTGTTTATAAAAGGTTAATAAG

Table S4. The sequences for the pH latches. The staple strands are written in the 5' to 3' direction and the Start – End location refers to the position in the caDNAno design. The hairpin-forming extensions and the ssDNA counterparts have been marked in italic, whereas the spacers are marked with lowercase letters.

Type	Start - end	Sequence
pH latch 1.1, hairpin	12[59] – 7[53]	*AGAGAAGAAAAGAGGAAGGAttttTCCTTCCTCTTTTCTTCTCT*ttttTA AAAGGGACATTCTGGCGTAAGAATGCCTAACTCACTGCCCGC
pH latch 1.2, ssDNA	14[69] – 15[59]	TGGAAAGCGCAGTCGTGTtttt*TCTCTTCTTTTCTCCTTCCT*
pH latch 2.1, hairpin	1[231] – 0[240]	AGTACAACGGATTCAAGATtttt*CCTTTCTTTCTTTCTTCCTCttttGAGG AAGAAAGAAAGAAAGG*
pH latch 2.2, ssdNA	25[240] – [26]231	*CTCCTTCTTTCTTTCTTTCC*ttttGGTAGAGCAGCGAAAGACA

Table S5. The sequences for permanently closing the unit. The staple strands are written in the 5' to 3' direction and the Start – End location refers to the position in the caDNAno design. The ssDNA regions forming the duplex have been marked in italic, whereas the spacers are marked with lowercase letters.

Type	Start - end	Sequence
latch 1.1 - closed, ssDNA	12[59] – 7[53]	*TCCTTCCTCTAAAGAAGAGA*ttttTAAAAGGGACATTCTGGCG TAAGAATGCCTAACTCACTGCCCGC
latch 1.1 - closed, ssDNA	14[69] – 15[59]	TGGAAAGCGCAGTCGTGTtttt*TCTCTTCTTTAGAGGAAGGA*
latch 2.1 - closed, ssDNA	1[231] – 0[240]	AGTACAACGGATTCAAGATtttt*CCTTTCTTTCAAAGAAGGAG*
latch 2.2 - closed, ssdNA	25[240] – 26[231]	*CTCCTTCTTTGAAAGAAAGG*ttttGGTAGAGCAGCGAAAGACA

Table S6. The sequences for the reverse pH latches. The staple strands are written in the 5' to 3' direction and the Start – End location refers to the position in the caDNAno design. The hairpin-forming extensions and the ssDNA counterparts have been marked in italic, whereas the spacers are marked with lowercase letters.

Type	Start - end	Sequence
reverse pH latch 1.1, hairpin	0[59] – 5[76]	*AAAAGGGAGAAGAAAAGAGG*tttt*CCTCTTTTCTTCTCCCTTT T*ttttAAGAGTCACGCTGCGCTGATGGTGGACAAGAGTATTGG G
reverse pH latch 1.2, ssDNA	25[60] – 25[59]	CAAGGGCGACACTTGAGCCATTACTACAACGTGTCGTCTTT Ctttt*TTTTCCCTCTTCTTTTCTCC*
reverse pH latch 2.1, hairpin	10[244] – 12 [240]	TATTAGATCTTTAGGAATTtttt*CCCCCTTTCTTTTTTCTTCT*tttt *AGAAGAAAAAAGAAAGGGGG*
reverse pH latch 2.2, ssDNA	15[240] – 19[244]	*TCTTCTTTTTTCTTTCCCCC*ttttCGTAATGGTTTACGGTCAA
core	14[230]–15[239]	CTTTAATGACGTTGGGAAGTCAA
core	9[238] – 10[245]	TATTAAATCCTTCATTAGAAG

Table S7. The sequences for the AuNP attachment. The staple strands are written in the 5' to 3' direction and the Start – End location refers to the position in the caDNAno design. The part complementary to the thiol modified strand is marked in italic, whereas the spacers are marked with lowercase letters.

Type	Start - end	Sequence
AuNP attachment	3[145] – 4[133]	*GAAGGGAGGAAA*GTATCGGCCTCAGGAAGAAGCGCCAACCAATC GAACGA
AuNP attachment	8[149] – 13[139]	*GAAGGGAGGAAA*ttAATTGAGTTATGCATTAGACGGGAGAATTAAT AACCCTATACCG
AuNP attachment	9[154] – 12[254]	*GAAGGGAGGAAA*ttGAAAAATAATATTAACGCGCCTGTTTATAAAA GGTTAATAAG
3' thiol modified strand	–	TTTCCTCCCTTCTTT/3ThioMC3-D/

Table S8. The sequences for the side strands. The staple strands are written in the 5' to 3' direction and the Start – End location refers to the position in the caDNAno design. The poly-T_8 overhangs added to the side strands are written in lowercase letters.

Type	Start - end	Sequence
side, polyT$_8$, B	0[289] – 0[259]	ttttttttAGAGGCGAATTATTCATTTCAAT
side, polyT$_8$, B	1[259] – 1[289]	AATACCAAGTTACAAAATCGCGCtttttttt
side, polyT$_8$, B	2[282] – 3[265]	ttttttttATATACAGTAACAGTAGTTTAAC
side, polyT$_8$, B	4[265] – 7[285]	ATAAAGACGGCTTAGGTTGGGTTATAtttttttt
side, polyT$_8$, B	4[285] – 3[282]	ttttttttCGTAAAACAGAAGTCAGATGAtttttttt
side, polyT$_8$, B	5[259] – 5[285]	CATATCAAAATTATTTGCAtttttttt
side, polyT$_8$, B	6[265] – 13[285]	AATGCTGATATCAAACCCTCAATCAAtttttttt
side, polyT$_8$, B	6[285] – 9[282]	ttttttttTAACTATATGTAAACGCGAGAtttttttt
side, polyT$_8$, B	8[282] – 9[265]	ttttttttAAACTTTTTCAAATATGACAAAG
side, polyT$_8$, B	10[282] – 12[259]	ttttttttCAATAGATAATACATTAATAGATTGGCAAA
side, polyT$_8$, B	12[285] – 11[282]	ttttttttTATCTGGTCAGTTAGAGCCGTtttttttt
side, polyT$_8$, B'	1[22] – 2[15]	ttttttttAAAACCGTCTATTGCGCCGCTACAGGGCGCGtttttttt
side, polyT$_8$, B'	3[15] – 4[18]	ttttttttTACTATGGTTGCTCGTGCCAGtttttttt
side, polyT$_8$, B'	3[35] – 8[15]	TTTGACGATCCAGAACAATATTACCGtttttttt
side, polyT$_8$, B'	4[48] – 0[22]	AGTCGGGGCCAACGCTCCAACGTCAAAGGGCGAtttttttt
side, polyT$_8$, B'	5[18] – 4[35]	ttttttttCTGCATTAATGAATCGAAACCTG
side, polyT$_8$, B'	7[18] – 6[35]	ttttttttCATTAATTGCGTTGCGTGAGTGA
side, polyT$_8$, B'	9[15] – 6[18]	ttttttttCCAGCCATTGCAGCTAACTCAtttttttt
side, polyT$_8$, B'	9[35] – 10[15]	ACAGGAATCAATCGTCTGAAATGGATtttttttt
side, polyT$_8$, B'	11[15] – 12[18]	ttttttttTATTTACATTGGGATAGAACCtttttttt
side, polyT$_8$, B'	13[18] – 11[41]	ttttttttCTTCTGACCTGAAAGCCAACAGACAGATTC
side, polyT$_8$, A	14[289] – 14[259]	ttttttttGAGAAACACCAGAACGAGTAGTA
side, polyT$_8$, A	15[259] – 15[289]	CAGTGAATAAGGCTTGCCCTGACtttttttt
side, polyT$_8$, A	16[282] – 17[265]	ttttttttAGAGTAATCTTGACAATGGCTGA
side, polyT$_8$, A	18[265] – 21[285]	CGGTGTAACGATAAAAACCAAAATAGtttttttt
side, polyT$_8$, A	18[285] – 17[282]	ttttttttAGGACAGATGAACCTTCATCAtttttttt
side, polyT$_8$, A	19[259] – 19[285]	AACTGACCAACTTTGAAAGtttttttt
side, polyT$_8$, A	20[265] – 27[285]	GCAAAAGTCGCTGAGGCTTGCAGGGAtttttttt
side, polyT$_8$, A	20[285] – 23[282]	ttttttttCGAGAGGCTTTTAAAATGTTTtttttttt
side, polyT$_8$, A	22[282] – 23[265]	ttttttttAGACTGGATAGCGTCCTAATAGT
side, polyT$_8$, A	24[282] – 26[259]	ttttttttACTTTTTCATGAGGAAGCTTTGATTTTGCG
side, polyT$_8$, A	26[285] – 25[282]	ttttttttGTTAAAGGCCGCGGACTAAAGtttttttt
side, polyT$_8$, A'	15[22] – 16[15]	ttttttttCATACATGGCTTAGTAACAGTGCCCGTATAAtttttttt
side, polyT$_8$, A'	17[15] – 18[18]	ttttttttACAGTTAATGCCCAGACTGTAtttttttt
side, polyT$_8$, A'	17[35] – 22[15]	CCCTGCCGCAAGCCCAATAGGAACCCtttttttt
side, polyT$_8$, A'	18[48] – 14[22]	TTTGCCTATTTTCGTTACCGTTCCAGTAAGCGTtttttttt
side, polyT$_8$, A'	19[18] – 18[35]	ttttttttGCGCGTTTTCATCGGCTTAGCGT
side, polyT$_8$, A'	21[18] – 20[35]	ttttttttAGCAGCACCGTAATCACAATGAA
side, polyT$_8$, A'	23[15] – 20[18]	ttttttttATGTACCGTAACACCATCGATtttttttt
side, polyT$_8$, A'	23[35] – 24[15]	ACTGAGTCATTCCACAGACAGCCCTCtttttttt
side, polyT$_8$, A'	25[15] – 26[18]	ttttttttATAGTTAGCGTATGGGATTTTtttttttt
side, polyT$_8$, A'	27[18] – 25[41]	ttttttttGCTAAACAACTTTCAATTCTGTAACGATCT

Table S9. The sequences for connector oligonucleotides. The staple strands are written in the 5' to 3' direction and the Start – End location refers to the position in the caDNAno design. The 3-nt long overhangs (in the 3' end) complementary to the scaffold sequence on the opposite end of the arm are written in lowercase letters.

Type	Start - end	Sequence
top arm, B	1[259] – 1[284]	AATACCAAGTTACAAAATCGCGCaaa
top arm, B	2[271] – 3[265]	TACAGTAACAGTAGTTTAAC
top arm, B	5[259] – 5[280]	CATATCAAAATTATTTGCActg
top arm, B	6[265] – 13[280]	AATGCTGATATCAAACCCTCAATCAActt
top arm, B	6[274] – 9[277]	CTATATGTAAACGCGAGAcca
top arm, B	8[271] – 9[265]	CTTTTTCAAATATGACAAAG
top arm, B'	1[33] – 2[20]	ACCGTCTATTGCGCCGCTACAGGGCGCGata
top arm, B'	3[35] – 8[20]	TTTGACGATCCAGAACAATATTACCGaaa
top arm, B'	5[29] – 4[35]	CATTAATGAATCGAAACCTG
top arm, B'	9[26] – 6[23]	GCCATTGCAGCTAACTCAtaa
top arm, B'	13[29] – 11[41]	CTGACCTGAAAGCCAACAGACAGATTC
bottom arm, A	14[278] – 14[259]	AAACACCAGAACGAGTAGTA
bottom arm, A	18[265] – 21[280]	CGGTGTAACGATAAAAACCAAAATAGagc
bottom arm, A	18[274] – 17[277]	ACAGATGAACCTTCATCAaca
bottom arm, A	24[271] – 26[259]	TTTTCATGAGGAAGCTTTGATTTTGCG
bottom arm, A	26[274] – 25[277]	AAAGGCCGCGGACTAAAGata
bottom arm, A'	17[26] – 18[23]	GTTAATGCCCAGACTGTAagg
bottom arm, A'	18[48] – 14[27]	TTTGCCTATTTTCGTTACCGTTCCAGTAAGCGTgag
bottom arm, A'	21[29] – 20[35]	AGCACCGTAATCACAATGAA
bottom arm, A'	23[35] – 24[20]	ACTGAGTCATTCCACAGACAGCCCTCact
bottom arm, A'	25[26] – 26[23]	GTTAGCGTATGGGATTTTgtt

9. References

1. Hung, A. M.; Micheel, C. M.; Bozano, L. D.; Osterbur, L. W.; Wallraff, G. M.; Cha, J. N. Large-area spatially ordered arrays of gold nanoparticles directed by lithographically confined DNA origami. *Nat. Nanotechnol.* **2010**, *5*, 121–126.
2. Nečas, D.; Klapetek, P. Gwyddion: an open-source software for SPM data analysis. *Open Phys.* **2012**, *10*, 181–188.
3. Ijäs, H.; Hakaste, I.; Shen, B.; Kostiainen, M. A.; Linko, V. Reconfigurable DNA Origami Nanocapsule for pH-Controlled Encapsulation and Display of Cargo. *ACS Nano* **2019**, *13*, 5959–5967.
